# Demystifying Acute Pain Management in the Emergency Department: A Case-Based Approach

**DOI:** 10.15766/mep_2374-8265.11339

**Published:** 2023-08-22

**Authors:** Morgan Sehdev, Jason Lewis, Antje Barreveld

**Affiliations:** 1 Third-Year Resident, Department of Emergency Medicine, Massachusetts General Hospital; 2 Assistant Professor, Department of Emergency Medicine, Beth Israel Deaconess Medical Center; 3 Assistant Professor, Department of Anesthesiology, Newton-Wellesley Hospital

**Keywords:** Acute Pain, Analgesia, Chalk Talk, Case-Based Learning, Clinical Teaching/Bedside Teaching, Emergency Medicine, Pain Management, Pain Medicine

## Abstract

**Introduction:**

Acute pain is one of the most common complaints that presents to the emergency department. Despite its ubiquity, oligoanalgesia, or the undertreatment of pain, remains a problem in medicine, possibly due to minimal dedicated pain teaching for senior medical students transitioning to residency.

**Methods:**

We designed a 2.5-hour interactive seminar for senior medical students transitioning into residency. The seminar included a chalk talk and case-based discussion, reviewed pain physiology, revisited pain assessment, and introduced pain management strategies using a novel acute pain plan to organize an analgesic approach from presentation through disposition from the emergency department. The didactic chalk talk was interwoven with a case of acute pain. Seminar materials promoted a near-peer teaching opportunity for future facilitators. Learners completed open-ended pre-/postsession knowledge assessments.

**Results:**

Data were obtained from 19 fourth-year medical students enrolled in three iterations of a preinternship course at Harvard Medical School. Prior to the seminar, learners scored an average of 23.0 out of 53.0 points (*SD* = 9.0) on the knowledge assessment, which improved to 36.6 out of 53.0 points (*SD* = 6.7) following the seminar (paired *t* test *p* < .001). Learner satisfaction data revealed a positive response to the seminar: Learners felt more confident managing pain and highly recommended the seminar's continuation in the future.

**Discussion:**

Initial data from this seminar suggest a need for and benefit of targeted pain education for senior medical students. Seminar materials can easily be adapted for learners in other departments or in early graduate medical education.

## Educational Objectives

By the end of the seminar, participating learners will be able to:

1. Summarize the differences between the four different types of pain (inflammatory, nociceptive, neuropathic, and visceral).
2. Efficiently assess a patient's pain on presentation to the emergency department.
3. Appropriately dose (including administration route, dose, and frequency) at least three opioid and three nonopioid pain medications.
4. Practice designing a basic acute pain management plan, from initial evaluation in the emergency department to discharge planning and prescribing, for one case-based scenario.
5. Identify three differences that need to be made in an acute pain plan for a patient with a history of substance use disorder as compared to a patient without a history of substance use disorder.

## Introduction

Acute pain is one of the most common complaints a physician will be asked to manage; however, medical students often do not feel prepared to manage pain or prescribe analgesics in a safe, effective manner as a resident and later as an attending physician.^1^ Multiple studies have shown that while most medical schools and residencies provide some form of pain education, pain-specific teaching may be variable, fragmented, and limited.^2–5^ Furthermore, deficiencies in pain education may self-perpetuate. A poor foundational understanding of pain as a student or trainee may later hinder teaching on pain as an attending.^6^ Today, comprehensive pain education includes recent concerns in pain medicine such as chronic pain and substance use disorder (SUD), but a foundational understanding of basic acute pain and its management is still lacking.^5^ The undifferentiated nature and frequency of acute pain presentations in the emergency department (ED) are ideal for discussions on acute pain.^7^ Learners in the ED must actively engage with the dueling forces between under- and overtreatment of pain, balancing the risks and benefits associated with oligoanalgesia^8^ and opioid overprescription,^9^ a balancing act best navigated with a basic understanding of pain pathophysiology and management options. Finally, the triage skills necessary to manage acute pain from presentation through disposition in the ED make for transferable skills that students and later trainees can apply in other rotations or practice settings given the ubiquity of acute pain.

Over the past decade, the number of pain education curricula and resources in *MedEdPORTAL* and elsewhere has surged in response to the overall limited scope of pain management, though most target learners in GME, emphasizing inpatient pain management^10^ or opioid use disorder/withdrawal management.^11^ Current UME-targeted publications emphasize pain management in palliative care^12^ and family medicine.^13^ These interventions highlight pain assessment and management specific to critically ill or dying patients and patients managing chronic pain, without focusing on assessment of acute pain in the undifferentiated patient. While *MedEdPORTAL* provides a handful of pain-related emergency medicine (EM) interventions, existing pieces are typically simulations or case-based learning scenarios utilizing a singular diagnosis (e.g., neck pain) as opposed to generalized acute pain.^14,15^ To our knowledge, there have been no prior studies or interventions designed for medical students or interns/junior residents that target general acute pain education as it pertains to emergency care or that use a chalk talk approach.

We created and evaluated a 2.5-hour, highly interactive, and unique didactic seminar on acute pain and its management in the ED. The seminar was designed for and implemented with senior medical students.^16^ It introduced relevant pain physiology, bias within pain care, and the idea of a presentation-through-disposition pain plan—a concept not featured in pain education interventions yet one particularly relevant for care in the ED and hospital setting at large. We designed the seminar utilizing Kern's frameworks for curriculum design^17^ and considering the arc of acute pain care within a fourth-year medical student's zone of proximal development (physiology = “I know,” clinical assessment/medication names = “I am learning,” prescribing and discharge considerations = “I am soon to be learning”). The material was presented in a chalk talk format to increase in-session discussion and engagement while promoting enhanced postsession retention.^18^ We assessed pre- and postseminar knowledge using patient case vignettes and open-ended questions to evaluate learners’ knowledge acquisition. The chalk talk format and pain plan framework were developed to promote our primary goal of creating an engaging and accessible educational intervention.

As a secondary goal, we created a tool kit suited for sustainable practice so that the seminar could continue to be broadly implemented. The seminar facilitation materials support a mentored near-peer teaching model that aims to empower student-educators and provide a unique, beneficial experience for near-peer learners.^19^ We also designed a facilitation primer as a novel approach to facilitate pain education by decreasing the barrier some educators may face when planning to teach about acute pain.

## Methods

This research met exemption criteria outlined by the Harvard Medical School (HMS) Program of Medical Education and Medical Education Institutional Review Board. Informed consent from students was obtained. Students were given the option to abstain from all surveys and assessments.

### Curricular Context

HMS recently introduced longitudinal curricular themes, such as those described by Kitzes and colleagues,^20^ focusing on common educational gaps identified within the curriculum. Pain and pain management were one of these curricular themes, aiming to incorporate pain teaching throughout the HMS experience in response to the national UME deficit in pain education.^21^ As a result, pain-focused seminars became incorporated into several fourth-year preinternship courses. Students were also exposed to 2 days of pain-related material during a second-year course occurring at the transition from preclinical to clinical learning. Currently, the curricular development team is working to interleave more pain-focused teaching throughout the preclinical courses themselves.

Fourth-year medical students at HMS had the opportunity to participate in two different preinternship preparatory courses within the Department of Emergency Medicine at Beth Israel Deaconess Medical Center. The first course was the Emergency Medicine Bootcamp, an optional advanced elective open to students applying to EM residencies or preliminary intern years. The second course, the Emergency Medicine Capstone, was required after applying for residency in EM. Both courses included didactic sessions and simulations focused on preparing students for the transition to intern year. Reflecting on prior iterations of the two courses and HMS's new pain and pain management theme, we recognized a lack of formal teaching on acute pain and its management in the ED throughout the curricula of both courses. Considering the curricular theme's mission as our general needs assessment^17^ for the HMS student body, we developed and implemented this seminar in response. Educational objectives for the intervention were informed by medical committee recommendations for acute pain management curricula in UME^22^ and EM-specific GME.^4^

### Participants and Target Learners

Fourth-year medical students enrolled from February 2021 through April 2022 in the bootcamp and capstone courses described above were required to participate in the acute pain management seminar during prescheduled didactics. Both courses fostered small-group learning within all learning sessions. Subsequently, we designed the seminar to be effective in smaller groups, although the materials could be adapted to meet the needs of larger learning groups. The content of our seminar required basic familiarity with pain signaling and assessment. Our content was most appropriate for a learner with clinical clerkship experience. Though our curriculum was implemented with senior medical students applying in EM, the material would also be suitable and adaptable for medical students or junior residents in other specialties.

### Educational Intervention

We developed a highly interactive, 2.5-hour seminar to be incorporated into both courses (note that the material presented in the session could, at facilitator discretion, be spread over multiple shorter sessions). The seminar featured an instructional chalk talk (Appendix A) interwoven with an extended case-based problem (Appendix B). The learners were not required to complete any prereading but were given a 10-minute presession knowledge assessment (Appendix C). To avoid oversurveying the learners, the presession knowledge assessment also served as a targeted needs assessment^17^ (any weak points for a particular group of learners given their pooled presession responses could be addressed in greater detail during their seminar). The assessment tools were designed collaboratively by all three authors to implement open-ended, case-based questioning that asked learners to develop a pain plan; the design was informed by best practices in medical education.^23^

The first 40 minutes of the seminar were split between covering the didactic material demonstrated during the first of the two chalk talk board maps (i.e., the depictions of the chalk talk; Appendix A) and covering the first half of the case-based scenario (Appendix B). The chalk talk facilitated a discussion that contextualized pain signaling and types of pain (i.e., nociceptive, inflammatory, neuropathic, visceral) with a particular emphasis on why this physiology could greatly impact management. Additionally, the material highlighted why managing acute pain was important on a physiologic and psychologic level for the patient. This segment concluded by reviewing pain assessment, placing emphasis on pain rating scales and pain-focused physical exams using trauma-informed care. Upon completion of the first chalk talk board map, learners were introduced to a patient case: Ms. C, a 41-year-old, Spanish-speaking woman with a history of inflammatory bowel disease who presented with several hours of right-sided abdominal pain (Appendix B).

The patient case played a supporting role by creating a space to discuss, practice, and apply the material covered during the chalk talk.^24^ Specifically, learners practiced collecting a pain-focused history, conducting a pain-focused exam, and identifying the type of pain experienced by the patient. Learners were also encouraged to practice naming special considerations that could globally impact the patient's pain care, as patient features including age,^25^ gender,^26^ and ethnicity^27^ have all been shown to play a role in pain-related health disparities.

The next 80 minutes of the seminar reviewed the material provided on the second chalk talk board map (Appendix A). This segment introduced and reviewed 11 common analgesics. Learners started this portion of the seminar by independently conducting guided research on an assigned analgesic (e.g., morphine). Learners were asked to specifically research analgesic dosing parameters, uses/indications, and side effects/contraindications. Learners returned to the group to report their findings as 15-minute experts on their assigned analgesics. The facilitator then filled the second chalk talk board as directed by the learners, adding detail or clarification as necessary. Upon completion of the analgesics table, the facilitator transitioned to the second half of the patient case. We utilized the 15-minute expert exercise here for several reasons: (1) Learners could practice and troubleshoot navigating suggested on-shift resources, (2) facilitators could introduce an opportunity for active learning utilizing a break-as-action moment,^28^ and (3) facilitators could encourage future retention (e.g., learners recalling, “I remember telling the group _____ about this analgesic” or “My colearner taught us _____ about another analgesic”). Those opting not to include the 15-minute expert exercise could consider incorporating additional mini-vignettes and having learners apply the knowledge presented to them after the facilitator has filled in the table.

**Figure 1.**
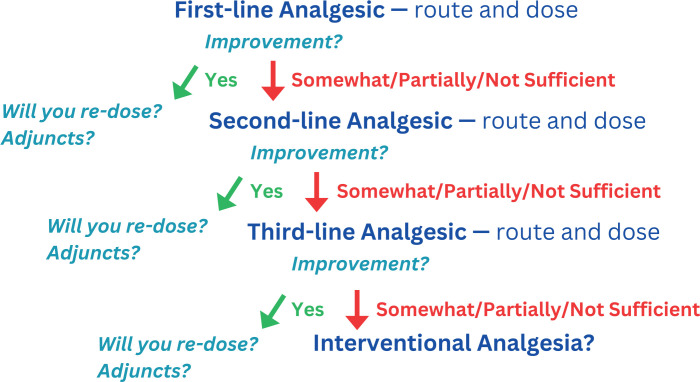
Recommended approach to developing a pain plan. This stepwise progression should be immediately considered after every encounter and mapped out mentally or on a scrap of paper to eliminate the mental burden of asking oneself, “What have I tried? What will I try next?”, if notified by the patient or other staff later in one's shift that the initial analgesic did not have much effect for the patient. Depending on one's practice pattern, select a first line using a pain ladder, shelf, or pyramid approach for escalation of analgesia.

Upon reintroducing the case, the facilitator defined and described the concept of a pain plan (Figure 1). Adapted from chronic and cancer pain management,^29^ the acute pain plan asked learners to consider first-, second-, and third-line interventions for pain from presentation through disposition (i.e., home or to the floor/ICU from the ED). We identified a paucity of teaching materials for UME and GME learners emphasizing discharge planning from the ED and therefore wanted to highlight this aspect through the case. Learners practiced developing an acute pain plan for Ms. C from presentation to discharge through guided, open-ended questions. The learners then briefly considered alterations to the pain plan if Ms. C were a pediatric patient, geriatric patient, or patient with SUD. On concluding the case discussion, the facilitator returned to the whiteboard and summarized an approach to discharge planning and constructing patient-centered pain plans.

**Figure 2.**
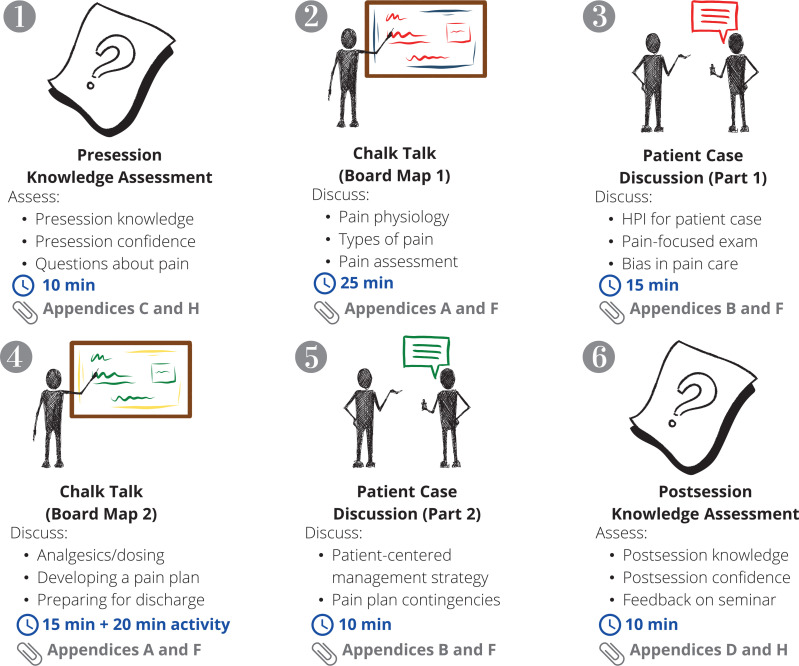
Graphical outline of the seminar's structure. The seminar can be broken down into six unique elements, including the administration of the pre- and postseminar knowledge assessments. The two board maps that anchor the seminar's 2.5 hours assist facilitators in capturing critical learning points while also serving as memory aids for the learners. Abbreviation: HPI, history of present illness.

The session concluded with a 10-minute postsession knowledge assessment (Appendix D). Students were given a pocket card (Appendix E) summarizing the material covered during the seminar and were provided with additional information on acute pain management. Figure 2 offers a visual representation of the workflow through the intervention's various phases.

### Facilitator Resources

Author Morgan Sehdev designed the seminar's materials as a part of a peer-to-peer/near-peer initiative within HMS's pain curricular theme. The three iterations of the seminar were delivered by Morgan Sehdev as a fourth-year medical student peer-to-peer facilitator and then as an intern and second-year resident near-peer facilitator, a strategy that promoted learner/facilitator self-efficacy, response efficacy, and cognitive congruence (communicating in the same language as learners). Because the seminar was designed to allow for a near-peer facilitation strategy, we developed a detailed facilitator guide (Appendix F) that included instructions on how to use the session materials and facilitation suggestions. While the facilitator for our seminars was a fourth-year medical student and subsequently a junior resident, the material was designed to support other medical students, residents, or junior attendings seeking to deliver the seminar to either medical student or resident learners. In addition to the guide, we constructed a facilitator primer (Appendix G) summarizing the literature on pain and pain management from authors in both pain medicine and EM. The primer provided facilitators who might feel hesitant to teach on pain with the necessary context to facilitate the seminar. Additional references were listed in the primer should a facilitator desire further reading. Whether used as a starting point or a refresher, the primer served to promote increased pain education.

### Assessment and Evaluation

The pre- and postsession knowledge assessments (Appendices C and D) served as the primary subjective and objective assessment of the seminar. Both assessments surveyed learners’ confidence in triaging and managing a patient with acute pain on a 5-point Likert scale (1 = *not at all confident,* 5 = *extremely confident* [ready to manage independently as an intern]). Additionally, the assessments provided two patient case vignettes. Two or three open-ended questions accompanied the vignettes to evaluate learners’ ability to name the type of pain experienced by a patient; develop a pain plan with a first-, second-, and third-line analgesic; provide discharge instructions for patients to manage pain at home; and consider how a pain plan would change if the patient had a history of SUD. We scored the open-ended questions using the coding scheme found in the annotated knowledge assessment (Appendix H), which assigned 1 point for each correct piece of information in a given response. The postassessment included subjective questions eliciting learner feedback to improve future iterations of the seminar. Pre- versus postintervention data for the seminars were analyzed using paired Student *t* tests.^30^

## Results

From February 2021 through March 2023, 19 fourth-year medical students enrolled in either the Emergency Medicine Bootcamp (*n* = 15) or the Emergency Medicine Capstone (*n* = 4) course and completed the pre- and postsession knowledge assessments (a total of four different course offerings/learner enrollments). One hundred percent of learners who participated agreed to complete the assessments and did so. Ten of the 19 students applied into EM, and six enrolled in preparation for a medicine/pediatrics preliminary year.

Rated on a 5-point Likert scale (1 = *not at all confident,* 5 = *completely confident*), learner confidence in determining an appropriate or adequate pain plan to manage patient pain while in the ED increased significantly from a presession mean of 2.5 (*SD* = 0.7, *n* = 19) to a postsession mean of 4.0 (*SD* = 0.5, *p* < .001). Additionally, learner confidence in determining a second-line analgesic if the patient had minimal relief from the first attempt at pain management also increased significantly (*n* = 19; presession *M* = 2.2, *SD* = 0.7; postsession *M* = 4.1, *SD* = 0.6; *p* < .001).

We assessed change in learner knowledge after the seminar by comparing the scores from the five open-ended questions. Student scores demonstrated a significant increase in knowledge following the seminar. Out of 53 possible points, the average cumulative score before the seminar was 23.1 (*SD* = 9.0), while afterward, the average cumulative score was 36.6 (*SD* = 6.3, *p* < .001). Scores for each of the five open-ended questions individually increased significantly after the seminar (Table 1). Notably, as a result of the seminar, learners’ ability to identify the types of pain experienced in a patient vignette and the breadth of appropriately chosen second- and third-line analgesic options increased significantly. Qualitatively, students commented that the seminar increased their “familiarity with doses of most commonly used pain medications” and that they now understood the “four different types of pain and potential ways to target [each].” Students identified individual learning gaps following the seminar by listing remaining questions; for example, “When should I consider medications like gabapentin for neuropathic pain?” and “When do I skip systemic pain medications and start an interventional pain management strategy such as a nerve block?”

**Table 1.**
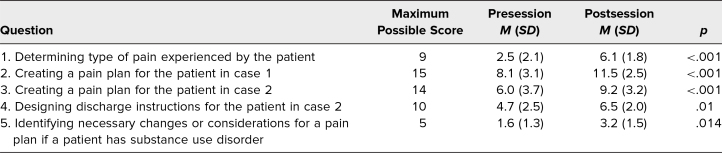
Learner Scores on Pre- and Postsession Knowledge Assessments (*N* = 19)

The seminar was highly rated by the learners, with an average rating of 4.8 on a 5-point Likert scale (1 = *not at all helpful,* 5 = *extremely helpful*). All learners felt strongly that they would recommend repeating the session for other students in the future, with an average rating of 4.9 on a 5-point Likert scale (1 = *would not recommend at all,* 5 = *would highly recommend*).

Given the unique nature of applying a peer-peer and subsequently near-peer teaching model, we have also included observations and lessons learned by lead author Morgan Sehdev in Table 2. In future iterations at HMS, where other near-peers will fulfill the role of facilitator, we plan to develop facilitator assessments to further guide development and improvement of the seminar.

**Table 2.**
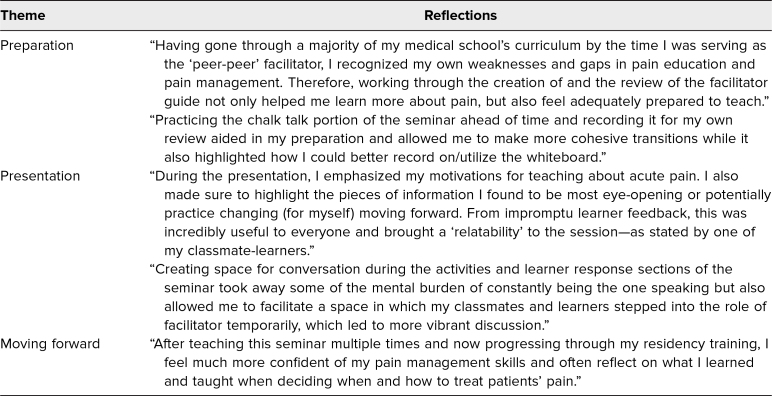
Perspectives From the Peer-Peer Teacher

## Discussion

We developed our interactive chalk talk and case-based seminar in response to the limited, fragmented, and variable teaching medical students and residents may receive on pain.^2–5^ The seminar was well received by our learners while significantly enhancing both their confidence and their knowledge. As physicians continue to address ongoing public health crises related to pain care (e.g., the opioid epidemic or SUD) through medical education,^12^ it remains necessary to bolster trainees’ basic understanding of acute pain management.^5^ Our seminar not only increases the availability of acute pain educational interventions but also provides ample resources to enhance future facilitators’ comfort with pain education. This unique seminar gives learners the opportunity to review acute pain physiology, assessment, and analgesic pharmacology while introducing the novel idea of an acute pain plan from presentation through disposition, as well as the disparities within pain care.

Important lessons learned during the creation and implementation of this seminar include the following: (1) When utilizing the near-peer teacher approach, ensure that the student or trainee facilitator is supported by a more senior mentor both prior to and during the seminar, as this mentor can help the facilitator navigate the material and provide in-seminar clarification for any learner questions that extend beyond the facilitator resources or possible cognitive congruence; and (2) review the pharmacologic management table used on the second chalk talk board map with your institution's pharmacist to confirm that the medications, doses, and routes of administration are likely to be encountered or used in practice by your learners.

A strength of this intervention is the approach to acute pain demonstrated in the seminar's progression—a framework that fosters a stepwise discussion through types, characterization, and management of patients’ pain using our novel acute pain plan approach. The acute pain plan and associated knowledge assessment were developed after recognizing the lack of a framework for helping trainees learn about and consider acute pain from presentation through disposition. This framework can be applied outside of the ED, such as in inpatient medical and surgical services. By encouraging trainees to anticipate the need for repeated dosing of analgesics or to consider second- or third-line analgesics, we can promote improved patient care, comfort, and outcomes.

Although innovative and filling a known educational need, our intervention had limitations: (1) We implemented our seminar at a single institution among a limited cohort of learners—it is encouraging that the results were significant among our small cohort, but they may not be generalizable to other institutions or learners; (2) our assessment strategy primarily evaluated students’ reactions to and knowledge acquired during the seminar, as the assessments were not able to account for any possible behavior changes in practice; and (3) the seminar was limited to 2.5 hours and did not allow for a full discussion of adjunct medications that might be useful for certain types of pain (e.g., steroids, gabapentin, muscle relaxants) or interventional pain control and nerve blocks, which are increasingly common in the ED.

Moving forward, modifications will be made to the teaching materials to focus on the theory of pain ladders, pyramids, and/or multimodal approaches, especially as they pertain to the development of a pain plan. These modifications will be made in response to some of the learners’ weaker responses to the postsession knowledge assessment. Furthermore, this seminar can be adapted and implemented among different learners. We anticipate that future iterations will allow other near-peer teachers to practice, prepare, and present the material. Further work with this seminar will continue to evaluate change in learners’ knowledge while also seeking an evaluation strategy to measure behavioral changes, particularly among resident learners. For future resident learners, the addition of templated discharge pain plans for patients discharged home with acute pain will be critical. Adding adjunct simulation to the seminar may promote possible behavioral changes among future learners and move the seminar further up Bloom's taxonomy.^31^ To assess knowledge retention or behavior changes, facilitators presenting this material to students who will also be evaluated in a clinical component of the course might consider including a question regarding pain management skills on a faculty postshift feedback form. Finally, this material would benefit from complementary seminars that address the acute pain management topics not covered in detail during the seminar, including nerve blocks, opioid conversions, and neuropathic pain-specific treatment.

This seminar ultimately addressed a curricular gap experienced by senior medical students, provided learners with an engaging near-peer chalk talk that enhanced their confidence in and knowledge of managing acute pain, and demonstrated a framework with which to manage a critically common complaint in medicine.

## Appendices


Chalk Talk Board Maps.docxPatient Case.docxPresession Knowledge Assessment.docxPostsession Knowledge Assessment.docxPocket Card.pdfFacilitator Guide.docxFacilitator Notes and Prereading.docxAnnotated Knowledge Assessment.docx
*All appendices are peer reviewed as integral parts of the Original Publication.*

## References

[1] Yanni LM, McKinney-Ketchum JL, Harrington SB, et al. Preparation, confidence, and attitudes about chronic noncancer pain in graduate medical education. J Grad Med Educ. 2010;2(2):260–268. 10.4300/JGME-D-10-00006.1 21975631 PMC2930316

[2] Mezei L, Murinson BB; Johns Hopkins Pain Curriculum Development Team. Pain education in North American medical schools. J Pain. 2011;12(12):1199–1208. 10.1016/j.jpain.2011.06.006 21945594 PMC13235945

[3] Shipton EE, Bate F, Garrick R, Steketee C, Shipton EA, Visser EJ. Systematic review of pain medicine content, teaching, and assessment in medical school curricula internationally. Pain Ther. 2018;7(2):139–161. 10.1007/s40122-018-0103-z 30058045 PMC6251835

[4] Paziana K, Timpano E, Stolbach A. Designing and implementing emergency department pain management curriculum: a Delphi approach. AEM Educ Train. 2018;2(2):121–129. 10.1002/aet2.10092 30051079 PMC6001602

[5] Vadivelu N, Mitra S, Hines R, Elia M, Rosenquist RW. Acute pain in undergraduate medical education: an unfinished chapter! Pain Pract. 2012;12(8):663–671. 10.1111/j.1533-2500.2012.00580.x 22712557

[6] Dotters-Katz S, Hargett CW, Zaas AK, Criscione-Schreiber LG. What motivates residents to teach? The *Attitudes in Clinical Teaching* study. Med Educ. 2016;50(7):768–777. 10.1111/medu.13075 27295481

[7] Todd KH. A review of current and emerging approaches to pain management in the emergency department. Pain Ther. 2017;6(2):193–202. 10.1007/s40122-017-0090-5 29127600 PMC5693816

[8] Rupp T, Delaney KA. Inadequate analgesia in emergency medicine. Ann Emerg Med. 2004;43(4):494–503. 10.1016/j.annemergmed.2003.11.019 15039693

[9] Axeen S, Seabury SA, Menchine M. Emergency department contribution to the prescription opioid epidemic. Ann Emerg Med. 2018;71(6):659–667.e3. 10.1016/j.annemergmed.2017.12.007 29373155

[10] Lester P, Remolana R, Sandhu S, Scott J. Road map for opioid management in the inpatient setting: a structured approach to opioid selection and titration. MedEdPORTAL. 2016;12:10470. 10.15766/mep_2374-8265.10470 31008248 PMC6464480

[11] Fujita AW, LaRosa A, Carter A. Treating withdrawal and pain in inpatients with opioid use disorder: a brief educational intervention for internal medicine residents. MedEdPORTAL. 2021;17:11123. 10.15766/mep_2374-8265.11123 33768154 PMC7970646

[12] Sagin A, Kimberly SM, Farabelli JP, Schafer K, Kumar P, Uritsky TJ. Teaching pain management in serious illness in the era of the opioid epidemic: a team-based intervention. MedEdPORTAL. 2020;16:11006. 10.15766/mep_2374-8265.11006 33150202 PMC7597940

[13] Smith K. Introduction to pain management for third-year medical students team-based learning module. MedEdPORTAL. 2021;17:11095. 10.15766/mep_2374-8265.11095 33598538 PMC7880255

[14] Barratt D, Tolchin R, Brown A, Segui D, Nausheen F. Neck pain OSCE: a 65-year-old patient presents to the emergency department with neck pain. MedEdPORTAL. 2015;11:10151. 10.15766/mep_2374-8265.10151

[15] Riddell JC, Sawtelle S, Jhun P, et al. Low back pain in the emergency medicine department: a flipped classroom module. MedEdPORTAL. 2016;12:10458. 10.15766/mep_2374-8265.10458 31008236 PMC6464443

[16] Teo AR, Harleman E, O'Sullivan PS, Maa J. The key role of a transition course in preparing medical students for internship. Acad Med. 2011;86(7):860–865. 10.1097/ACM.0b013e31821d6ae2 21617513 PMC3128667

[17] Thomas PA, Kern DE, Hughes MT, Tackett SA, Chen BY, eds. Curriculum Development for Medical Education: A Six-Step Approach. 4th ed. Johns Hopkins University Press; 2022.

[18] Mookherjee S, Beste LA, Klein JW, Wright J, eds. Chalk Talks in Internal Medicine: Scripts for Clinical Teaching. Springer; 2020.

[19] Hall S, Harrison CH, Stephens J, et al. The benefits of being a near-peer teacher. Clin Teach. 2018;15(5):403–407. 10.1111/tct.12784 29573152

[20] Kitzes JA, Savich RD, Kalishman S, et al. Fitting it all in: integration of 12 cross-cutting themes into a school of medicine curriculum. Med Teach. 2007;29(5):437–442. 10.1080/01421590701288564 17885970

[21] Antman KH, Berman HA, Flotte TR, Flier J, Dimitri DM, Bharel M. Developing core competencies for the prevention and management of prescription drug misuse: a medical education collaboration in Massachusetts. Acad Med. 2016;91(10):1348–1351. 10.1097/ACM.0000000000001347 27532868

[22] Howard B. Students learn to treat pain, with and without opioids. Association of American Medical Colleges. October 2, 2018. Accessed July 6, 2023. https://www.aamc.org/news-insights/students-learn-treat-pain-and-without-opioids

[23] Hauer KE, Boscardin C, Brenner JM, van Schaik SM, Papp KK. Twelve tips for assessing medical knowledge with open-ended questions: designing constructed response examinations in medical education. Med Teach. 2020;42(8):880–885. 10.1080/0142159X.2019.1629404 31282798

[24] Pluta WJ, Richards BF, Mutnick A. PBL and beyond: trends in collaborative learning. Teach Learn Med. 2013;25(suppl 1):S9–S16. 10.1080/10401334.2013.842917 24246112

[25] Platts-Mills TF, Esserman DA, Brown DL, Bortsov AV, Sloane PD, McLean SA. Older US emergency department patients are less likely to receive pain medication than younger patients: results from a national survey. Ann Emerg Med. 2012;60(2):199–206. 10.1016/j.annemergmed.2011.09.014 22032803 PMC3338876

[26] Chen EH, Shofer FS, Dean AJ, et al. Gender disparity in analgesic treatment of emergency department patients with acute abdominal pain. Acad Emerg Med. 2008;15(5):414–418. 10.1111/j.1553-2712.2008.00100.x 18439195

[27] Pletcher MJ, Kertesz SG, Kohn MA, Gonzales R. Trends in opioid prescribing by race/ethnicity for patients seeking care in US emergency departments. JAMA. 2008;299(1):70–78. 10.1001/jama.2007.64 18167408

[28] Graffam B. Active learning in medical education: strategies for beginning implementation. Med Teach. 2007;29(1):38–42. 10.1080/01421590601176398 17538832

[29] Developing a pain control plan. American Cancer Society. Updated January 3, 2019. Accessed July 6, 2023. https://www.cancer.org/treatment/treatments-and-side-effects/physical-side-effects/pain/developing-a-pain-control-plan.html

[30] Harpe SE. How to analyze Likert and other rating scale data. Curr Pharm Teach Learn. 2015;7(6):836–850. 10.1016/j.cptl.2015.08.001

[31] Bloom BS, Krathwohl DR, Masia BB, eds. Taxonomy of Educational Objectives. David McKay; 1986.

