# Supplementary material for: Demystifying Acute Pain Management in the Emergency Department: A Case-Based Approach
Source: MedEdPORTAL. 2023 Aug 22;19:11339. doi: 10.15766/mep_2374-8265.11339 (PMC10442463; doi:10.15766/mep_2374-8265.11339)
Supplement: Supplementary file 1 — Chalk Talk Board Maps.docxPatient Case.docxPresession Knowledge Assessment.docxPostsession Knowledge Assessment.docxPocket Card.pdfFacilitator Guide.docxFacilitator Notes and Prereading.docxAnnotated Knowledge Assessment.docx [file mep_2374-8265.11339-s001.zip › D. Postsession Knowledge Assessment.docx]

**Emergency Medicine Boot Camp** Last 4 Digits of Phone Number: _________

**Pain Seminar: Post-session Knowledge Assessment**

**Managing Pain:**

1. ***After this seminar*:** When a patient presents in acute pain, how confident do you feel determining an appropriate or adequate pain plan to manage their pain while they are in the Emergency Department?

**1 (Not at all confident) 2 3 4 5 (Completely confident, intern/resident level)**

1. ***After this seminar*:** How confident do you feel determining a second line analgesic if the patient has minimal relief from the first attempt at pain management?

**1 (Not at all confident) 2 3 4 5 (Completely confident, intern/resident level)**

1. ***After this seminar*:** As you approach the beginning of intern year, what would you say is your biggest barrier or greatest weakness to providing appropriate pain care to patients in the Emergency Department?

**Case 1:**

**CC:** 56F left rib pain and left wrist pain

**HPI:** Ms. A is a 56yo woman with no significant past medical history who presents with several hours of worsening left wrist pain and left sided chest pain. She states that she was in her usual state of health when she tripped in her home and fell this morning. During the fall, she struck her left side, but not her head and denied any loss of consciousness. She initially felt some pain, but over the course of the morning her pain increased significantly and so she had her daughter bring her to the ED. Her pain is currently 8/10. The chest pain is “sore and occasionally sharp”, nonradiating and she points to the left midaxillary line near the 6th rib space; the chest pain is worsened by taking a deep breath, so she feels as if her breathing is quite shallow. Ms. A believes she fell on her outstretched wrist trying to stop her fall. The left wrist pain is “sore” and does not radiate. However, she does find it difficult to flex the wrist. The pain improves minimally during holding it still. There is mild swelling of the wrist. 400mg of Ibuprofen at home helped minimally, which is why she chose to come in as the pain was unbearable at home and her daughter was concerned about the patient’s inability to take a deep breath without being in pain. Patient’s review of systems is otherwise negative and she does not take any daily medications at home, including blood thinners.

**Exam:** General: **Uncomfortable, in mild distress**. Awake, alert, and oriented.

VS: Temp 36.9 C, **HR 105**, BP 130/85, **RR: 18**

HEENT: Normocephalic, atraumatic. No cervical spinal tenderness.

CV: Regular rate and rhythm. No murmurs, rubs, or gallops. No elevated JVP. Peripheral pulses palpable. Capillary refill intact, of note intact distal to the left wrist.

Pulm: Deep inspiration pain limited, but clear to auscultation bilaterally.

Abd: Nondistended and nontender

MSK: **Point tenderness over the anterolateral 4th/5th/6th left ribs, visible ecchymoses in the same area. Decreased wrist flexion, extension, supination, pronation - exam pain limited. Left wrist tender to palpation with mild warmth to the touch, mild erythema in the surrounding area, and mild to moderate edema, no visible deformity.**

Neuro: Motor function is normal with muscle strength 5/5, though limited due to pain on the left upper extremity. Sensation is intact bilaterally. Reflexes 2+ bilaterally.

1. From the history and physical above, what type/class of pain do you think that Ms. A is experiencing? Please cite at least one thing from the history or physical that supports your answer.
2. You develop an assessment and order your workup for Ms. A. At the same time, you develop a “pain plan” for her as she is quite uncomfortable. Please decide on the following aspects of your pain plan (*multiple options exist):
   1. Medication (indicate route of administration): ___________________________
   2. Dose: ___________________________________________________________
   3. Frequency (in anticipation of longer stay in the department): _______________
   4. Why did you select this medication? _______________________________________________________________________________
   5. Second and third line medication, route of administration, dose, frequency if on recheck in 30 minutes Ms. A has had little to no relief with the first analgesic: ____________________________________ ____________________________________

**Case 2:**

**CC:** 49M with a history of chronic low back pain presents with worsening back pain and new leg pain

**HPI:** Mr. B is a 49yo man with a history of chronic back pain complicated by prior vertebral stress fractures who presents with one day of worsening left lower back pain and leg pain. Mr. B states that his back pain has been well-controlled over the past several weeks. For the past day, however, he has experienced worsening back pain in a larger area of his low back than he is used to in the past. Rated pain at 8 or 9 out of 10. He also has left leg pain that is completely new. The left leg pain is “sharp” and radiates down the entire leg when he bends or tries to walk, at which point it feels like it’s “burning or tingling pain”, most noticeable on the outer side of the left thigh and down to the side of the left calf. He was able to initially bear weight, but that has become progressively more difficult due to the pain. The pain improves minimally during rest. When asked, he’s not sure when she first noticed it. He thinks it was roughly just before heading home from work. Mr. B tells you that he and his wife own a hardware store and he was re-organizing storage in the back of the shop yesterday. He was unable to go to work today due to pain. 400mg of Ibuprofen at home helped minimally with his worsening pain, which is why he chose to come in as the pain was unbearable at home. Otherwise, the patient has a history of diabetes treated with metformin. No allergies. Non-smoker with no prior drug use. Review of systems notable for fatigue and some recent weight loss, but otherwise negative.

**Exam:** General: **Uncomfortable, in mild distress and trying to stay still in bed yet frequently “readjusting” his position.** Awake, alert, and oriented.

VS: Temp 36.9 C, HR 80, BP 130/85, RR: 12

HEENT: Normocephalic, atraumatic. No cervical spinal tenderness.

CV: Regular rate and rhythm. No murmurs, rubs, or gallops. No elevated JVP. Peripheral pulses faint, but palpable. Capillary refill intact.

Pulm: No increased work of breathing, clear to auscultation bilaterally.

Abd: Nondistended and nontender

MSK: No pain on log roll of the right hip, mild discomfort on left. **Paraspinal point tenderness along the lumbar spine**, left greater than right. **Range of motion of the spine and left leg pain limited**. **Straight leg raise positive** (pain starts at about 40˚ off the bed and shoots down the left leg).

Neuro: Motor function is normal with muscle strength 5/5, though **limited due to pain** on the left lower extremity (graded at 4/5). Sensation is intact bilaterally, **though worsening of the “tingling” on the left during lower extremity exam**. Reflexes **1+ for the left achilles and left patellar**, otherwise 2+.

1. You develop an assessment and order your workup for Mr. B. At the same time, you develop a “pain plan” for him as he is quite uncomfortable. Please decide on the following aspects of your pain plan (*multiple options exist):
   1. Medication (indicate route of administration): ___________________________
   2. Dose: ___________________________________________________________
   3. Frequency (in anticipation of longer stay in the department): _______________
   4. Second and third line medication, route of administration, dose, frequency if on recheck in 30 minutes Mr. B has had little to no relief with the first analgesic: ____________________________________ ____________________________________
2. After gathering further history and working him up in the Emergency Department, your clinical decision making leads you to the conclusion that Mr. B’s pain is from acute lumbar radiculopathy due to a herniated disc without red flag back pain features with baseline degenerative changes. After controlling his pain in the ED and ruling out spinal epidural abscess, you decide that he is safe to discharge home. What is your discharge pain plan? Please identify **medication**, **route of delivery**, **dose**, **frequency**, and **special instructions** for his discharge worksheet.
3. Consider that in addition to the information above, Mr. B reveals that he has a history of substance use disorder. How might you modify your pain plan if Mr. B has alcohol use disorder? Or how might you modify your pain plan if he is currently on a stable dose of methadone?

**Questions for the Post-Assessment:**

1. What is one new thing you learned from today’s session?
2. What is one question you still have or now have as a result of this session?
3. How helpful did you find this session?

**1 (Not at all helpful) 2 3 4 5 (Extremely helpful)**

1. Indicate how much you agree with this statement: I would recommend repeating this session for other students.

**1 (Strongly Disagree) 2 3 4 5 (Strongly Agree)**

1. What additional comments, concerns, or suggestions would you have for this session?
