# Supplementary material for: Demystifying Acute Pain Management in the Emergency Department: A Case-Based Approach
Source: MedEdPORTAL. 2023 Aug 22;19:11339. doi: 10.15766/mep_2374-8265.11339 (PMC10442463; doi:10.15766/mep_2374-8265.11339)
Supplement: Supplementary file 1 — Chalk Talk Board Maps.docxPatient Case.docxPresession Knowledge Assessment.docxPostsession Knowledge Assessment.docxPocket Card.pdfFacilitator Guide.docxFacilitator Notes and Prereading.docxAnnotated Knowledge Assessment.docx [file mep_2374-8265.11339-s001.zip › H. Annotated Knowledge Assessment.docx]

**Emergency Medicine: Acute Pain Management**

**Seminar for Senior Medical Students and Junior Trainees**

Key:

***Suggested answers***

**Suggested scoring**

**Summary of scoring**

**Case 1:**

**CC:** 56F left rib pain and left wrist pain

**HPI:** Ms. A is a 56yo woman with no significant past medical history who presents with several hours of worsening left wrist pain and left sided chest pain. She states that she was in her usual state of health when she tripped in her home and fell this morning. During the fall, she struck her left side, but not her head and denied any loss of consciousness. She initially felt some pain, but over the course of the morning her pain increased significantly and so she had her daughter bring her to the ED. Her pain is currently 8/10. The chest pain is “sore and occasionally sharp”, nonradiating and she points to the left midaxillary line near the 6th rib space; the chest pain is worsened by taking a deep breath, so she feels as if her breathing is quite shallow. Ms. A believes she fell on her outstretched wrist trying to stop her fall. The left wrist pain is “sore” and does not radiate. However, she does find it difficult to flex the wrist. The pain improves minimally during holding it still. There is mild swelling of the wrist. 400mg of Ibuprofen at home helped minimally, which is why she chose to come in as the pain was unbearable at home and her daughter was concerned about the patient’s inability to take a deep breath without being in pain. Patient’s review of systems is otherwise negative and she does not take any daily medications at home, including blood thinners.

**Exam:** General: **Uncomfortable, in mild distress**. Awake, alert, and oriented.

VS: Temp 36.9 C, **HR 105**, BP 130/85, **RR: 18**

HEENT: Normocephalic, atraumatic. No cervical spinal tenderness.

CV: Regular rate and rhythm. No murmurs, rubs, or gallops. No elevated JVP. Peripheral pulses palpable. Capillary refill intact, of note intact distal to the left wrist.

Pulm: Deep inspiration pain limited, but clear to auscultation bilaterally.

Abd: Nondistended and nontender

MSK: **Point tenderness over the anterolateral 4th/5th/6th left ribs, visible ecchymoses in the same area. Decreased wrist flexion, extension, supination, pronation - exam pain limited. Left wrist tender to palpation with mild warmth to the touch, mild erythema in the surrounding area, and mild to moderate edema, no visible deformity.**

Neuro: Motor function is normal with muscle strength 5/5, though limited due to pain on the left upper extremity. Sensation is intact bilaterally. Reflexes 2+ bilaterally.

1. From the history and physical above, what type/class of pain do you think that Ms. A is experiencing? Please cite at least one thing from the history or physical that supports your answer. **(+9 possible points total)**

*Ms. A is most likely experiencing a combination of nociceptive and inflammatory pain.*

(+2 point if named “nociceptive”, and +2 point if named “inflammatory”).

*After the fall, she most likely has some contusions and possibly a wrist and/or rib fracture(s). Typically, nociceptive pain would follow traumatic injury such as a fall, which is revealed in her history. The visible ecchymoses demonstrate that the fall had been significant enough to cause at least that. In response to injury, the body releases pro-inflammatory markers. The physical exam demonstrates warmth, redness, and swelling of the left wrist, which would indicate that the injury in the wrist has triggered an inflammatory response that can contribute to her pain and discomfort.*

(+1 point for each of the possible reasons cited from the history and physical, in this sample answer there are 7 findings–though different answers from the vignette may be selected at which case these can be scored at the facilitator’s discretion. **The maximum a student can cite and receive credit for are +5 findings given limited vignette, positive or negative findings count**).

1. You develop an assessment and order your workup for Ms. A. At the same time, you develop a “pain plan” for her as she is quite uncomfortable. Please decide on the following aspects of your pain plan (*multiple options exist): **(+15 possible points total)**

*For initial management, there are several options:*

- *Ketorolac 10-15mg IV Q4-6H (may opt for this if she had minimal response with Ibuprofen at home.*
- *Acetaminophen 500-1000mg PO or 1000mg IV Q6H (route depends on patient PO status and how ill the student perceives the patient to be from the limited vignette).*
- *Morphine 15mg PO or 2-10mg IV (0.1mg/kg IV) Q2-4H (IV vs PO depending on if you think the patient may need to go to OR or can tolerate PO. Opioid analgesia is much more likely to be necessary as a first line if the patient has a displaced fracture or other more severe injuries. With a patient already splinting due to rib fracture-related pain, it will be necessary to monitor respiratory status closely if opioids are administered.*
- *Ibuprofen 400-600mg PO Q4-6H (The student may elect to re-dose the ibuprofen, however the vignette hints at the fact that the patient has not responded to this at home. This answer is not wrong, but practically may increase time to analgesic relief especially if the patient will require a second-line drug if Ibuprofen continues to provide little to no relief).*
- **If a medication above is named, dosed appropriately, properly administered (route), given at an adequate frequency, and provides appropriate rationale, such as “severe pain” or “nociceptive/inflammatory pain,” the student will receive +1 for each of these five aspects of the selected analgesic. *Note that student answers may differ from what’s listed above, if this is the case the facilitator scoring the responses will need to use their discretion as to what’s appropriate and may refer to the facilitator guides for appropriate dosing.***
- **Maximum of +5 points for the appropriately identified first-line medication.**

*For second-/third-line management:*

- *For those that selected either an NSAID or APAP, could suggest an opioid, such as morphine, as dosed above.*
- *For those that selected an opioid for the severity of the pain, could choose to re-dose the same opioid after 5 minutes and titrate to the dose necessary for clinically significant pain relief.*
- *Additionally, we could trial a different opioid. For instance, could consider Hydromorphone IV 0.2-1mg IV Q2-4H. If this patient were not well appearing, hypotensive, or this was a trauma you might want to consider Fentanyl IV 0.5-1mcg/kg Q15min PRN.*
- *For students that selected first line opioid, could trial multi-modal with Ketorolac 10-15mg IV Q4-6H*
- *Ketamine IV 0.1-0.3mg/kg infused over 15 min with potential repeat doses, though this varies by patient response.*
- *Could consider interventional pain procedures, like nerve blocks for the wrist and the chest wall as these are particularly useful when patients splint from rib fracture pain or there may be a fracture requiring reduction.*
- *Other analgesics may be selected, in which case the facilitator would need to score using their discretion and refer to the chart provided in the facilitator’s reading/board maps for dosing. The medications listed above are the more commonly used or evidence-based approaches to managing the pain experienced by the patient in the vignette.*
- **For each proposed second- or third-line treatment, the student receives+1 point for each of the following appropriate pieces of the analgesic plan: name of medication, route of administration, dose, and frequency. The student can receive a maximum of +4 points for each medication second-line medication and +4 points for each third-line medication, for a total of +8 cumulative points.**
- **+2 points if a student proposes an interventional procedure, no need to dose as this is not discussed during the seminar.**
- **Maximum of +10 points for the appropriately identified second-/third-line management options.**

**Case 2:**

**CC:** 49M with a history of chronic low back pain presents with worsening back pain and new leg pain

**HPI:** Mr. B is a 49yo man with a history of chronic back pain complicated by prior vertebral stress fractures who presents with one day of worsening left lower back pain and leg pain. Mr. B states that his back pain has been well-controlled over the past several weeks. For the past day, however, he has experienced worsening back pain in a larger area of his low back than he is used to in the past. Rated pain at 8 or 9 out of 10. He also has left leg pain that is completely new. The left leg pain is “sharp” and radiates down the entire leg when he bends or tries to walk, at which point it feels like it’s “burning or tingling pain”, most noticeable on the outer side of the left thigh and down to the side of the left calf. He was able to initially bear weight, but that has become progressively more difficult due to the pain. The pain improves minimally during rest. When asked, he’s not sure when she first noticed it. He thinks it was roughly just before heading home from work. Mr. B tells you that he and his wife own a hardware store and he was re-organizing storage in the back of the shop yesterday. He was unable to go to work today due to pain. 500mg of Tylenol at home helped minimally with his worsening pain, which is why he chose to come in as the pain was unbearable at home. Otherwise, the patient has a history of diabetes treated with metformin. No allergies. Non-smoker with no prior drug use. Review of systems notable for fatigue and some recent weight loss, but otherwise negative.

**Exam:** General: **Uncomfortable, in mild distress and trying to stay still in bed yet frequently “readjusting” his position.** Awake, alert, and oriented.

VS: Temp 36.9 C, HR 80, BP 130/85, RR: 12

HEENT: Normocephalic, atraumatic. No cervical spinal tenderness.

CV: Regular rate and rhythm. No murmurs, rubs, or gallops. No elevated JVP. Peripheral pulses faint, but palpable. Capillary refill intact.

Pulm: No increased work of breathing, clear to auscultation bilaterally.

Abd: Nondistended and nontender

MSK: No pain on log roll of the right hip, mild discomfort on left. **Paraspinal point tenderness along the lumbar spine**, left greater than right. **Range of motion of the spine and left leg pain limited**. **Straight leg raise positive** (pain starts at about 40˚ off the bed and shoots down the left leg).

Neuro: Motor function is normal with muscle strength 5/5, though **limited due to pain** on the left lower extremity (graded at 4/5). Sensation is intact bilaterally, **though worsening of the “tingling” on the left during lower extremity exam**. Reflexes **1+ for the left achilles and left patellar**, otherwise 2+.

1. You develop an assessment and order your workup for Mr. B. At the same time, you develop a “pain plan” for him as he is quite uncomfortable. Please decide on the following aspects of your pain plan (*multiple options exist): **(+14 possible points total)**

*For initial management, there are a couple of options:*

- *Naproxen 500mg PO Q12H*
- *Ibuprofen 400-800mg PO Q6H*
- *Ketorolac 10-15mg IV Q4-6H. In this case, there’s also the option to administer Ketorolac once while in the ED followed by other NSAIDs in the ED/ultimately at time of discharge*
- *NSAIDs are the preferred choice for attempting to treat acute back pain, opioids are not recommended unless severe, traumatic, or related to malignancy/fracture/epidural abscess (more worrisome back pain presentations or presence of “red flag” symptoms).*
- **+1 point for correct identification of each of the following aspects of the above medications: medication name, route of administration, dose, and frequency of administration.**
- **No points for opioids, this should be mentioned during the seminar when discussing indications, see facilitator notes.**
- **Maximum of +4 points for the appropriately identified first-line medication.**

*There are several second-/third-line options that learners may include:*

- *If not selected in first line:*
  - *Naproxen 500mg PO Q12H*
  - *Ibuprofen 400-800mg PO Q6H*
  - *Ketorolac 10-15mg IV Q4-6H*
- **+1 point for correct identification of each of the following aspects of the above medications: medication name, route of administration, dose, and frequency of administration.**
- *Students may recommend a host of common adjuncts for back-pain including trigger point injections, muscle relaxants (e.g., cyclobenzaprine), or neuromodulating agents (e.g., gabapentin).*
- **+1 point for each appropriate adjunct/second line option named from this list (note this is not the standard +4 points for dosing as dosing is not reviewed during the session and therefore not expected of students.**
- *Students could suggest topical NSAIDs.*
- **+1 point if the learner mentions topical NSAIDS. Dosing is not reviewed during the session and therefore if learners select topical NSAIDs it is okay to name them without dose or frequency.**
- *Learners may choose to add 500-1000mg PO Acetaminophen Q6H to regimen, as NSAID and APAP in combination have been found to be effective.*
- **+4 points if the learner chooses to add APAP and appropriately doses the medication.**
- *While not typically used in low back pain, many learners may view the acute on chronic nature of this pain as a reason to attempt dosing IV Ketamine (analgesic dosing) or IV Lidocaine, particularly in the post-session knowledge assessment after discussing these medications in the seminar. Dose: Ketamine IV 0.1-0.3mg/kg infused over 15 min with potential repeat doses, though this varies by patient response.*
- **+4 points if dosed fully with drug, route, dose, frequency OR +1 point if just named the drug.**
- *Should learners interpret this to be quite severe pain, learners may consider escalation of pain medications with lower dose, PO, immediate release morphine. However, this is not a solution and much of the evidence points away from this when managing back pain.*
- **+2 points if students appropriately doses all four elements of PO immediate release morphine or very low dose IV morphine; however, as it is discussed during the seminar that this is not the appropriate management for low back pain, the learner will not receive all +4 dosing points.**
- **Maximum +10 points for the second/third line management options.**

1. After gathering further history and working him up in the Emergency Department, your clinical decision making leads you to the conclusion that Mr. B’s pain is from acute lumbar radiculopathy due to a herniated disc without red flag back pain features with baseline degenerative changes. After controlling his pain in the ED and ruling out spinal epidural abscess, you decide that he is safe to discharge home. What is your discharge pain plan? Please identify **medication**, **route of delivery**, **dose**, **frequency**, and **special instructions** for his discharge worksheet. **(+10 possible points total)**

*These responses may also vary. Options includes:*

- *Naproxen 500mg PO Q12H for the 2-3 days following discharge with PCP follow up .*
- *Ibuprofen 400mg PO Q4H and Acetaminophen 500mg PO Q4H. Take Ibuprofen, wait two- three hours, take Acetaminophen, wait two-three hours, take Ibuprofen, and repeat the cycle as needed - not to exceed 4000mg of Acetaminophen a day and 3200mg of Ibuprofen a day. Do this for 2-3 days and then follow up with PCP.*
- *Follow up in 2-3 days is essential to continue evaluating and see if more pain medication is necessary.*
- **+1 point for correct identification of each of the following aspects of the above medications: medication name, route of administration, dose, and frequency of administration. +2 additional points if the learner provides clear instructions on how to dose the medications, when to follow up with a PCP, and/or what other non-pharmacologic options the patient may have for pain control.**
- **For pharmacologic interventions, students can receive a maximum of +6 points, either with the Naproxen or the Ibuprofen/APAP option.**

***Additional Considerations:***

- *Could consider a steroid “dose pak” - burst steroids, typically 20-50mg prednisone daily for 5 days. There is limited evidence for this and may be more beneficial in low back pain with inflammation and radicular symptoms.^^[[1]](#footnote-1)^^*
- **+1 point for naming steroids as an option.**
- *Students may recommend a host of common adjuncts for back-pain including trigger point injections, muscle relaxants (e.g., cyclobenzaprine), neuromodulating agents (e.g. gabapentin), or breakthrough dosing of PO morphine for very limited supply of 2-3 days.*
- **+1 point for each additional suggested adjunct, do not need to dose as this is not discussed in the seminar and therefore not expected of the learners.**
- *Special instructions: Rest and ice/heat as needed, could provide stretches/physical therapy suggestions/recommend massage. Avoid bed rest! Activity as tolerated.*
- **+1 point if the learner identifies any activity goals for the patient, though not expected as not discussed at length in the seminar.**
- *Could consider outpatient Physical Therapy (PT) referral. If unable to bear weight or significant concern, this patient may require initial evaluation by PT in the department prior to discharge.*
- **+1 point if the learner identifies any benefit in PT for the patient, though not expected as not discussed at length in the seminar.**
- *If pain is significantly severe, worsening, and continues to be without “red flag” symptoms, may consider surgical consult or interventional pain consult for outpatient epidural or trigger point injections.*
- **+1 point if the learner identifies a potential need for interventional pain procedure should the pain worsen or become severe, dosing or instructions not expected as not discussed at length in the seminar.**
- **Students can earn an additional +1 points for every appropriate adjunct or non-pharmacologic recommendation they make, as shown in the examples above for a maximum of +4 additional points.**

1. Consider that in addition to the information above, Mr. B reveals that he has a history of substance use disorder. How might you modify your pain plan if Mr. B has alcohol use disorder? Or how might you modify your pain plan if he is currently on a stable dose of methadone? **(+5 possible points total)**

*Patient should be screened to see if his history makes him likely to experience any adverse side effects from the Acetaminophen or Ibuprofen or if the total daily max dose should be decreased. In patients with active drug use or history of substance use, acute pain should always be treated. Patients are less likely to experience a high when in acute pain. Short courses of any pain medication are recommended. The patient, if requiring opioids, may need to have higher doses due to tolerance, however in this case opioids should still be avoided. PO opioids or opioids that cause less euphoria should be selected generally and still considered in patients with a history of substance use or substance use disorder. Patients should be continued on methadone or buprenorphine even when in acute pain, unless there is another reason to discontinue (this may be due to the amount of analgesia needed while in the hospital, etc.; this decision should be made with the hospital’s Pain Team).*

**Scoring this response can be subjective, for every point the student brings up from this sample response they will receive +1 point for a maximum of +5 points. If a student makes points not mentioned in this example, +1 point can be added for each correct consideration, as determined by the scorer using available literature/experience.**

1. Eskin B, Shih RD, Fiesseler FW, et al. Prednisone for emergency department low back pain: a randomized controlled trial. J Emerg Med. 2014;47(1):65-70. doi:10.1016/j.jemermed.2014.02.010 [↑](#footnote-ref-1)
