# Supplementary material for: Demystifying Acute Pain Management in the Emergency Department: A Case-Based Approach
Source: MedEdPORTAL. 2023 Aug 22;19:11339. doi: 10.15766/mep_2374-8265.11339 (PMC10442463; doi:10.15766/mep_2374-8265.11339)
Supplement: Supplementary file 1 — Chalk Talk Board Maps.docxPatient Case.docxPresession Knowledge Assessment.docxPostsession Knowledge Assessment.docxPocket Card.pdfFacilitator Guide.docxFacilitator Notes and Prereading.docxAnnotated Knowledge Assessment.docx [file mep_2374-8265.11339-s001.zip › B. Patient Case.docx]

**Emergency Medicine: Acute Pain Management**

**Seminar for Senior Medical Students and Junior Trainees**

***For Facilitator Reference -- In-session Patient Case:***

**CC:** 41F with fever and abdominal pain

**HPI:** Ms. C is a 41yo Spanish-speaking woman with a history of IBD who presents with several hours of right sided abdominal and side pain -- patient is seen with an interpreter. [*For the purposes of this case, we will assume that the interpreter was available for the duration of the visit and that the potential language barrier is not impairing your ability to assess the patient or understand fully the patient history/exam. For sessions that may be longer, the facilitator may alternatively include this as a barrier and discuss care options -- see Footnote.^^[[1]](#footnote-1)^^*]

The patient was in her usual state of health when she noticed a “twinge” in her right side while sitting at her desk this afternoon. This sensation comes and goes in waves and has progressively worsened from a “twinge” to a “sharp pain”, 7-9/10 in severity and radiating to the right groin. But, she is also complaining of diffuse lower abdominal pain that is also “dull and crampy.” Nothing seems to make the pain better and she cannot find a comfortable position. She is most concerned because the intermittent pain has become more constant over the past two hours. She endorses associated nausea without vomiting. The patient thinks she may have a fever, and does become diaphoretic during the waves of pain. Ms. C has not noticed any blood in her urine, though it has been darker over the past couple of days which she attributes to decreased water intake due to a stressful deadline at work.

At home, she takes twice daily extended release morphine 15mg for chronic low back pain -- she has taken this morning’s dose (it is now late afternoon/early evening). States that her pain has been well controlled on this medication, which was started on in Guatemala before immigrating. 400mg of Ibuprofen at home helped minimally with her worsening pain, which is why she chose to come in as the pain was unbearable at home.

Otherwise, the patient has a history of diabetes treated with metformin and hypertension treated with metoprolol. She was diagnosed with IBD as a teenager and is now in remission; only takes a daily multivitamin (no steroids or immune modulators). No prior surgeries. Non-smoker with no prior drug use. Sexually active with her husband. Last menstrual period was 3 weeks ago. Review of systems is otherwise notable for fatigue and some recent weight loss.

**Exam:** General: **Uncomfortable, in moderate distress, moving around in bed**. Awake, alert, and oriented.

VS: Temp 38.2 C, HR 90, BP 105/85, RR: 14

HEENT: Oropharynx clear. EOMI, PERRLA, no scleral icterus.

CV: Regular rate and rhythm. No murmurs, rubs, or gallops.

Pulm: Clear to auscultation bilaterally.

Abd: **Bowel sounds present, but decreased.** Nondistended. **Mild guarding,** **tender to deep palpation in the RLQ>LLQ. CVA tenderness on the right. No rigidity. Negative Murphy’s sign. Equivocal psoas sign.**

Neuro: CN 2-12 intact. Motor function is normal with muscle strength 5/5 throughout. Sensation is intact bilaterally. Reflexes 2+ bilaterally.

1. Pain management is improved when patients with limited english proficiency interact with providers using interpreters. Several studies demonstrate the benefit of considering barriers to receiving adequate analgesia, **one example:** Jimenez N, Moreno G, Leng M, Buchwald D, Morales LS. Patient-reported quality of pain treatment and use of interpreters in spanish-speaking patients hospitalized for obstetric and gynecological care. J Gen Intern Med. 2012;27(12):1602-1608. doi:10.1007/s11606-012-2154-x

   **One example regarding ED follow up:** Sarver J, Baker DW. Effect of language barriers on follow-up appointments after an emergency department visit. J Gen Intern Med. 2000;15(4):256-264. doi:10.1111/j.1525-1497.2000.06469.x [↑](#footnote-ref-1)
