# Supplementary material for: Demystifying Acute Pain Management in the Emergency Department: A Case-Based Approach
Source: MedEdPORTAL. 2023 Aug 22;19:11339. doi: 10.15766/mep_2374-8265.11339 (PMC10442463; doi:10.15766/mep_2374-8265.11339)
Supplement: Supplementary file 1 — Chalk Talk Board Maps.docxPatient Case.docxPresession Knowledge Assessment.docxPostsession Knowledge Assessment.docxPocket Card.pdfFacilitator Guide.docxFacilitator Notes and Prereading.docxAnnotated Knowledge Assessment.docx [file mep_2374-8265.11339-s001.zip › G. Facilitator Notes and Prereading.docx]

**Emergency Medicine: Acute Pain Management**

**Seminar for Senior Medical Students and Junior Trainees**

**Primer on Pain: Facilitator Notes and Pre-Reading**

There exists an identified need in both undergraduate and graduate medical education to enhance and increase teaching on pain. The “Acute Pain Management in the Emergency Department” seminar is intended to serve as a resource for educators, trainees, and students who are not only interested in furthering their own knowledge on pain and pain management, but the knowledge of their peers or potential junior learners. The seminar is designed to be delivered in a near-peer teaching format by residents or senior medical students to peers or junior learners, with support from faculty and mentors. Interested faculty may also choose to serve as facilitators and may opt to modify the content as they see fit.

As an accompaniment to the seminar materials, this primer serves as a collection of notes on pain management. Faculty, trainees, and students might be hesitant to serve as facilitator without prior background knowledge or teaching on pain. This hesitancy and lack of prior knowledge creates a barrier that perpetuates the minimal pain education trainees often receive. The primer was created as either an introduction to or review of pain management in the Emergency Department in order to combat the potentially low, self-perceived familiarity with pain and pain management on the part of future facilitators. As a form of “pre-reading” or in-session reference material for the seminar, the primer provides background, context, and further resources on pain management. Ultimately, this document is intended to motivate those previously hesitant to teach on pain management and support their endeavor to increase learners’ confidence and knowledge in providing evidence-based acute pain care.

The majority of this primer is a collective summary of Hogans and Barreveld’s *Pain Care Essentials*,^^[[1]](#footnote-1)^^ the new Emergency Medicine Residents’ Association (EMRA) tool, *EMRA’s Pain Management Guide,*^^[[2]](#footnote-2)^^ Tintinalli’s *Emergency Medicine* chapter 35,^^[[3]](#footnote-3)^^ and Harwood-Nuss’ *Clinical Practice of Emergency Medicine* chapter 15.^^[[4]](#footnote-4)^^ Additional citations for supplemental material are provided in the footnotes. It is advised that facilitators read and review this primer prior to the teaching session. This document may be useful to have on hand during the session for quick reference.

**Note:** This material is designed for faculty, trainees, or students in the field of Emergency Medicine, however principles of acute pain management discussed in this reading are broadly applicable to other medical and surgical specialties as the Emergency Department setting is used here as a means to discuss workup of general *acute pain*.

***Pain in the Emergency Department***

- Approximately 78% of patients in the Emergency Department present because of pain or are experiencing pain as an associated symptom; of these patients, 40% have an underlying chronic pain condition. A national survey demonstrated that 24 million adults with chronic pain visit the ED annually, of which 12 million visits are due to chronic pain exacerbations.^^[[5]](#footnote-5)^^
- A 2011 retrospective study out of one ED demonstrated that the mean triage pain score for all patients seen over a 6-month period was 7.1 (out of 10), with the most common presentations being minor injury and abdominal pain, and the most painful (rated as such on the pain scale) presentations being sickle cell crisis, back pain, and headaches.^^[[6]](#footnote-6)^^
- Why is managing acute pain important?
  - There are several physiologic changes that occur when a patient experiences acute pain, in addition to the acute distress and future risk for chronic pain (if unmanaged), acute pain can affect:
    - *Cardiovascular:* increase heart rate, blood pressure, and propensity for cardiac arrhythmia as the myocardium has an increased oxygen demand.
    - *Pulmonary:* localized pain can decrease a patient's incentive to inspire deeply which leads to atelectasis, splinting, eventual pneumonia, and potential respiratory failure. Studies have demonstrated that treating rib fracture pain with thoracic epidural anesthesia decreased cases of pneumonia and increased vent free days.^^[[7]](#footnote-7)^^
    - *Gastrointestinal:* cortisol/stress hormones and sympathetic drive from acute pain can itself cause an ileus, so avoiding opioids for GI motility reasons may still result in ileus due to poorly managed pain.
    - *Endocrine*: pain increases cortisol which in turn increases extracellular sodium, water retention, and blood glucose.
    - *MSK*: acute pain discourages patient ambulation.
    - *Neurologic*: delirium, altered mental status.
    - *Immune*: pain, stress, and inflammation will suppress immune responses.
  - Managing acute pain may lead to better care and ultimate disposition in the department with fewer readmissions or fewer discharge prescriptions.
- **Oligoanalgesia,** or the misuse of analgesics through their underprescription and thus undertreatment of pain, in the Emergency Department is a known problem. This term was coined in 1989 by Wilson and Pendleton.^^[[8]](#footnote-8)^^
  - Oligoanalgesia is thought to be the result of several factors: little pain management education during medical training, inadequate pain management quality measures for the ED, varied opinions on opioids and fears of prescribing them, and bias or disbelief in pain reporting based on various patient demographics. While pain may be the most common complaint in the ED, it is not commonly managed appropriately, for both acute and chronic pain.^^[[9]](#footnote-9)^^
  - Hesitancy in the face of the opioid epidemic and opioid abuse has contributed to providers’ poor management of pain. Nationally, there has been a rise in the frequency of opioid prescriptions, which has created increases in dependence as well as opioid overdose and associated mortality. One study found that while practices during the height of opioid epidemic rose in most outpatient settings, practices in the ED remained fairly constant, if not decreased -- of opioid prescriptions from 1996 to 2012 the percentage attributable to the ED decreased from 7.4% to 4.4% of the overall pool and only 2.4% of prescriptions in the top 5% of consumers came from ED physicians.^^[[10]](#footnote-10)^^
  - *Patient related barriers that contribute to oligoanalgesia*: Ethnicity, gender, age (very young, very old); Diminished cognitive function; Fear of medications: addiction, side effects; Acceptance of pain as being inevitable; Unwillingness to bother healthcare providers.
  - *Provider related barriers that contribute to oligoanalgesia*: Inadequate education; No objective measuring tool for pain; Accepting only pain reports that conform to our expectations; Perception of addiction and drug-seeking behavior.
  - *Provider related barriers that contribute to oligoanalgesia*: Lack of clearly articulated standards; Paucity of treatment guidelines; Fear of regulatory sanctions; Lack of healthcare provider accountability.
- In addition to the physiologic benefits of managing acute pain, there are psychological benefits as well. Patients who have their pain managed are less likely to be acutely anxious or worried while in the Emergency Department. These patients are also less likely to be chronically experiencing anxiety or depression from long term pain-related symptoms.
- In managing acute pain, there is less risk of developing dependence to the medication.^^[[11]](#footnote-11)^^ **Patients in acute pain will not get high on their analgesics**.
- The first step in managing a patient’s acute pain is listening and **validating** their pain (there is therapeutic benefit in this affirmation of pain).
- Unless the patient is made somnolent by a pain medication, providing analgesia (including opioids) does not compromise decision making capacity and should not be withheld for informed consent. Pain is mind altering and distracting, therefore pain itself can be compromising.

***Pathophysiology of Pain***

- Primary Afferent Nociceptors (PANs) -- a diverse subset of primary afferent neurons that act as the primary pathway in the peripheral nervous system’s initial pain sensing mechanisms in response to noxious stimuli. These nociceptors arise from the edge of the neural plate.
- Pain sensing peripheral nerve fibers:^^[[12]](#footnote-12)^^
  - Aβ nerve fibers: myelinatinated and large diameter nerve fibers, involved with simple touch and proprioception.
  - Aδ nerve fibers: thinly myelinated, medium diameter, conduct at a rate of 2 to 20 m/sec, and primary function is nociception (thermal, mechanical).
  - C nerve fibers: unmyelinated, small diameter, conduct at a rate of 0.5 to 2 m/sec, nociceptors (thermal, chemical, mechanical).
- Various analgesics function on different parts of the pain sensing pathway (four steps, see below). For instance, some work centrally and interfere with the interpretation of pain while others may work peripherally and dampen the initial pain signal caused by the stimuli. Therefore, it is critical to know the pain sensing pathway and where on the pathway you may be targeting with your analgesia as multimodal approaches not only consider the type of analgesic but the primary mode of action.
- **The four “steps” of pain sensing:**^^[[13]](#footnote-13)^^

1. **Transduction** = the conversion of a stimuli into an action potential at the site of the peripheral afferent nociceptors (PANS).
2. **Transmission** = PANs carry the signal to the dorsal root ganglion, through the dorsal horn where they synapse on the second order neurons, and then to the third order neurons of the central nervous system (CNS).
   - - Note: The Spinothalamic Tract carrying these pain sensing signals decussates (crosses midline) immediately at the level of entry.
3. **Perception** = interpretation of the signal by the CNS.
   - - Centrally the perception of pain is interpreted by several regions within the brain including the thalamus, cortex, limbic system, cingulate cortex, insula, and amygdala.
     - Because distracting input such as other tactile stimuli or injuries decrease the amount of processing power the thalamus, somatosensory cortex, and insula can dedicate to any one painful stimuli, distraction can decrease the perception of any one painful stimuli.
       - Ex: We’re inclined to rub or itch a region that is painful because it makes the pain “feel better”. Instead, we are creating a distraction for the CNS and decreasing the amount of pain signals that can be processed.
4. **Modulation** = control of the signal through pathways that inhibit/dampen or enhance the signal.
   - - Modulatory pathways can exist a priori or can develop in response to repeated insults, pain ‘themes’, inflammation, or epigenetic changes in gene expression patterns.
       - “+” Modulatory Pathways
         - Peripheral sensitization: the PNS develops lower activation thresholds or more spontaneous activity leading to increased pain and sensitivity. Example: hyperalgesia following sunburn -- PNS in skin has a lower temperature threshold and therefore finds warm water more painful than usual.
         - Central sensitization: lower activation thresholds and more spontaneous activity in the spinal cord neurons. This process can lead to generalized hyperalgesia or chronic pain conditions. Typically, NMDA antagonists or other neurotransmitter blockers are most effective at targeting this type of process. Nerve blocks may also be effective.
         - Supraspinal modulation: disruption, increased spontaneous activity, or lower firing thresholds in the cortical and nuclear pain perception regions of the brain.
       - “-” Modulatory Pathways
         - Inhibitory interneurons in the dorsal horn and efferent (descending) negative feedback pathways.
         - Endogenous opioid peptides that are released in anticipation of or response to pain.

- Excessive growth of PANs can lead to increased pain sensitivity and increased experiences of pain. PANs will grow in areas with high concentrations of neurotrophic factors, such as nerve growth factor (NGF). NGF is released at sites of injury and inflammation. Therefore, release of NGF and subsequent PAN growth leads to heightened sensitivity or chronic pain in injured areas of the body, especially areas of the body experiencing repeated or chronic insults.
- Alternatively, the loss of PANs or transduction/transmission processes is also dangerous, such as in diabetes or congenital syndromes, as pain sensing is an adaptive trait that protects us from acute, external threats.
- The spinothalamic tract (STT) is primarily responsible for transmission of the pain signals. The STT is divided into the neospinothalamic tract, which travels to the somatosensory cortex, and the paleospinothalamic tract, which travels to the limbic system and propagates a behavioral response to pain. This division of the STT demonstrates how complicated the actual perception of pain can be within the nervous system.
- For a useful diagram of the pain pathway, see: Ellison, Deborah L. "Physiology of pain." *Critical Care Nursing Clinics* 29.4 (2017): 397-406.

***Pain and Types of Pain***

- The IASP defines pain as an unpleasant sensory and emotional experience associated with actual or potential tissue damage or described in terms of such damage.
  - In 2019, the IASP began to consider including the term “or resembling that caused by” in reference to pain presentations that don’t necessarily have identifiable tissue damage.
- Understanding the type of pain the patient is experiencing is critical in determining the most appropriate management. ***There are 4 categories of pain***:
  - **Nociceptive**: “normal” pain sensing in response to threats in the environment.
    - Typically, acute and the result of activation of peripheral nociceptors.
    - This type of pain is typically proportional to the intensity of the stimulus, and this proportional relationship creates a linear “stimulus-response” curve that is reproducible person to person (unless individual factors make you more or less sensitive, moving your curve to the right or left respectively).
    - Occurs in response to painful heat, mechanical force, and other stimuli such as chemical or biochemical ones.
    - Treatment: for mild pain ice, rubbing (counter stimulus), and NSAIDs will be effective; for more severe pain opioids and other anesthetics may be necessary.
  - **Inflammatory**: heightened pain sensing that increases sensitivity to normal levels of touch, pressure, and position.
    - Occurs in response to inflammatory mediators under conditions of infection, late-phase injury response, or generalized inflammation.
    - Inflammatory markers will directly activate and sensitize PANs.
    - May present acutely or chronically.
    - Treatment: warm or cool the area, consider bracing or physical therapy, NSAIDs (and sometimes APAP, especially in combination) effective, disease modifying drugs useful, minimize opioids as able chronically but may have impact acutely.
  - **Neuropathic**: aberrant signalling in response to stimuli increasing the pain perceived in response to non-painful and painful stimuli.
    - Results from injury to the nerves or as an aberrant reaction.
    - Characterized by sensations of “burning”, “deep cold”, or “shock-like jolts.”
    - Treatment: distraction, CBT, pain-active antidepressants, pain-active anticonvulsants, local anesthetics, opioids only if necessary (minimize use).
  - **Visceral:** poorly localized pain that is caused by inflammation, increased pressure, and increased stretch on and in visceral structures. Acute somatic pain includes the muscles, skin, bones, tendons, and the parietal membranous surfaces. Visceral pain can occur everywhere else in the body cavities.^^[[14]](#footnote-14)^^
    - Typical examples include: vasospasmic angina pectoris, achalasia, peptic ulcer disease, pancreatitis, biliary disease, kidney stones, endometriosis, prostatitis, cystitis, colitis, appendicitis, and more.
    - Visceral pain can be functional/ “nociplastic” (ex: IBS) or secondary (ex: appendicitis).
    - Gastrointestinal pain is a great example of visceral pain, here the extrinsic afferents of the GI tract project to the spinal cord within splanchnic nerves, which contain both extrinsic afferents and sympathetic fibers, these then travel to the dorsal horn much like somatic pain through C and Aδ fibers.
      - Pain-sensing from these afferent tracks is generally “dull” and poorly localized as the afferents typically project into the positions on the homunculus of their developmental predecessors, or rather where the nerve segment originated from. The visceral afferents are segmental and extend into the deep dorsal horn laminae which all contributes to less specific localization.
      - Improved localization occurs, for instance in appendicitis, when inflammation comes in contact with the parietal peritoneum which has the more localized, dermatomal input to the pain perception centers.
      - Referred pain in the abdomen is due to the convergence of the visceral and somatic input at the dorsal horn coming from similar “locations” as perceived by the segmental visceral input and dermatomal somatic input.
- It is important to understand which of the above three categories a patient’s pain falls into, as it will help determine management and better inform your understanding of the disease process. There are several presentations that are multifaceted and present as one or more of these types of pain, which indicate some of the “gray zone” areas of managing pain. However, these multifaceted presentations also indicate why multimodal pain management is, at times, more effective.

***Evaluating Pain***

- Understand the patient’s experience and perception of the pain through pain scales.
  - Pain scales have been extensively studied and various tools to measure a patient’s pain exist.
  - It is important to note that pain scales represent the subjective experience for each individual patient. The patient in one encounter may experience an “8” on the pain scale of 1 to 10, but you cannot compare that to the “6” experienced by a different patient. Instead, the pain scale reporting by each patient helps the provider understand how much pain the patient subjectively is experiencing (“8” to “10” on the 10-point pain scale would mean severe and that patient has likely not experienced pain worse than their current pain). Additionally, the pain scale reporting helps the provider understand changes in an individual patient’s pain, whether improving or worsening during the patient’s visit.
  - The most reproducible and highly reliable scale is the Numeric Rating Scale (NRS) which has been studied robustly across populations.
    - The NRS is reliable in low-literacy populations, though not necessarily as reliable in patients that are non-English speaking.
    - **The NRS is a scale of 1 to 10 with 1-3 = mild pain, 4-6 = moderate pain, and 7-10 = severe pain.**
      - The minimum clinically meaningful change in a patient’s pain is defined as a change of 2 or more points on the pain scale after analgesia.
  - There exist more specialized pain scales, depending on the population of the patient, and there are more time intensive pain scales. Some examples include:
    - Faces Pain Scale Revised (FPS-R) - used for children
    - Wong-Baker FACES pain scale - low-literacy adults or children
    - FLACC (Faces, legs, activity, cry, consolability) - acute pain in kids 2 months to 7 years
    - CRIES (Crying, O2 saturation, blood pressure, heart rate, expression, and sleep) - pain in kids postnatal 32-week-old newborns to 6 months of age
    - PAIN-AD and MOBID-2 - Geriatric patients or those suffering from Alzheimer’s
- It is important to consider pain as a biopsychosocial phenomenon, therefore you must also consider:
  - Evaluating the psychological factors that may contribute to pain, such as anxiety and/or depression.
  - Evaluating the social aspects that contribute to or are altered due to the experience of pain, such as decreased social interaction or decreased physical activity.
- Pain is a subjective experience -- the language of pain between patients will differ, therefore instead of objectively comparing pain, use the patient’s subjective classification to gauge what “relief” will look like and to understand what may be distressing to the patient. It is essential to understand the meaning that the pain holds for the patient experiencing it to treat the pain.
- A good history and physical are critical in managing pain appropriately. Not only do the history and physical help you narrow your differential while figuring out the etiology of the pain, but they help you decipher what type of pain the patient may be experiencing or any risk factors/past medical history the patient may have that would factor into the management plan.
- Obtain a thorough pain characterization using “OPQRSTAA” (other variations of this mnemonic available or practiced) to guide your history. This approach will not only allow you to determine chronicity of the pain but may help you to further understand the type of pain (nociceptive, inflammatory, neuropathic) experienced by the patient and what management plan might be most effective.
  - “OPQRSTAA” = **O**nset, **P**rovocation, **Q**uality, **R**adiation, **S**everity, **T**iming, **A**lleviating factors, **A**ggravating factors.
  - “LMNOPQRST” -a different mnemonic for pain-related history that queries location, movement/migration, **nausea (good for visceral vs somatic pain)**, onset, provocation/palliation, quality, radiation, severity, timing.
  - Pieces of the “OPQRSTAA” history that might be indicative of certain pathologies or possible management considerations, some examples include:
    - *Quality* - “sharp and burning” might be neuropathic pain vs. “dull, crampy, intermittent” might be visceral pain.
    - *Radiation* - while determining radiation of pain can be challenging as you must distinguish if the pain is radiating or referred, this information is beneficial in that radiating pain may tell you what type of process is occurring to irritate viscera and subsequently nerves, either through irritation, inflammation, or impingement.
    - *Timing* - this information helps characterize acute vs. chronic pain (>6 months).
    - *Alleviating factors or aggravating factors* - this information may tell you where to start with pain management (ex: round-the-clock ibuprofen has provided no relief, do you need to start with something stronger or different in the ED?) or may tell you goals for discharge or disposition (Ex: Pain is worse with eating, so does the patient need a “PO challenge” prior to discharge?).
- Asking the patient about their functional status before and after the onset of pain is critical in determining how debilitating the pain is for them. For example, was the patient able to work before they started having pain, but cannot work with the pain? Could they walk, but the pain limits that? Functional status prior to and after the start of the pain provides the physician with a realistic target for pain control and possible discharge considerations. Changes in functional status also signal how severe the pain is and how disruptive it is in the patient’s day to day life.
- Consider a **pain-focused** physical exam.
  - Approach the physical as you would any trauma informed care. A significant number of patients with chronic pain have experienced some form of trauma that may make the physical exam particularly triggering.
  - Prior to testing or palpating, elicit the patient’s anxieties about the exam, i.e., the amount of pain they expect or fear, and attempt to explain why the maneuver may be useful or diagnostic and comfort them while performing the maneuver.
  - While inspecting, focus on patient gesturing or positioning as they may be indicating how severe the pain is or where they are primarily experiencing the pain.
  - During palpation, always go superficial to deep and gauge the patient’s tolerance while applying more pressure. Use the patient’s tolerance to guide the exam and document anything that is limited by severe pain.
  - In manipulating joints or assessing range of motion, rely on the symmetry of the human body.
    - Remember to evaluate active and passive range of motion, if necessary, as pain with passive range of motion may signal a joint space problem.
    - There are several provocative maneuvers that may be necessary to employ, so consider these given pertinent chief complaints (ex: trigger point for migraine, Tinel’s sign for carpal tunnel).
  - Screening neurologic exams are useful in determining any functional limitations or associated neurologic processes.
    - Assess sensation and motor function.
    - Test small nerve fibers with a sharp tip.
    - Hogan’s tapping test = using the sharp tip to test sensation tap 3 times per second over the legs and feet as this will create repeated stimulation that may reproduce possible hyperalgesia from sensitization processes of neuropathic pain.
- Your history and physical will ultimately guide your differential, but various pain presentations may warrant testing or imaging.
  - Examples for lab work include: Lyme, ANA, VRDL/RPR, RF, HgbA1c, Hepatitis C antibodies
  - Imaging is particularly useful in acute pain, but it is necessary to follow ACR appropriateness criteria and imaging guidelines.
    - Example: Imaging is not indicated in standard, atraumatic, acute low back pain without red flags.

***Pain Management***

- **Develop a pain plan.** A pain plan is a plan for analgesia throughout the course of the patient’s time in the ED and into disposition (discharge medications, pre-operative pain medication, or standing medications for the floor until the wards team can evaluate on admission) as opposed to addressing pain as needed and intermittently throughout the patient’s visit.
- The overall goal of pain management is to improve the patient’s symptoms, not necessarily cure the patient’s symptoms. Many believe that poorly controlled acute pain increases the risk of developing chronic pain later on in life. Additionally, acute pain can cause agitation, decrease a patient's attention and decision-making capacity, and affect multiple organ systems acutely (see above). Therefore, addressing a patient’s pain is as essential as diagnosing/managing other symptoms and pathologic processes.
- **Questions to ask when determining pain management plans**:
  - How intense is the pain?
  - How quickly is pain relief required?
  - Can the patient take oral medications?
  - Are there contraindications to particular agents?
  - What will be my second- and third-line options if the first analgesic does not provide relief?
  - Are there interventional pain strategies that I could use?
  - What tolerance or incomplete cross-tolerance to opioids may be factoring into the dosing?
  - How much of what medication will I prescribe and how often?
  - How can I make the plan multi-modal?
- In managing pain, **multimodal** approaches allow providers to increase analgesia while limiting toxicity.
  - Multimodal analgesia is a pharmacologic method of pain management which combines various groups of medications for pain relief. This combination can include different classes of medications, different mechanisms, and different administration routes.
  - Sogov and colleagues at Maimonides have proposed a “CERTA” (Channel Enzyme Receptor-Targeted Analgesia) approach that challenges providers to consider which of their medications used will target **each** of these three pain modulating pathways while attempting to treat acute pain.^^[[15]](#footnote-15)^^
  - For an example table of CERTA in practice, see: Cisewski, David H., and Sergey M. Motov. “Essential pharmacologic options for acute pain management in the emergency setting.” *Turkish journal of emergency medicine* 19.1 (2019): 1-11.
- Consider the **“pain ladder,”** **“pain pyramid,”** or **“pain platform.”** All of these tools help categorize pain by severity and provide considerations for how to manage the pain. Examples of these are shown in the figures below.
  - In 1986, the WHO proposed the **“pain ladder”** for pain management in patients with cancer-related pain. The ladder has since been adopted for other patient populations as well. Intended to “escalate” the potency of the drug being given for pain, the pain ladder starts with APAP and NSAIDs and as the patient’s pain severity increases the ladder adds opioids and adjuncts. Used as a reference tool for how to escalate pain care, the ladder has been quite useful.
  - For acute, severe pain in a patient with non-cancer related pain, a **“step down”** approach has been proposed in adaptations of the pain ladder (which typically advises a **“step up”** approach, i.e., start with the least potent/lowest rung medication and work up). The **“step down”** approach asserts that for patients with severe pain, you start with the most potent/highest rung medications and work down as their pain decreases.
    - For an image of the pain ladder, see: Pergolizzi, Joseph V., and Robert B. Raffa. "The WHO Pain Ladder: Do We Need Another Step?" *Practical Pain Management* 14.1 (2015).
  - As a result of the “step down” approach, the **“pain pyramid”** was introduced. Instead of serving as a step-by-step guide, the **“pain pyramid”** recommends medications based on the severity of pain experienced by the patient and the recommended analgesics to use for that severity. *The **“pain pyramid,”** and variations of the pain pyramid are most often applied to managing critically ill patients or patients in acute pain, including many patients in the Emergency Department.*
    - For an image of the pain pyramid, see: Cisewski, David H., and Sergey M. Motov. “Essential pharmacologic options for acute pain management in the emergency setting.” *Turkish journal of emergency medicine* 19.1 (2019): 1-11.
  - There have been several other pain management diagrams and approaches that have been proposed. Deciding which one to use depends on the patient population that you are treating, the setting in which you’re treating pain, and the severity of the individual patient’s pain. An example of yet another model is the **“pain platform.”** The platform asserts that for patients, particularly those with chronic pain, there should be more than one intervention at each level of increasing potency/aggressiveness of treatment.
    - For an image of the pain platform, see: Leung, Lawrence. "From ladder to platform: a new concept for pain management." *Journal of primary health care* 4.3 (2012): 254-258.
- In pain management, medications generally fall into the category of systemic vs. neuromodulating.
- Again, remember that a pain plan extends through the visit and into the patient’s disposition. **ASK YOURSELF:** What will I use for analgesia after the initial dose (i.e. second/third line options and scheduling of medications)? What will I discharge the patient with for their pain? What pain medications will this patient need while they are being transferred to the floor or the intensive care unit?

***Pharmacology of Pain Management***

- **Acetaminophen (APAP**)
  - APAP’s complete mechanism of action is unclear, however it is known to be an inhibitor of the COX-3 enzyme, a COX-1 splice variant present in the brain and spinal cord. Inhibition of this enzyme ultimately decreases the central production of prostaglandins and increases the patient’s pain threshold.
    - APAP also alters endogenous cannabinoid system via the vanilloid and cannabinoid signaling.
  - APAP crosses the blood brain barrier easily, allowing it to reach therapeutic levels in the CNS -- a property not common to other analgesics.
  - COX-3 posesses centrally mediated analgesic and antipyretic properties, but lacks robust anti-inflammatory activity; ***therefore, APAP works well as an analgesic and antipyretic, but is less effective as an anti-inflammatory***.
  - Administration/Pharmacology:
    - Dosing: PO 500-1000mg Q4-6H, PR 650mg Q6H, IV 1000mg Q6H
      - Maximum Daily Dose: 4g (3g if liver or kidney disease)
      - Typical single dose: PO 650mg
    - Duration of action: 4-6 hours
    - Frequency: Q4-6H
    - Maximum plasma concentration at 1 hour
    - t ½ = 2.4 hours, 85-98% bioavailability
    - Common indications: fever, nociceptive pain, general mild pain
    - Common side effects: nausea, vomiting, liver injury (typically at supratherapeutic doses)
    - Use caution when dosing in patients with liver or kidney disease.
    - Toxic metabolite in overdose: NAPQI -- reduced by glutathione. N-Acetylcysteine (NAC) = antidote. At toxic doses, APAP can cause acute liver failure.
- **NSAIDs (General Notes)^^[[16]](#footnote-16)^^**
  - NSAIDs are COX-1 and COX-2 inhibitors with a wide range of use in mild to moderate pain. Many NSAIDs are nonselective inhibitors, while some are selective against either of the two COX enzymes.
  - COX-1 and COX-2 are part of the arachidonic acid pathway. NSAIDs reduce the levels of prostaglandins in the central and peripheral nervous system, relieving pain, swelling and redness that results from activation of this pathway. NSAIDs inhibit prostaglandin-mediated stimulation of the hypothalamus subsequently lowering body temperature.
    - COX-1 is expressed in the gastric mucosa, kidneys, and possesses hemostatic function; it is the precursor to COX-3, however COX-1 possesses both peripheral and central targets. Analgesic and antipyretic target through inhibition.
    - COX-2 is expressed in the brain, bones, kidneys, and reproductive organs and is involved in inflammatory pathways/signalling. Inhibitors of COX-2 will relieve pain due to inflammation.
    - Inhibition of the COX-2 in the spinal cord decreases the excitability of dorsal horn neurons responsible for hyperalgesia and allodynia.
  - Aspirin = the only irreversible nonselective inhibitor of COX.
  - Of note, topical NSAIDs can be effective analgesics in MSK and dermatologic pain in patients with contraindications to systemic formulations. Topical NSAIDs possess mechanisms that are more localized yet similar to enteral and parenteral formulations. *Topical formulations have 3-10% systemic absorption.
  - *Common adverse effects of NSAIDs include:* GI symptoms/ulceration (decreased in COX-2 selective inhibition), hypertension, exacerbations of heart failure, cardiovascular thrombotic events, acute renal failure or injury, elevated hepatic enzymes.
  - Other than GI upset, more COX-2 inhibition generally leads to more risk for side effects.
  - *Common drug-drug interactions include:* NSAIDs+ACEi = hyperkalemia; NSAIDs + corticosteroids, SSRIs = increased risk for GI event; NSAIDs decrease renal clearance of lithium.
  - **There exist multiple **classes of NSAIDs**.** Not all NSAIDs have the same pharmacokinetic properties -- this leads to clinically relevant differences in response depending on the specific patient and pain pathology. If a patient lacks a sufficient response to one NSAID class, it may be worth considering an alternative class at a comparable effective dose. *These classes include: propionic acid derivatives, acetic acid derivatives, and oxicam derivatives.*
- **Ibuprofen (NSAID, propionic acid derivative)^^[[17]](#footnote-17)^^**
  - **Also known as:** Advil, Motrin
  - Administration/Pharmacology:
    - Dosing: PO 400-800 mg Q6H
      - Maximum Daily Dose: 3200mg
    - Duration of action: 8 hours
    - Maximum plasma concentration at 1-2 hours
    - t ½ = 2 hours, 100% bioavailability
    - Common indications: Rheumatoid/osteoarthritis, dental pain, dysmenorrhea, fever, headache -- mild to moderate pain.
    - Common side/adverse effects: GI bleeding, peptic ulcers, heart failure, hyperkalemia, bronchospasm
    - Analgesic Ceiling: 400mg
      - **Analgesic Ceiling** = in examining the dose-response curve, there is a point when additional increases in the dose of non-opioid medications provide no further benefit; the ceiling has been studied in several medications and will be reported as able^^[[18]](#footnote-18)^^
      - While the analgesic ceiling is at 400mg per dose, it’s thought that higher doses (600mg and 800mg) provide added anti-inflammatory properties. Therefore, pain may be reduced not from analgesia of the higher doses directly, but indirectly from decreased inflammation.
- **Naproxen (NSAID, propionic acid derivative)^^[[19]](#footnote-19)^^**
  - Administration/Pharmacology:
    - Dosing: PO 500mg Q12H or 250mg Q6-8H
      - Maximum Daily Dose: 1250mg
    - Duration of action: 8-12 hours
    - Maximum plasma concentration at 30 min to 1 hour
    - t ½ = 12-17 hours
    - Common indications: acute gout, ankylosing spondylitis, bursitis, polyarticular juvenile idiopathic arthritis, osteoarthritis, tendonitis, rheumatoid arthritis, musculoskeletal pain, primary dysmenorrhea, migraine/headache, and general inflammatory pain -- mild to moderate pain.
    - Common side/adverse effects: dyspepsia, nausea, dizziness, elevated liver enzymes, increased blood pressure, diminished renal function, rash, increased bleeding risk, and peptic ulcers.
- **Diclofenac (NSAID, acetic acid derivative)**
  - Administration/Pharmacology:
    - Dosing: PO 50-75mg Q12H
      - Maximum Daily Dose: 150mg
    - Duration of action: 8-12 hours
    - t ½ = 1-2 hours
    - Common indications: inflammatory pain, nociceptive pain, arthritis. *Especially useful if applied topically for MSK pain.*
    - Common side/adverse effects: GI upset, GI bleeding, renal dysfunction, bronchospasm, impaired wound healing.
    - Associated risk of dermatitis.
- **Indomethacin (NSAID, acetic acid derivative)^^[[20]](#footnote-20)^^**
  - Administration/Pharmacology:
    - Dosing: PO 25mg Q8H or 50mg Q8-12H
      - Maximum Daily Dose: 200mg
    - Duration of action: 6 hours
    - Maximum plasma concentration at 1-1.5 hours
    - t ½ = 2.6-11.2 hours
    - Common indications: inflammatory pain, nociceptive pain, arthritis, various cephalgias
    - Common side/adverse effects: GI upset, GI bleeding, renal dysfunction, bronchospasm, headache, dizziness, macular changes
- **Ketorolac (NSAID, acetic acid derivative)^^[[21]](#footnote-21)^^**
  - **Also known as:** Toradol
  - Administration/Pharmacology:
    - Dosing: IV 10mg Q4-6H (IM and PO versions as well, less common, but dosed equivalently)
      - Maximum Daily Dose: 60mg
    - Duration of action: 6 hours
    - Maximum plasma concentration at 30-40 minutes
    - t ½ = 5 hours, 80% bioavailability
    - Common indications: postoperative pain, renal colic, acute and chronic musculoskeletal pain, minor pain related to interventions, dental pain, labor pain, cancer pain; mild to moderate pain, especially when the patient cannot tolerate PO
    - Common side/adverse effects: somnolence, nausea, gastrointestinal pain, dyspepsia, diarrhea, headache, and dizziness
    - Analgesic Ceiling: 10mg, many studies find it equivalent or superior to opioids for various types of pain^^[[22]](#footnote-22)^^
    - Maximum IV/IM dose is 120 mg/day, but if age >65 years or weight <50 kg, then maximum dose is 60mg/day.
    - Maximum PO dose is 40mg/day.
    - **Do not administer for more than 3 days if IV, or 5 days if PO.**
- **Aspirin (analgesic and antipyretic dosing; NSAID, salicylate)**
  - Administration/Pharmacology:
    - Dosing: 325–650mg PO every 4 h; 300–600mg PR every 4–6 h
      - Max daily dose: 4g
    - Duration 4-8 hours
    - Contraindications: Hypersensitivity to NSAIDs or other salicylates; patients with asthma, rhinitis, and nasal polyps; do not use it in children or teenagers for viral infections, with or without fever as there is a risk of Reye’s Syndrome.
    - Common side/adverse effects: bleeding and bruisingGI irritation and mucosal bleeding, platelet dysfunction, tinnitus, CNS toxicity, metabolic acidosis.
    - Salicylate toxicity is possible, therefore must observe the maximum daily dose closely.
    - The dosing for anti-platelet activity is not documented here. While aspirin is an analgesic, its most common use is for its antiplatelet function.
- **Celocoxib (NSAID**, Cox-2 inhibitor)
  - Administration/Pharmacology:
    - Dosing: 400 mg initially, then 200 mg BID
      - Max daily dose: 600mg on first day of use, subsequently 400mg/day
    - Duration 8-12 hours
    - Typical uses include arthritis, gout, and dysmenorrhea.
    - May cause less GI side effects.
    - No inhibition of platelet function.
    - Contraindications: Hypersensitivity to celecoxib, sulfonamides, aspirin, other NSAIDs, or any component of the formulation; patients who have experienced asthma, urticaria, or allergic-type reactions after taking aspirin or other NSAIDs; use in the setting of CABG surgery.
- **Opioids (General Notes)**
  - “Opioids may be used in the treatment of traumatic injuries, visceral pain, vaso-occlusive crisis, and cancer related pain. Opioids mimic the body’s own analgesic system with a similar molecular structure as endogenous opioids.”^^[[23]](#footnote-23)^^
  - Opioids target and function as agonists of the mu, kappa, and delta receptors^^[[24]](#footnote-24)^^ (all G protein coupled) and affect the descending modulation of pain, primarily in the GI tract, cardiac tissue, immune system, vas deferens, and limbs.
    - Mu receptor activation promotes more significant analgesia, however it also incites more respiratory depression and more physical dependence (which are not associated with kappa and delta activation). Additionally, mu receptors are responsible for miosis, euphoria, decreased GI motility, and cough suppression.
    - Kappa receptor activation is responsible for sedation, miosis, dysphoria, and cough suppression.
    - Delta receptor activation possesses some physical dependence properties, but primarily creates an antidepressant effect and dysphoria. Additionally causes cardiovascular depression and decreased brain/myocardial oxygen demand.
  - Opioids trigger mast cell degranulation and release histamine (effect varies based on type of opioid).
  - Opioids provide the most diverse set of delivery methods, as they can be administered SubQ, IV, PO, SL, and IN.
  - There are multiple ways to classify and distinguish opioids from one another:
    - Natural, semi-synthetic, and synthetic
      - Helpful to know as “standard” drug screens will not always automatically include the synthetic and semi-synthetic compounds, but rather the metabolites of the natural compounds.
    - Strong agonists, mild to moderate agonists, partial agonists, and mixed receptor activity.
      - Full opioid receptor agonists (e.g., morphine) have no ceiling effect.
      - Partial agonists (e.g., buprenorphine) will have a ceiling effect.
    - Phenanthrenes, Phenylheptylamines, and Phenylpiperidines (chemical structures).^^[[25]](#footnote-25)^^
      - May be beneficial to consider these classes as patients develop adverse reactions or allergies to opioids: phenanthrene opioids are more likely to have cross reactivity reactions, but there is low cross reactivity as you switch between chemical classes^^[[26]](#footnote-26)^^
- **Dosing Opioids:**
  - If the patient can tolerate PO, try PO opioids first. There is less euphoria associated with PO medications. Of course, patients’ ability to take PO and pain severity may make IV administration the better option.^^[[27]](#footnote-27)^^
  - Immediate release is the preferred and suggested method for any opioid prescribed from the Emergency Department.^^[[28]](#footnote-28)^^
  - You can choose a weight-based or fixed dose method, sometimes this varies by hospital or practice. Refer to your pharmacy guidelines but be sure to know weight-based dosing and appropriate conversions.
  - While opioids are known to cause nausea and emesis in some patients (also due to the histamine release), pre-treating with antiemetics is not necessary.
  - Response to opioids: pain reduction due to opioids differs due to age, initial pain severity, and previous or chronic exposure to opioids. No data demonstrates differences in opioid response due to body mass or gender.
- **Converting Opioids^^[[29]](#footnote-29)^^:**

Morphine Sulfate

PO (mg)

**x3**

**÷3**

Morphine Sulfate

IV

Oxycodone

PO

**x1.5**

**÷1.5**

Hydrocodone

PO

**x1.5**

**÷1.5**

**x80**

**÷80**

**x4-7**

**÷4-7**

Hydromorphone

PO

**x20**

**÷20**

Hydromorphone

IV

Fentanyl

IV

*These conversions determine Morphine Milligram Equivalents. Milligrams of Morphine are multiplied/divided by potency conversions. Ex: IV Hydromorphone is 20x more potent than PO Morphine

**Equianalgesic Dosing typically determined using ***30mg PO Morphine Sulfate***.

**Image: Author Owned**

- **Morphine (Natural, strong agonist, phenanthrene)**
  - Administration/Pharmacology:
    - Dosing (in opioid naive patient):
      - PO 7.5-15mg Q4-6H
      - IV 2.5-5mg Q4-6H (dosing done at 0.05-0.1mg/kg)
        - In the Emergency Department, you may want to reassess the patient every 5-10 minutes after giving an initial dose of 0.1mg/kg IV morphine. If the patient has a good response, use that dose every 4-6H. If the patient has little to no response, titrate with an additional 2-4mg IV. Continue titrating until the patient experiences some analgesic benefit. Add the total amount the patient received to achieve an analgesic effect and use that dose every 4-6H. Monitor for respiratory depression. This method allows for appropriate dosing in the setting of higher patient tolerance.
      - **Conversion of IV:PO = 1:3**
    - Duration of action: 4-6 hours
    - Maximum plasma concentration at 60 min, 80% effect at 6 minutes
    - t ½ = 2 hours, 40% bioavailability enterally
    - Common indications: moderate to severe acute nociceptive pain, less utility in inflammatory pain.
    - Common side/adverse effects: accumulation in renal dysfunction, hypotension from histamine release, constipation, sedation.
- **Codeine (Natural, mild to moderate agonist, phenanthrene)**
  - Codeine is not biochemically active until metabolized into morphine, it therefore possesses variable properties when administered.
  - Children 12-18 are at increased risk for difficulty breathing after administration of codeine and caution must be taken with breastfeeding mothers as breast milk may be produced with higher-than-expected levels of the drug.
  - Not commonly used.
- **Hydromorphone (Semi-synthetic, strong agonist, phenanthrene)**
  - **Also known as:** Dilaudid
  - Administration/Pharmacology:
    - Dosing:
      - PO 2-4mg Q3-4H *Immediate release
      - IV 0.2-0.5mg (0.2-1mg depending on source) Q3-4H (dosing done at 0.075 0.015mg/kg)
        - Titrate in similar fashion to IV morphine above in severe pain.
      - Also available as extended release, suppository, and oral suspension.
    - Onset of action: 2-5 min (IV), 10-15 min (PO)
    - Duration of action: 4-6 hours
    - Maximum plasma concentration at 30-60 minutes
    - t ½ = 2-3 hours
    - Common indications: Severe pain, better in renal failure than morphine especially if the patient is on dialysis as the metabolites more easily filtered, larger doses of opioids required.
    - Common side/adverse effects: more lipophilic than morphine which leads to smaller doses, however nausea and constipation still common, accumulation in renal dysfunction, pruritus.
    - Hydromorphone tends to cause more euphoria. Many providers feel they are prescribing “less opioids” with smaller doses of Hydromorphone, but with equianalgesia the dose is equivalent to a larger amount of morphine.^^[[30]](#footnote-30)^^
- **Hydrocodone (Semi-synthetic, mild to moderate agonist, phenanthrene)**
  - **Also known as**: lots of brand names and various combination pills, most encountered is Vicodin (combo with acetaminophen).
  - Metabolized to hydromorphone, crosses blood brain barrier quickly→ increased risk of dependence.
  - Like oxycodone (below), hydrocodone was produced to limit decreased first pass metabolism to inactive metabolites, therefore both medications have variable effects as there are slow and rapid metabolizers.
  - Administration/Pharmacology:
    - Dosing:
      - Same potency as morphine PO
      - Hydrocodone: PO 5-10mg Q4-6H (IR)
      - Hydrocodone/APAP: 7.5mg/325mg
    - Duration of action: 4-6 hours
    - Onset at 30 minutes
    - Maximum plasma concentration at 90 minutes
    - t ½ = 3-4 hours
    - Common indications: moderate to severe acute pain
    - Common side/adverse effects: higher abuse potential, sedation, constipation, nausea
- **Oxycodone (Semi-synthetic, mild to moderate agonist, phenanthrene)**
  - **Also known as:** OxyContin, Percocet (combination pill)
  - Less potent when given neuraxially
  - Administration/Pharmacology:
    - Dosing:
      - PO 5-10mg Q4-6H (30mg morphine PO=20-30mg oxycodone PO).
      - ER tablets available as well as combination pills.
      - ER tablets ≠ ER capsules -- double check dosing.
    - Onset of action: 15 minutes
    - Duration of action: 4-6 hours.
    - Maximum plasma concentration at 60 minutes.
    - t ½ = 3-5 hours , 60-80% bioavailability enterally.
    - Common indications: moderate to severe acute pain.
    - Common side/adverse effects: there exist slow and fast metabolizers, renal toxicity.
- **Fentanyl (Synthetic, strong agonist, phenylpiperidine)**
  - Does not have the same histamine releasing properties, so useful in hemodynamically unstable patients.
  - Metabolized to inactive metabolites, so safe for use in renal dysfunction.
  - Administration/Pharmacology:
    - Dosing:
      - IV 0.35-1mcg/kg
      - 75-100x more potent than morphine
    - Duration of action: 1 hour
    - Maximum plasma concentration in minutes
    - Common indications: hypotensive patient with acute pain, trauma, indication for general anesthesia (intubation or impending surgery), concern for renal failure or risk of renal accumulation.
    - Common side/adverse effects: respiratory depression, muscle (chest wall) rigidity.
    - Preferred in patients with renal failure as less renal accumulation and easily dialyzed.
    - Transdermal fentanyl may be useful, but less so acutely as onset is delayed.
- **Tramadol (Synthetic, weak agonist and serotonin/norepinephrine reuptake inhibitor)^^[[31]](#footnote-31)^^**
  - Administration/Pharmacology:
    - Dosing: PO 50-100mg Q4-6H
      - Maximum Daily Dose: 400mg
    - Duration of action: 3-6 hours
    - Maximum plasma concentration at 1 hour
    - Common indications: mild to moderate pain, postoperative pain
    - Common side/adverse effects: serotonin syndrome, less likely to cause respiratory depression.
  - Lowers seizure threshold, therefore contraindicated in epilepsy.
  - This is a “dirty drug” that is commonly advised against using due to the variable response patients can have to it.^^[[32]](#footnote-32)^^
  - High risk for serotonin syndrome when administered with other serotonergic drugs.
  - Tramadol can produce a false-positive result on the urine phencyclidine screen.
- **Buprenorphine (Semi-synthetic, mixed agonist-antagonist, phenanthrenes)**
  - Used for both opioid use disorder and pain.
  - Partial mu agonist, partial kappa, and delta antagonist
  - If a patient is taking it, it may be contributing to some pain relief. It is difficult to dose additional opioids in addition to buprenorphine.
  - Administration/Pharmacology:
    - Dosing: Varies, analgesic ceiling at 8-16mg daily
    - Common side/adverse effects: respiratory suppression and habit formation
- **Methadone (Synthetic, strong agonist and NMDA antagonist, phenylheptylamines)**
  - Used for chronic pain and opioid use disorder.
  - Changes in dosing must occur on the order of days as it’s difficult to titrate.
  - The NMDA antagonism can reverse prior opioid tolerance.
  - Administration/Pharmacology:
    - Dosed once daily.
    - Duration of action for analgesia: 4-12 hours **Minimal effect and should not be dosed for acute pain.
    - t ½ = 6-150 hours
    - Common side/adverse effects: respiratory suppression, QTc prolongation
- **Naloxone (Semi-synthetic, opioid antagonist)**
  - Drug of choice for reversal of acute opioid intoxication.
  - Onset is rapid, duration is also rapid (on the order of minutes)
    - Must be re-dosed every 2 to 3 minutes.
  - If given to a patient with chronic opioid use or with pain, naloxone will reverse all analgesia and precipitate withdrawal.
  - Dosing:
    - IV 0.04mg Q3-5 minutes (Intoxication)
    - IV/IM/SubQ 0.4 -2mg Q2-3 minutes (Overdose)
      - May dose lower and repeat as needed to minimize precipitating withdrawal.
    - IN 4mg/0.1mL Q2-3 minutes [Narcan]
    - May require drip to combat long-acting agonists.
  - Naloxone has poor bioavailability as a PO medication. Therefore, in order to discourage abuse, many combination PO full agonist medications have naloxone incorporated into the formulation. In its PO form naloxone will not precipitate withdrawal, but should the drug be abused via injection or snorting the naloxone will exert its antagonist properties and prevent overdose.

***Managing patients with a history of opioid use or opioid use disorder:***

- **Breakthrough dosing** - for patients that are using opioids chronically to manage their pain, their chronic pain plans typically include a dose for breakthrough pain. This dose is typically 10-20% of their daily opioid dose. Therefore, if the patient takes 15mg of ER morphine BID, for 30mg total each day, then the patient likely has a dose of 3-6mg of IR morphine for their breakthrough pain. This breakthrough dosing is prescribed as needed, typically Q4H PRN. Daily pain plans are modified by the primary provided managing the patient’s pain if the patient requires frequent breakthrough dosing. Breakthrough dosing may be useful in determining what starting dose to give a patient when they present with acute pain and a history of chronic pain, as it may help the EM provider assess for potential tolerance or cross-tolerance while attempting to prescribe and manage acute pain.
- When **converting immediate release (IR) to extended release (ER) (and vice versa)** add the total amount taken in one day of immediate release and divide by number of daily administrations of extended release.
  - Ex: Patient takes IR morphine 5mg Q4H daily
    - Patient takes 30mg of IR morphine total in a day.
    - ER morphine has a Q12H formulation, therefore the patient should take 15mg Q12H of this particular ER morphine to have equivalent analgesic effect to the IR 5mg they were taking Q4H.
- **Switching opioids and Incomplete cross-tolerance:** In chronic opioid use, tolerance to the drug develops, which means that higher doses of the same medication may be required to meet necessary levels of analgesia. However, this tolerance does not always translate across different types of opioids. Therefore, when starting a different opioid, it is necessary to decrease the dose in relation to daily dosing of the home/chronic opioid to avoid overdose or adverse effects.
  - ***Decrease dose of new type of opioid to 50-75% of the dose of the chronic type of opioid and titrate up as necessary.*** You may be more conservative with the amount you decrease by if the patient is not receiving appropriate analgesia from the chronic dose.
- Methadone maintenance and combination of buprenorphine/naloxone (Suboxone) treatments for opioid use disorder do not provide analgesia for the patient. If in acute pain, patients on these treatments would also require supplemental analgesia/opioids if warranted. These medications for OUD should be continued though, based on limited evidence. More work must be done to fully understand the guidelines for managing acute pain in patients receiving OUD treatment.^^[[33]](#footnote-33)^^
- Alford et al. (2006) developed a useful list of considerations for managing acute pain in the patient with OUD, particularly those receiving treatment.^^[[34]](#footnote-34)^^ These considerations may also be useful for patients who use opioids chronically or have SUD/OUD and are not on OUD treatment. The following is a summarized list of the considerations Alford et al. (2006) suggest.
  - **Addiction and pain: considerations for patients with OUD on treatment**
    - Reassure the patient that their history of substance use will not preclude adequate pain management.
    - Continue the patient’s standard dose for their OUD treatment.
    - Verify the patient’s methadone or buprenorphine maintenance doses with the prescribing physician or center.
    - Notify the addiction treatment program or prescribing physician regarding the patient’s admission and discharge from the hospital and confirm the time and amount of last maintenance opioid dose. Also note any medications, such as opioids and benzodiazepines, given to the patient as a part of their care during the hospitalization because they may show up on routine urine drug screening.
  - **Acute pain with chronic opioid use or addiction: considerations for pain management**
    - Use shared decision-making strategies to decrease patient anxiety regarding the pain.
    - As needed for the severity of the pain, consider and use conventional analgesics, including opioids.
    - Consider that these patients may require higher opioid analgesic doses administered at shorter intervals due to tolerance, cross-tolerance, or heightened pain sensitivity.
    - Write for continuous pain control with frequent reassessment, as opposed to as needed pain control.
    - Avoid using mixed agonist and antagonist opioids.
  - **Acute pain and methadone: considerations for pain management with ongoing methadone use**
    - Continue methadone maintenance dose.
    - Choose short-acting opioid analgesics for ongoing pain control.
  - **Acute pain and buprenorphine use: select one of four options.**
    - Continue buprenorphine maintenance therapy and utilize immediate release opioid analgesics (for pain of short duration only).
    - Divide buprenorphine dose to administer it every 6–8 hours.
    - Discontinue buprenorphine and use opioid analgesics. Convert back to buprenorphine therapy when the patient no longer requires opioid analgesics to manage pain.
    - Hospitalized patient: discontinue buprenorphine therapy and switch to low-dose methadone. Prescribe immediate-release opioid analgesics to treat pain. Have naloxone available as needed while receiving opioids. At discharge, convert inpatient methadone back to buprenorphine.

***Additional Pain Management Medications***

- **Ketamine (subdissociative doses)**
  - noncompetitive N-methyl-D-aspartate (NMDA) receptor antagonist in the brain and spinal cord as well as a partial mu receptor agonist.
  - Administration/Pharmacology:
    - Dosing: IV 0.1–0.3 mg/kg x1 dose over 10-15 minutes OR 0.15 mg/kg/hr as a continuous infusion
      - Note that this is lower than procedural dosing and anesthetic dosing.
    - Onset of action: 30-45 seconds
    - Duration of action: 30-45 minutes
    - Common indications: Analgesic adjunct in cases of severe pain in the unstable patient, intractable pain, neuropathic pain, or opioid-tolerant and opioid-induced hyperalgesia.
    - Common side/adverse effects: sedation, agitation, reemergence phenomenon, nausea, vomiting, dizziness, nystagmus, catecholamine surge (decreased risk of side effects when infused over 10-15min).
    - Patients with chronic obstructive pulmonary disease, cystic fibrosis, and neuromuscular disorders affecting respiratory effort are particularly susceptible to opioid related respiratory suppression, therefore ketamine may be preferred in these patients.
- **Lidocaine**
  - Administration/Pharmacology:
    - Dosing: 1-1.5 mg/kg IV if dosed by weight, some studies in the Emergency Department dose in standard doses of 50 or 100mg.^^[[35]](#footnote-35)^^
      - Maximum Dose: 200mg
    - Duration of action: Varies, onset is within minutes.
    - Common indications: still experimental and under investigation. Commonly used in pain clinics for patients with chronic pain or neuropathic pain. Being explored in the Emergency Department for use in patients with severe pain, chronic opioid use/contraindications to other medications, or failure of other medications.^^[[36]](#footnote-36)^^
    - Common side/adverse effects: Systemic toxicity (neuro and cardiovascular symptoms * see below).
- **Neuromodulating pain medications**
  - The following medications are useful in the emergency department but do not always have immediate onset. Therefore, in acute pain, they represent adjuncts to start or continue for prolonged pain. The dosing, indications, and pharmacology are out of the scope of this reading and seminar.
  - Why do anticonvulsants work in managing pain?
    - Chronic or neuropathic pain = disruption or overactivity of nociceptive input to the STT… just like epilepsy (but in the brain)!
    - Anticonvulsants target sodium channels (much like local anesthetics, i.e. lidocaine) and increase GABA while blocking presynaptic calcium channels (involved in signal transduction of pain).
  - Anticonvulsants used to manage pain:
    - Gabapentin
    - Carbamazepine/Oxcarbazepine
    - Lamotrigine
    - Topiramate
  - Why do antidepressants work in managing pain?
    - This mechanism is a bit more unclear.
    - We do know that MAOIs and SSRIs are ineffective against pain.
      - This signals that pain relief is not merely a byproduct of treating depression (though there is a known biological and psychological connection between pain and depression).
    - TCAs and SNRIs are effective, both of which target norepinephrine reuptake.
      - Many believe that chronic or neuropathic pain may therefore possess some component of sympathetic derangement (though clonidine is only somewhat effective in certain patients…).
  - Antidepressants used to manage pain:
    - Amitriptyline
    - Nortriptyline
    - Venlafaxine
    - Duloxetine
- **Other medications useful in treating pain (or medications your patients may be on for pain)**
  - Prednisone - anti-inflammatory properties can help with inflammatory pain.
  - Baclofen - GABA-B agonist that is useful for spasticity and more specifically muscle spasm.
  - Cyclobenzaprine (Flexeril) - 5-HT2 receptor antagonist that works centrally to decrease muscle spasm.
  - Clonidine - alpha-2 agonist.
  - Dopamine Receptor (D1-R, D2-R) Antagonists - metoclopramide, prochlorperazine, chlorpromazine, haloperidol, and droperidol.
  - Dexmedetomidine - alpha-2 agonist (used in ICUs for mechanically ventilated patients).

***Interventional Pain Management***

- *The EMRA Pain Management Guide* starts its chapter on interventional pain management with this salient quote: *“Ultrasound-guided nerve blocks (UGNBs) are a vital part of the multimodal approach to the treatment of acute pain in the emergency department. The classic mono-modal approach, which has been the cornerstone of emergency medicine training, has been shown to produce inadvertent acute effects (hypotension, apnea, delirium, etc.) and chronic conditions (opioid addiction) as downstream consequences.”*
- While developing a pain plan, always ask yourself: would an interventional pain strategy be beneficial for this patient and enhance your multimodal pain plan?
- Using ultrasound guidance, a nerve block is a procedural pain management strategy that can be highly effective and highly targeted.
- Like any procedure, it is important to practice and prepare.
- **Preparation for a nerve block includes**:
  - Recognizing that a patient may be a candidate.
  - Assessing for contraindications such as coagulopathy, anesthesia allergies, or history of neurologic deficiency.
  - Consenting the patient.
  - Placing the patient on the monitor and getting peripheral access (in case any resuscitative measures must be taken during or because of the intervention).
  - Document a pre- and post-procedural neurologic exam.
  - Gather **materials** including an ultrasound, your anesthetic, appropriate needle and syringe, and potential assistant if injecting a large volume (two-person block recommended). Know where the intra-lipid is in case the patient begins to show signs of toxicity.
  - See Life in the Fast Lane’s CIMPLE-D setup for more information on setting up blocks: <https://litfl.com/regional-nerve-block/>
- **Dosing of local anesthetic**:^^[[37]](#footnote-37)^^
  - The Highland EM Ultrasound blog has a wonderful online guide for reference of specific dosing recommendations: <http://highlandultrasound.com/med-guide>.
    - The Pain Pocket Card provided with the seminar materials also has a guide on dosing.
  - Dosing should be considered based on the duration of analgesia required (is it for a short procedure, a time-limited surgery, or a longer duration while in the Emergency Department and on the wards?) and the site of the block. Esters are rapidly metabolized and therefore preferred for short procedures.
  - Epinephrine added to the local anesthetic slows the absorption prolongs the effect of the block while also increasing its intensity, therefore patients can receive higher maximum doses/volumes of local anesthetics with epinephrine.
  - Other patient considerations for dosing:
    - *Age:* pediatric patients metabolize the block slower and are prone to toxicity at higher doses. Nerves in elderly patients have been shown to be more sensitive to local anesthetic, which in addition to decreased metabolic/organ function also warrants decreasing the standard dose.
    - *Kidney function*: patients with impaired kidney function have decreased clearance of local anesthetics and therefore may require smaller doses or closer monitoring.
    - *Heart failure or hepatic dysfunction:* these patients likely have hypo-dynamic circulation or associate poor kidney function which puts them at risk for toxicity. Many may be able to tolerate a normal dose, but it is often recommended to start with smaller doses.
    - *Pregnancy:* pregnant patients have increased sensitivity and increased cardiotoxicity with bupivacaine and ropivacaine due to progesterone in their circulation. Due to the changes in hemodynamics, clearance, and sensitivity, large volume blocks should be avoided in the first trimester and all other standard doses should be decreased.
    - ***These considerations may contribute to increased risk of local anesthetic systemic toxicity.*
- **Following the nerve block:**
  - Document the amount of anesthesia used, location of the block, time of administration, and any potential complications associated with the block.
  - Repeat a neurologic exam and make note.
  - Assess the patient’s pain immediately after and a short time after to check for adequate analgesia/effect.
- **Types of nerve blocks and indications:**
  - For a list of nerve blocks and their indications you may choose to refer to:
    - ACEP Sonoguide blog post: <https://www.acep.org/sonoguide/nerve_block.html#NBoverviewref1>
    - WikEM: <https://wikem.org/wiki/Nerve_and_regional_blocks_(main)>
    - Reichman, E. F. "Chapter 126. Regional nerve blocks (regional anesthesia)." Emergency medicine procedures, 2e. New York, NY: McGraw-Hill (2013): 815-6.
    - 5 Minute Sono - Nerve Blocks: <https://www.coreultrasound.com/5ms/>
    - See the Pain Pocket Card provided with the seminar materials for a diagram on the types of nerve blocks and indications.
- **Local Anesthetic Systemic Toxicity (LAST)^^[[38]](#footnote-38)^^**:
  - Occurs relatively infrequently -- estimated 1.8 incidences per 1000 nerve blocks.
  - The majority of cases occur within the first hour following the block, with approx 20% happening 1-12 hours after the block.
  - What causes LAST? “The general clinical presentation of LAST reflects the relevant molecular and organ targets. Local anaesthetic pharmacotoxicity results from a combination of adverse effects on ionotropic and metabotropic cell signalling as well as energy transduction.”
  - CNS signs (may be subtle or absent):
    - Excitatory symptoms - agitation, confusion, seizures
    - Depressive symptoms - mental slowing, decreased consciousness, apnea, coma
    - Non-specific - perioral paresthesia, tinnitus, diplopia, metallic taste
  - CV signs (may be isolated; typically progressive):
    - Hypertension and tachydysrhythmias
    - Hypotension
    - Bradycardia
    - Conduction block
    - Ventricular arrhythmias
    - Asystole
  - Management:
    - Immediately stop the local anesthetic agent.
    - Secure the airway as needed; avoid hypercarbia (increases delivery to brain) and hyperventilation (decreases cardiac output).
    - Benzodiazepines for seizures.
    - Cardiopulmonary resuscitation as warranted.
    - **Lipid Emulsion therapy is the only way to remove the anesthetic and truly treat the toxicity!**
      - Dosing: An initial bolus of 1.5 ml/kg of 20% lipid over 2–3 min (approximately 100 ml in a 70 kg adult patient) is recommended, followed by an infusion of 0.25 ml/kg/minuntil 10 min after stability is achieved (average total dose approximately 2.8 ml/kg).

***Discharge from the Emergency Department with Pain Management***

- In 2017, the American Academy of Emergency Medicine released a white paper on the management of pain in the Emergency Department. This document includes basic guidelines and recommendations for prescribing while the patient is in the Department and upon discharge from the Department, as supported by the literature.^^[[39]](#footnote-39)^^ This is a great reference for those seeking further guidance on pain management in the Emergency Department, especially as it pertains to planning for a patient’s discharge.
- Some patients may not be ill enough to require admission, but they still may be in pain at the time of discharge. In these scenarios, emergency providers will have to counsel the patient on expectations for pain management in the emergency department and at home. It may be necessary to send the patient home with prescriptions for analgesics.
- **Immediate Release is a must**. Because providers in the emergency department are limited in the amount of patient education they can provide, it is difficult and potentially dangerous to prescribe extended-release medications, especially when prescribing outpatient opioids. As the discharge medications are supposed to serve as a bridge to outpatient management, 2-3 days of immediate-release analgesics are preferable.^^[[40]](#footnote-40)^^
- **Steps to prescribing pain management at the time of discharge**:^^[[41]](#footnote-41)^^
  - Have a conversation with the patient to engage in shared-decision making -- What does the patient feel comfortable going home with? Do they understand risks/benefits of various medications?
  - If it is determined that opioid analgesics are the most suitable discharge medication, then the prescribing physician should opt for immediate-release opioids for a 2-3 day course (the shorter the course, the better). View home regimens of analgesia as a bridge to follow up.
    - Consider a multi-modal approach in these scenarios.
      - Continue to consider the analgesic ceilings of the medications you prescribe.
    - Consider the patient’s comorbidities - obstructive sleep apnea and pulmonary disease put patients at higher risk for respiratory depression.
    - If prescribing a combination pill, be sure not to exceed the daily maximum dose for the non-opioid analgesics (ex: APAP if prescribing oxycodone-acetaminophen).
    - Review side effects and risks with the patient, in addition to special instructions.
      - Many patients continue to drive after starting a short course of opioids after discharge.^^[[42]](#footnote-42)^^
    - Counsel patients on safe storage and possible disposal of opioids.
    - Ensure the patient has follow-up by the end of the prescribed course so that the pain may be addressed, if persistent, by the primary physician.
  - Consider any additional medications that may alleviate the patient’s particular pain. For instance, if the pain is due to gastritis, the patient may be able to receive a GI cocktail and leave with a PPI.
  - Be clear and specific about pain management instructions - include **dosing**, **special instructions**, and **clear timing** (“every 8 hours” preferred to “three times a day”) when writing out pain care instructions.
  - Include non-pharmacologic options in the discharge instructions, such as “RICE” (rest, ice, compression, elevation) for MSK pain and activity levels (especially in back pain -- bed rest is not recommended!).
- For diagnosis specific discharge considerations, EMRA’s *Pain Management Guide* (2020) serves as a useful resource.

1. Hogans BB, Barreveld A, eds. *Pain Care Essentials*. Oxford: Oxford University Press; 2020. [↑](#footnote-ref-1)
2. Chisewski, David H. *EMRA Pain Management Guide*, EMRA Publications, USA, 2020. [↑](#footnote-ref-2)
3. Ducharme J. Acute Pain Management. In: Tintinalli JE, Stapczynski J, Ma O, Yealy DM, Meckler GD, Cline DM. eds. *Tintinalli’s Emergency Medicine: A Comprehensive Study Guide*, 8e. McGraw-Hill; 2016. [↑](#footnote-ref-3)
4. Rosenzweig S, Lopez BL, Oettinger G. Acute Pain Management. In: Wolfson AB, Hendey GW, Ling LJ, Rosen CL, Schaider JJ, Sharieff GQ. eds. *Harwood-Nuss' Clinical Practice of Emergency Medicine,* 7e. Lippincott Williams & Wilkins; 2012. [↑](#footnote-ref-4)
5. James R. Miner, Knox H. Todd, “Ch 75 - Pain Management in the Emergency Department”, pages 1009-1014, in *Practical Management of Pain,* 2014. Editor(s): Honorio T. Benzon, James P. Rathmell, Christopher L. Wu, Dennis C. Turk, Charles E. Argoff, Robert W. Hurley. [↑](#footnote-ref-5)
6. Catherine A. Marco, William Kanitz, and Matthew Jolly, "Pain scores among emergency department (ED) patients: comparison by ED diagnosis," *The Journal of Emergency Medicine* 44.1 (2013): 46-52. [↑](#footnote-ref-6)
7. Baker, Edward James, and Geraldine Ann Lee. "A retrospective observational study examining the effect of thoracic epidural and patient controlled analgesia on short-term outcomes in blunt thoracic trauma injuries." *Medicine* 95.2 (2016). [↑](#footnote-ref-7)
8. Wilson JE, Pendleton JM. Oligoanalgesia in the emergency department. *Am J Emerg Med.* 1989;7:620-623. [↑](#footnote-ref-8)
9. Timothy Rupp and Kathleen A. Delaney, "Inadequate analgesia in emergency medicine," *Annals of emergency medicine* 43.4 (2004): 494-503. [↑](#footnote-ref-9)
10. Sarah Axeen, Seth A. Seabury, and Michael Menchine, "Emergency department contribution to the prescription opioid epidemic," *Annals of emergency medicine* 71.6 (2018): 659-667. [↑](#footnote-ref-10)
11. Todd, Knox H. "A review of current and emerging approaches to pain management in the emergency department." *Pain and therapy* 6.2 (2017): 193-202. [↑](#footnote-ref-11)
12. Vanderah, Todd W. "Pathophysiology of pain." *Medical Clinics* 91.1 (2007): 1-12. [↑](#footnote-ref-12)
13. Fisher, Casey J., Yaksh, Tony L., Bruno, Kelly, Eddinger, Kelly A. “Chapter 1 - Basic Science of Pain”

    Editor(s): Sanjong Pangarkar, Quynh G. Pham, Blessen C. Eapen, *Pain Care Essentials and Innovations*,

    Elsevier, 2021, Pages 1-14. [↑](#footnote-ref-13)
14. Drewes, Asbjørn M., et al. "Gastrointestinal pain." Drewes, Asbjørn M., et al. "Gastrointestinal pain." *Nature Reviews Disease Primers* 6.1 (2020): 1-16. 6.1 (2020): 1-16. [↑](#footnote-ref-14)
15. Cohen, Victor, et al. "Development of an opioid reduction protocol in an emergency department." *American Journal of Health-System Pharmacy* 72.23 (2015): 2080-2086. [↑](#footnote-ref-15)
16. Finnerup, Nanna B. "Nonnarcotic methods of pain management." *New England Journal of Medicine* 380.25 (2019): 2440-2448. [↑](#footnote-ref-16)
17. Rainsford, K. D. *Ibuprofen: pharmacology, therapeutics and side effects*. Springer Science & Business Media, 2013. [↑](#footnote-ref-17)
18. Becker, Daniel E. "Pain management: Part 1: Managing acute and postoperative dental pain." *Anesthesia progress* 57.2 (2010): 67-79. [↑](#footnote-ref-18)
19. Brutzkus JC, Shahrokhi M, Varacallo M. Naproxen. [Updated 2020 May 24]. In: StatPearls [Internet]. Treasure Island (FL): StatPearls Publishing; 2020 Jan-. Available from: https://www.ncbi.nlm.nih.gov/books/NBK525965/ [↑](#footnote-ref-19)
20. Lucas, Sylvia. "The pharmacology of indomethacin." *Headache: The Journal of Head and Face Pain* 56.2 (2016): 436-446. [↑](#footnote-ref-20)
21. Buckley, Micaela M-T., and Rex N. Brogden. "Ketorolac." *Drugs* 39.1 (1990): 86-109. [↑](#footnote-ref-21)
22. Soleyman-Zomalan, Emil, et al. "Patterns of Ketorolac dosing by emergency physicians." World Journal of Emergency Medicine 8.1 (2017): 43. [↑](#footnote-ref-22)
23. *EMRA Pain Management Guide*: Ch. 17 - Pharmacology of Pain, Jenny Koehl, PharmD, BCPS; Massachusetts General Hospital. [↑](#footnote-ref-23)
24. Valentino, Rita J., and Nora D. Volkow. "Untangling the complexity of opioid receptor function." *Neuropsychopharmacology* 43.13 (2018): 2514-2520. [↑](#footnote-ref-24)
25. Terrie, Yvette C. "An overview of opioids." *Pharmacy Times* 77.6 (2011): 36. [↑](#footnote-ref-25)
26. Powell, Marissa Z., Scott W. Mueller, and Paul M. Reynolds. "Assessment of opioid cross-reactivity and provider perceptions in hospitalized patients with reported opioid allergies." *Annals of Pharmacotherapy* 53.11 (2019): 1117-1123. [↑](#footnote-ref-26)
27. Wightman R, Perrone J, Portelli I, et al. Likeability and Abuse Liability of Commonly Prescribed Opioids. J Med Toxicol. September 2012. doi: 10.1007/s12181-012-0263-x [↑](#footnote-ref-27)
28. Hoppe JA, Nelson LS, Perrone J, Weiner SG, Prescribing Opioids Safely in the Emergency Department (POSED) Study Investigators. Opioid Prescribing in a Cross Section of US Emergency Departments. Ann Emerg Med. 2015;66(3):253–259. [↑](#footnote-ref-28)
29. Figure designed by author M.S. with guidance from: <https://palliative.stanford.edu/opioid-conversion/equivalency-table/> and <https://www.aafp.org/dam/AAFP/documents/patient_care/pain_management/conversion-table.pdf> [↑](#footnote-ref-29)
30. Mazer-Amirshahi, M., S. Motov, and L.S. Nelson, Hydromorphone use for acute pain: Misconceptions, controversies, and risks. *J Opioid Manag*, 2018. 14(1): p. 61-71. [↑](#footnote-ref-30)
31. Scott, Lesley J., and Caroline M. Perry. "Tramadol." *Drugs* 60.1 (2000): 139-176. [↑](#footnote-ref-31)
32. Juurlink, D. “Tramadont.” EMCrit blog, May 7 2018. https://emcrit.org/toxhound/tramadont/ [↑](#footnote-ref-32)
33. Veazie, Stephanie, et al. "Managing Acute Pain in Patients Taking Medication for Opioid Use Disorder: a Rapid Review." Journal of General Internal Medicine (2020): 1-9. [↑](#footnote-ref-33)
34. Alford, Daniel P., Peggy Compton, and Jeffrey H. Samet. "Acute pain management for patients receiving maintenance methadone or buprenorphine therapy." Annals of internal medicine 144.2 (2006): 127-134. [↑](#footnote-ref-34)
35. e Silva, Lucas Oliveira J., et al. "Safety and efficacy of intravenous lidocaine for pain management in the emergency department: a systematic review." Annals of emergency medicine 72.2 (2018): 135-144. [↑](#footnote-ref-35)
36. Clattenburg, Eben J., et al. "Intravenous lidocaine provides similar analgesia to intravenous morphine for undifferentiated severe pain in the emergency department: a pilot, unblinded randomized controlled trial." Pain Medicine 20.4 (2019): 834-839. [↑](#footnote-ref-36)
37. Rosenberg, Per H., Bernadette Th Veering, and William F. Urmey. "Maximum recommended doses of local anesthetics: a multifactorial concept." *Regional Anesthesia & Pain Medicine* 29.6 (2004): 564-575. [↑](#footnote-ref-37)
38. Macfarlane, A. J. R., et al. "Updates in our understanding of local anaesthetic systemic toxicity: a narrative review." *Anaesthesia* 76 (2021): 27-39. [↑](#footnote-ref-38)
39. Motov, Sergey, R. Strayer, and B. Hayes. "AAEM White Paper on Acute Pain Management in the Emergency Department." *American Academy of Emergency Medicine* (2017). [↑](#footnote-ref-39)
40. Hoppe JA, Nelson LS, Perrone J, Weiner SG, Prescribing Opioids Safely in the Emergency Department (POSED) Study Investigators. Opioid Prescribing in a Cross Section of US Emergency Departments. Ann Emerg Med. 2015;66(3):253–259. [↑](#footnote-ref-40)
41. Strayer RJ, Motov SM, Nelson LS. Something for pain: Responsible opioid use in emergency medicine. *Am J Emerg Med*. 2017 Feb;35(2):337-341. [↑](#footnote-ref-41)
42. Mcintosh, Scott E., and Stephen Leffler. "Pain management after discharge from the ED." *The American journal of emergency medicine* 22.2 (2004): 98-100. [↑](#footnote-ref-42)
