# Supplementary material for: Demystifying Acute Pain Management in the Emergency Department: A Case-Based Approach
Source: MedEdPORTAL. 2023 Aug 22;19:11339. doi: 10.15766/mep_2374-8265.11339 (PMC10442463; doi:10.15766/mep_2374-8265.11339)
Supplement: Supplementary file 1 — Chalk Talk Board Maps.docxPatient Case.docxPresession Knowledge Assessment.docxPostsession Knowledge Assessment.docxPocket Card.pdfFacilitator Guide.docxFacilitator Notes and Prereading.docxAnnotated Knowledge Assessment.docx [file mep_2374-8265.11339-s001.zip › E. Pocket Card.pdf]

MANAGEMENT of ACUTE PAIN

Characterize the patient's pain.

"LMNOPQRST" History
Location, Medical changes (assoc. sxs), Nausea, Onset, Provocation/Palliation, Quality, Radiation, Severity, Timing

Examine the patient using trauma informed care and validate the pain experience.

Mentally "classify" the patient's pain.

What type of pain is the patient experiencing given the history?
Nociceptive, Inflammatory, Neuropathic, Visceral

Where in the pain signaling pathway will your analgesics target?
Transmission, Transduction, Perception, Modulation

Develop a pain plan.

1. What will your **multi-modal** approach include?
2. If your initial analgesic method fails, what will your **second and third line** attempts include?
3. Has your pain plan accounted for the patient's **entire stay** in the department through discharge/admission?

APAP/NSAIDs

Medication	Dose	Frequency	Onset	Peds Dose
Acetaminophen (APAP)	PO: 325-650mg<br>IV: 1g; PR: 650mg	Q4-6H<br>Max:3-4g/d	PO: 1 hour<br>IV: 5-10min	PO 15 mg/kg, Q4-6H<br>Max: 90mg/kg/day
Ibuprofen	PO: 400-600mg	Q6-8H<br>Max: 3.2g/d	1-2 hours	PO 10 mg/kg, Q4-6H<br>Max: 40mg/kg/d or 2.4g/d
Naproxen	PO: 250-500mg	Q8-12H<br>Max: 1.5g/d	30-60 min	PO 5 mg/kg, Q12H<br>Max: 1g/d
Ketorolac	IV/IM: 10-15mg	Q8-12H<br>Max: 120mg/d	30-40 min	IV 0.5-1 mg/kg, Q6H<br>Max: 15-30mg Q6Hx5d
Diclofenac	PO: 50mg	Q8H<br>Max: 150mg/d	1-2 hours	2 to 3 mg/kg/d divided Q6-12H; limited data
Indomethicin	PO: 25-50mg	Q6-12<br>Max: 200mg/d	1-1.5 hours	>2yr: PO 1-2 mg/kg;Q6-12H<br>Max: 4mg/kg/d or 200mg/d
Meloxicam	PO: 7.5-15mg	QD<br>Max: 15mg/d	Hours	----

APAP Side Effects:
Nausea, vomiting, liver toxicity, liver necrosis.
(All primarily at supratherapeutic doses.)

NSAID Side Effects:
GI upset, GI ulcers/ bleeding, risk of hemorrhage, renal dysfunction, bronchospasm, delayed wound healing.

Analgesic Ceilings:
Consider investigating trials that identify dosing for max analgesic effect.
Ex: Ibuprofen = 400mg.

OPIOIDS**

Medication	Starting Dose	Onset	Frequency	Peds Dose (>6mo)
Morphine (IR)	PO: 7.5-15mg	30-60 min	Q3-6 hours	PO: 0.3mg/kg,Q4H
Morphine (IR)	IV: 0.05-0.1mg/kg<br>Typically 2-10mg	5-10 min	Q2-4 hours<br>2mg q5-10min on initial titration	IV: 0.1mg/kg,Q2-4H
Hydromorphone	PO: 1-4mg<br>IV: 0.2-1mg<br>(0.015mg/kg)	PO: 15-30 min<br>IV: 2-5 min	PO: Q4-6H<br>IV: Q2-4H after initial titration	PO: 0.03mg/kg, Q4H<br>IV: 0.01mg/kg, Q3-6H
Oxycodone (IR)	PO: 5-10mg	30-60 min	Q4-6H	PO: 0.1 to 0.2 mg/kg, Q4-6H (<50kg)
Hydrocodone combo w/APAP	PO: 7.5mg/325mg	30 min	Q4-6H<br>*Max: APAP/d	PO: 0.1 to 0.2 mg/kg, Q4-6H (<50kg)
Fentanyl	IV: 0.35-1mcg/kg<br>Max 250mcg	Seconds to minutes	Q30-60min<br>*Titrate	IV: 0.35-1mcg/kg, Q1-2H (<50kg)

Side Effects of Opioids:
Sedation, constipation, respiratory depression, hepatic accumulation (*fentanyl*), hypotension from histamine release (*most prominent with morphine*), muscle/chest wall rigidity (*fentanyl -- primarily when large doses given rapidly*), renal accumulation especially in elderly patients and those with renal disease (*all but fentanyl; hydromorphone metabolites easier to remove with dialysis*).

Equianalgesia

Medication	PO	IV	PO MME
Morphine	30mg	10mg	1
Hydromorphone	7.5mg	1.5mg	4
Oxycodone	20mg	---	1.5
Hydrocodone	30mg	---	1
Fentanyl	---	0.1mg	---

Opioid Overdose: Naloxone

IV, IM, SubQ: Initial: 0.4 to 2 mg, repeat every 2 to 3 minutes
* A lower initial dose (**0.04mg Q2-3min**) could be considered to avoid acute withdrawal symptoms, titrate to support respiration.

IN: 4 mg (contents of 1 nasal spray as a single dose in 1 nostril)
* Repeat doses every 2 to 3 minutes

After 10mg administered, consider other etiologies.
May need to consider IV infusion if overdose with sustained release or long-acting opioids.

Pain Plan Approach

First-line Analgesic - route and dose

Improvement?
Yes → *When will you re-dose? Adjuncts?*
Somewhat/Partially/Not Sufficient ↓

Second-line Analgesic - route and dose

Improvement?
Yes → *When will you re-dose? Adjuncts?*
Somewhat/Partially/Not Sufficient ↓

Third-line Analgesic - route and dose

Improvement?
Yes → *When will you re-dose? Adjuncts?*
Somewhat/Partially/Not Sufficient ↓

Interventional analgesia?

Acute Pain Management Pocket card.
Compiled and designed by Morgan Sehdev. Image: Author Owned, Image by Morgan Sehdev, retrieved from and designed on: www.canva.com on 03/12/2021. Free Stock Media and Creative Commons License associated: www.canva.com.
Reviewed by **Eli Phillips, PharmD, BCPS and Holly Reed, PharmD**. Updated 03/2021.

MANAGEMENT of ACUTE PAIN

NON-OPIOIDS

Medication	Dose	Frequency	Onset	Mechanism	Notes
Ketamine (analgesic)	IV: 0.1-0.4mg/kg<br>IM/IN: 0.5-1mg/kg	Give IV bolus over 15min, +/- infusion	secs to mins	NMDA receptor antagonist	Useful in opioid tolerant or unstable patients.<br>**Side Effects**: nystagmus, agitation, dizziness, catecholamine release, re-emergence reaction
Ketamine (procedural)	IV: 1-2mg/kg	Initial bolus, with 0.5-1mg/kg Q10min	secs to mins	NMDA receptor antagonist	
Lidocaine	IV: 1.5mg/kg over 10 min	Initial bolus, may consider infusion	minutes	Sodium Channel Blockers	**Side Effects**: EKG changes, dizziness, parasthesias
Dexmedetomidine	IV: 0.5 to 1.0 μg/kg	Typically given as an infusion	minutes	Central alpha-2-adrenergic receptor agonist	ICU sedative/analgesic, research on ED use ongoing
Gabapentin	PO: 300mg<br>(max 3600mg/d)	Dose QHS to TID, **needs titrated**	days to weeks	Calcium Channel Blocker (↓glutamate)	good for neuropathic pain, less bioavailable at higher doses, increase euphoria with opioids
Duloxetine	PO: 30mg	Daily	Varies	SNRI	May release endogenous opioids.<br>**Side Effects**: dry mouth, sedation, nausea, caution with serotoninergics
Amitriptyline	PO: 10-25mg<br>(max 150mg/d)	QHS<br>Titrate dose	Varies	TCA	**Side Effects**: sedation, nausea, constipation, dry mouth, caution in elderly + patients w/ CV disease
Baclofen	PO: 5-10mg<br>(max 80mg/d)	TID as needed, short-term use	2-3 hours	GABA agonist	Useful for spasticity, use cautiously<br>**Side Effects:** nausea, confusion, dizziness, risk of withdrawal
Sumatriptan	PO: 50 to 100 mg<br>IN: 20mg	x1, may repeat once after ≥2 hrs	1-2 hours	Selective serotonin receptor agonist	Abortive treatment for migraine **Side Effects:** fatigue, chest pain, prolonged QTc, sore throat. **AVOID** in patients with ischemic heart disease (will ↑ angina).

LOCAL ANESTHETICS

Sodium Channel Blockers

Medication	Max Dose	Onset	Concentration	Max Dose	Duration	Class
Lidocaine (with Epi)	7mg/kg	Rapid	1% (10mg/mL)	500mg/50cc (7mg/kg)	1.5-3 hours	Ester
Lidocaine (without Epi)	4.5mg/kg	Rapid	1% (10mg/mL)	300mg/30cc (5mg/kg)	1-2 hours	Ester
Chloroprocaine (with Epi)	14mg/kg	Rapid	2% (20mg/mL)	1000mg/50cc (15mg/kg)	0.5-1 hour	Amide
Chloroprocaine (without Epi)	11mg/kg	Rapid	2% (20mg/mL)	800mg/40cc (10mg/kg)	0.5-1 hour	Amide
Ropivacaine (without Epi)	3mg/kg	Medium	0.5% (5mg/mL)	225mg/45cc (3mg/kg)	3-6 hours	Ester
Bupivacaine (with Epi)	3mg/kg	Slow	0.5% (5mg/mL)	225mg/45cc (3mg/kg)	5-8 hours	Ester
Bupivacaine (without Epi)	2.5mg/kg	Slow	0.5% (5mg/mL)	175mg/35cc (2.5mg/kg)	3-6 hours	Ester

DISCHARGE PLANNING

1. Engage in shared decision making.
2. Take a multi-modal approach.
3. Immediate release is always preferred (always PO).
4. 2-3 day supply to last until follow up.
5. Consider patient co-morbidities and the maximum daily doses.
6. Review side effects carefully with the patient.
7. Counsel patient on safe use, storage, and disposal.
8. Write clear, specific, hour-based dosing instructions and return precautions.

SAMPLE DISCHARGE INSTRUCTIONS

For pain at home, you can take Acetaminophen 1000mg up to 4 times a day, with 6 hours in between each dose. Do not take more than 4000mg of Acetaminophen in one 24 hour period (one day). You can also take Ibuprofen 800mg up to 4 times a day, with 6 hours in between eacd dose. Do not take more than 3200mg of Ibuprofen in one 24 hour period (one day). For best results, you can stagger each medication by three hours for more pain control.

Example schedule:

- 6am - 1000mg of Acetaminophen
- 9am - 800mg of Ibuprofen
- 12pm - 1000mg of Acetaminophen
- 3pm - 800mg of Ibuprofen
- 6pm - 1000mg of Acetaminophen
- 9pm - 800mg of Ibuprofen

Continued as needed.

**Considerations in Chronic Pain, Chronic Opioid Use, or Opioid Use Disorder

- **Incomplete Cross-Tolerance:** In opioid tolerant patients, tolerance to one opioid does translate into complete tolerance of a different opioid. Therefore, you need to reduce dose by 25-50%.

Calculate dose accounting for Incomplete Cross-Tolerance:

1. Add up total daily dose of given opioid.
2. Convert daily dose to Morphine Milligram Equivalents (MME).
3. Determine equivalent daily dose of new opioid by dividing the calculated MMEs of current opioid by new opioid's conversion factor. Reduce this amount by 25-50% and then divide into appropriate intervals.

- **Opioid Tolerance:** Those on chronic opioids may require higher doses of opioids acutely; therefore, continue to titrate the recommended doses above to analgesic effect while monitoring respiratory status.
- **Always treat acute pain!** Continue OUD treatment (suboxone, methadone, etc.) as able, but provide adequate analgesia. Attempt non-opioid options first, consider higher doses/increased frequency.
- For patients with chronic pain, **continue home regimen**.
- **Breakthrough pain** opioid doses are typically 10-15% of the daily total opioid in immediate release form. *Example:* patient's total daily morphine is 40mg, breakthrough dose is 4-6mg morphine immediate release.

Acute Pain Management Pocket card.
Compiled and designed by Morgan Sehdev. Image: Author Owned, Image by Morgan Sehdev, retrieved from and designed on: www.canva.com on 03/12/2021. Free Stock Media and Creative Commons License associated: www.canva.com.
Reviewed by **Eli Phillips, PharmD, BCPS and Holly Reed, PharmD**. Updated 03/2023.
