# Supplementary material for: Demystifying Acute Pain Management in the Emergency Department: A Case-Based Approach
Source: MedEdPORTAL. 2023 Aug 22;19:11339. doi: 10.15766/mep_2374-8265.11339 (PMC10442463; doi:10.15766/mep_2374-8265.11339)
Supplement: Supplementary file 1 — Chalk Talk Board Maps.docxPatient Case.docxPresession Knowledge Assessment.docxPostsession Knowledge Assessment.docxPocket Card.pdfFacilitator Guide.docxFacilitator Notes and Prereading.docxAnnotated Knowledge Assessment.docx [file mep_2374-8265.11339-s001.zip › F. Facilitator Guide.docx]

**Emergency Medicine: Acute Pain Management**

**Seminar for Senior Medical Students and Junior Trainees**

**Seminar Overview**

**Duration:** 2.5 hours

**Seminar Goals:**

There exists an identified need within undergraduate and graduate medical education to enhance education regarding pain and pain management. Pain care can be particularly challenging in settings like the Emergency Department, where orders are expediently required and often various comorbidities, hemodynamic instabilities, psychosocial dimensions, or past medical history factors may also be at play. Confidently managing and understanding pain is a basic skill necessary to succeed in the emergency department early on in residency and therefore a skill that senior medical students and trainees should begin to practice in advanced electives, sub-internships, and early residency. The overarching goal of this seminar will be to refamiliarize senior medical students or junior residents with the basic pathophysiology of pain, options for treating pain including medication dosing, and considerations for developing a pain plan while accounting for the patient’s history and hospital course.

**Seminar Objectives:**

By the end of the seminar, participating learners will be able to:

1. Summarize the differences between the four different types of pain.
2. Efficiently assess a patient’s pain on presentation to the Emergency Department.
3. Appropriately dose (including administration route, dose, frequency) three opioid and three non-opioid pain medications.
4. Design an acute pain management plan, from initial evaluation in the Emergency Department to discharge planning and prescribing, for a case-based scenario.
5. Identify three differences that need to be made in an acute pain plan for a patient with a history of substance use disorder as compared to a patient without a history of substance use disorder.

**Seminar Format/Schedule:**

- *Before the Session:*
  - Learners will take 5 to 10 minutes to complete the pre-test knowledge assessment
  - Before the session, allow time for the facilitator to draw the blank board map on the white board.
  - Ask learners to bring a phone, tablet, or computer for activity; alternatively the session could be conducted in a space with computers. If computer access is limited, you may choose to bring print papers/resources for the “Interactive Activity.”
- *During the Session:*
  - **0:00-0:05 - Introduction**
  - **0:05-0:20 - Assessing Pain -- didactic portion using Chalk Talk Board Map #1**
  - **0:20-0:35 - Assessing Pain: Patient Case Part I**
  - **0:35-0:40 - Introduction to Pain Management**
  - **0:40-0:55 - Interactive Activity**
  - **0:55-1:00 - Break**
  - **1:00-1:35 - Managing Pain -- didactic portion using Chalk Talk Board Map #2**
  - **1:35-1:50 - Managing Pain: Patient Case Part II**
  - **1:50-2:00 - Final Considerations, Special Cases, Discharge**
  - **2:00-2:10 - Wrap Up and Questions**
  - **2:10-2:30 - Post-test Knowledge Assessment and Review**
- *After the Session*:
  - Distribute pocket cards which contain a smaller version of the chart we built together in addition to some other useful information for on-the-go in the ED

**Materials:**

- Board maps
  - If the facilitator has to conduct this session virtually, the facilitator may use the “Chalk Talk” board maps provided in Appendix A. If the facilitator has access to a tablet or may annotate easily, they may use the blank Board Maps in Appendix A and annotate Maps themselves according to the Empty/Completed Board Maps provided.
- 4 colors of dry erase markers
  - Alternatively, “chalk talk” can be drawn on paper for smaller groups or projected/converted to PowerPoint
- Pre-/post-test print outs
- Pocket cards

**How to Use the Facilitator Guide:**

This Facilitator Guide is built to lead anyone through the presentation of this two-hour introduction to managing acute pain in the Emergency Department. The Facilitator Guide provides suggested timing, goals for each section, notes, discussion points/questions, and resources to reference.

- Timing is written in **BLUE**.
- Key discussion points/questions for learners are written in ***RED***.
- Suggested use of Board Maps for the chalk talk in the seminar are **Bolded/Highlighted**.

Further resources can be found in **“A Primer on Pain: Facilitator Notes and Pre-Reading” (**Appendix G). The primer contains notes and further reading suggestions for potential facilitators who would like to review pain-related material and contextualize any aspects of the seminar. This appendix is provided so that facilitators may find the seminar material more accessible. If a topic is covered in the appendix in depth, there is less elaboration in this facilitator guide. Any questions related to content in this guide may be addressed in the primer (Appendix G), as together the resources are meant to be additive as opposed to repetitive.

**Facilitator Session Guide and Script:**

**0:00-0:05 - Introduction**

1. Welcome learners and contextualize the seminar. You may choose to insert a personal anecdote from caring for a patient in pain and potentially finding it difficult to manage or lacking familiarity with pain management options. This anecdote may or may not be the reason you chose to facilitate this seminar for your learners!
2. Review overarching goals and objectives for the seminar with learners.
3. Provide context as to why this is important -- **approximately 78% of Emergency Department visits carry some form of a pain-related complaint** [you may choose to fill this in on **Board #1** when sharing this or repeat and fill in later].

**0:05-0:20 - Assessing Pain [Board Map #1]**

*Prior to the session, it is important to have drawn the* ***Empty Board Map #1*** *on the whiteboard/chalkboard you will use so as to save time. Together with the learners, the facilitator will fill in the board according to the provided* ***Board Maps****.* Any information not found in this guide can be found on the **Completed Board Maps** and elaborated in the “**Primer on Pain:** **Facilitator Notes and Pre-Reading**.”

For the next **15-minutes**, the facilitator will help learners review the physiology of pain, the types of pain patients can experience, and how to assess pain. The following points are *suggested considerations for discussion* as it pertains to filling out the **first whiteboard (Board Map #1)**. This discussion is best if learner-led. As the facilitator, prompt the learners and encourage their participation to fill out each section of the board together. It is advised that the facilitator serve as the scribe for learner comments.

1. ***Physiology of pain***
   1. Assess learners' recollections of the **spinothalamic tract** and **how it is organized**, as this part is descriptive you may opt to have a learner join you at the board to fill in/draw the first, second, and third order neurons.
   2. Briefly review of the types of nerve fibers and the stimuli they process.
   3. Briefly describe the four processes involved in pain signaling: transduction, transmission, perception, and modulation. ***Highlight that this is important to know as analgesics will target different steps in the pain signaling pathway**.
2. ***Types of pain***
   1. Review Nociceptive, Inflammatory, Neuropathic, and Visceral Pain.
   2. Discuss how pain may be one or more of these types of pain, discuss the etiology of these types of pain, and briefly review the differences.
   3. Targeting the type of pain based on the most appropriate analgesic for that pain helps in crafting a multi-modal pain plan.
3. ***Pain Assessment***
   1. Introduce the “drive by”–walk past the room to see how patient comfort level may change by your presence/arrival. Are they writhing in pain even without their provider present? Are they asleep or quietly/comfortably looking at their phone?
   2. Review the importance of thorough history. Include discussion of the value of the social history and past medical history, as this information can alter pain plans.
   3. Review a pain-centric physical exam while validating the pain and practicing trauma informed care.
   4. Review that labs and imaging may be necessary, but it’s most advisable to follow guidelines based on your differential diagnosis (outside the scope of talk).
   5. Review Numeric Rating Scale (NRS) as one scale to measure pain and how a change of 2 or more points after analgesia is a clinically significant change.
   6. Review how assessing change in functional status really helps gauge the effect of the pain on the patient → i.e., does pain limit work? limit walking?
4. ***Why is pain assessment important?***
   1. Review ED visits and % of pain diagnoses, review oligoanalgesia (patient/provider/systems related causes).
   2. Review widespread impacts of pain on other organ systems, including but not limited to vital sign changes to look out for.
   3. Review benefits of patient comfort through analgesia when required and the need to provide empathetic care.^^[[1]](#footnote-1)^^

**0:20-0:35 - Assessing Pain: Patient Case Part I**

Using the case write up below, the facilitator will walk learners through an introduction to the case that will be used to anchor the seminar’s discussion and apply the concepts introduced in the more didactic portion in the previous part of the seminar. This **15-minute** section of the seminar is included to allow learners to ***practice assessing*** ***the patient’s pain***. Goals for this section of the seminar include:

- Eliciting the “LMNOPQRST” of the pain
- Interpreting the pertinent physical exam findings
- Identifying the type of pain being experienced
- Constructing the differential diagnosis
- Assessing for any biases that may play into the next portion of the case discussion

***Suggested approaches for this section*:**

- ***In person***: facilitator can give the group the Chief Complaint (CC) and have the group ask questions of the facilitator as if the facilitator was the patient responding (using case write up below).
- ***In person***: facilitator selects one learner to be the “clinician” and role play as though the facilitator (or selected co-facilitator) is the patient.
- ***In person***: if the group is small enough you may choose to use [insert appendix].
- ***Virtual***: facilitator presents the CC, then invites each learner to ask a question they would pose to the patient (either out loud or in the platform’s “chat” function) with the facilitator providing the responses.
- ***In person or virtual*:** option to record the information that the learners acquire on a whiteboard (if there is space available) or while annotating on the screen. You may want to choose a scribe as facilitating and scribing would increase the time required for this section. Alternatively, the facilitator may ask learners to take their own notes as they find useful.

***Suggested questions to pose to learners and possible points of discussion***:

1. CC: 41F with a history of IBD and chronic low back pain presents with fever and right sided abdominal pain. ***What would you like to know next?***
   1. Guide learners through HPI (below) including: location, movement/migration, nausea (good for visceral vs somatic pain), onset, provocation/palliation, quality, radiation, severity, timing if they don’t inquire on their own.
   2. Pain assessment scales -- Numeric Rating Scale (NRS) among others.^^[[2]](#footnote-2)^^
      1. When there are language barriers/cross-cultural differences, the visual analog scale (VAS) is the preferred pain assessment tool.
   3. **Note**: HPI is intentionally vague to allow for a broader differential, facilitator may editorialize as needed.
2. ***What more do you want to know about the patient’s chronic low back pain?***
   1. Guide learners through the patient's pain regimen at home.
   2. This will be an introduction to managing patients with home pain regimens, questions about implications will be deferred to Part II of the patient case.
3. ***What physical exam maneuvers would you perform once you’ve completed your history?***
   1. Have learners name the physical exam maneuvers they would perform and verbally give the findings. You may discuss the implications of findings as you see fit.
4. ***Focusing particularly on the vital signs and abdominal exam, what do you make of the findings and how does this inform your understanding of Ms. C’s presentation?***
   1. **Vital Signs:** Metoprolol or other medications may mask the typical vital signs we expect to see in pain (tachycardia, hypertension) and pain may mask hypotension of SIRS picture. Be careful when relying on vital signs!
   2. **Abdominal Exam:** Tenderness concerning for a lower abdominal or renal etiology. May consider doing special maneuvers to elucidate whether GYN, GI, or GU.
5. ***Using the framework for the four types of pain (nociceptive, neuropathic, inflammatory, visceral) and considering the history you’ve acquired, what type of pain do you believe Ms. C is experiencing?***
   1. Most likely an inflammatory and/or visceral pain—the pain is acute, there’s a peristaltic nature to diffuse and poorly localized pain, the patient is febrile, pain is dull and crampy.
6. ***When triaging a patient, why is it important to at least quickly consider what type of pain a patient is describing? How might knowing the various types of pain change your management or understanding of a patient’s pain?***
   1. Comment on how the type of pain can modify the approach you’ll take to management—for example neuropathic pain is less likely to respond to NSAIDs.
7. ***What are the top five diagnoses on your differential and why? Which of these are you most concerned about?***
   1. Answers will vary depending on how little or how much information the facilitator gives to the group. This answer is not critical to moving the discussion forward but helps ground the learners and may inform their decisions later.
   2. Example List: Renal Colic or Pyelonephritis, appendicitis, Cystitis, TOA/ovarian torsion, perhaps abdominal vascular pathology.
8. ***Ms. C is a middle-aged woman and a Spanish-speaking immigrant, how do you believe this may impact her care? What biases do you think may impact her care and how might they do so?***
   1. There is no one right answer to this question and this could be an entire seminar unto itself. However, to have a holistic understanding of pain care in the emergency department, we felt it was necessary to at least introduce this concept.
   2. Race has been identified to play a role in differential prescribing. While nothing is consistent across institutions, some papers have reported that while there is no racial difference in those who receive pain medication there is a provider tendency to preferentially prescribe opioids to white patients. One paper states that when cases were matched, white patients received opioids 10% more often.^^[[3]](#footnote-3)^^ One meta-analysis found that in acute pain patients, black patients received analgesia less often than their white counterparts (OR 0.60); the same was true for Hispanic patients (OR 0.75).^^[[4]](#footnote-4)^^
   3. Gender of both the patient and the provider may also play a role in prescribing analgesia in the Emergency Department. Historically, women patients often receive less analgesia than men. In some presentations, such as abdominal pain, women have been found to receive analgesia less often and if they do it is less often opioid analgesia.^^[[5]](#footnote-5)^^ Physician gender may play a role in these disparities as well.^^[[6]](#footnote-6)^^

***For Facilitator Reference -- In-session Patient Case, Part I:***

**CC:** 41F with fever and abdominal pain

**HPI:** Ms. C is a 41yo Spanish-speaking woman with a history of IBD who presents with several hours of right sided abdominal and side pain -- patient is seen with an interpreter. [*For the purposes of this case, we will assume that the interpreter was available for the duration of the visit and that the potential language barrier is not impairing your ability to assess the patient or understand fully the patient history/exam. For sessions that may be longer, the facilitator may alternatively include this as a barrier and discuss care options -- see Footnote.^^[[7]](#footnote-7)^^*]

The patient was in her usual state of health when she noticed a “twinge” in her right side while sitting at her desk this afternoon. This sensation comes and goes in waves and has progressively worsened from a “twinge” to a “sharp pain”, 7-9/10 in severity and radiating to the right groin. But, she is also complaining of diffuse lower abdominal pain that is also “dull and crampy.” Nothing seems to make the pain better and she cannot find a comfortable position. She is most concerned because the intermittent pain has become more constant over the past two hours. She endorses associated nausea without vomiting. The patient thinks she may have a fever, and does become diaphoretic during the waves of pain. Ms. C has not noticed any blood in her urine, though it has been darker over the past couple of days which she attributes to decreased water intake due to a stressful deadline at work.

At home, she takes twice daily extended-release morphine 15mg for chronic low back pain -- she has taken this morning’s dose (it is now late afternoon/early evening). States that her pain has been well controlled on this medication, which was started on in Guatemala before immigrating. 400mg of Ibuprofen at home helped minimally with her worsening pain, which is why she chose to come in as the pain was unbearable at home.

Otherwise, the patient has a history of diabetes treated with metformin and hypertension treated with metoprolol. She was diagnosed with IBD as a teenager and is now in remission; only takes a daily multivitamin (no steroids or immune modulators). No prior surgeries. Non-smoker with no prior drug use. Sexually active with her husband. Last menstrual period was 3 weeks ago. Review of systems is otherwise notable for fatigue and some recent weight loss.

**Exam:** General: **Uncomfortable, in moderate distress, moving around in bed**. Awake, alert, and oriented.

VS: Temp 38.2 C, HR 90, BP 105/85, RR: 14

HEENT: Oropharynx clear. EOMI, PERRLA, no scleral icterus.

CV: Regular rate and rhythm. No murmurs, rubs, or gallops.

Pulm: Clear to auscultation bilaterally.

Abd: **Bowel sounds present but decreased.** Nondistended. **Mild guarding,** **tender to deep palpation in the RLQ>LLQ. CVA tenderness on the right. No rigidity. Negative Murphy’s sign. Equivocal psoas sign.**

Neuro: CN 2-12 intact. Motor function is normal with muscle strength 5/5 throughout. Sensation is intact bilaterally. Reflexes 2+ bilaterally.

**0:35-0:40 - Introduction to Pain Management**

Now, after having established the types of pain and how to assess pain, the seminar will move towards learning how to **manage** the pain. The introduction to this section would be an optimal time to gauge learners’ comfort and familiarity with pharmacologic pain management options (a real time “educational needs assessment”) to tailor the level of difficulty and depth of ensuing discussion.

**0:40-0:55 - Interactive Activity**

The goal of this activity is to have learners research and become the **“15 Minute Expert”** on one of the medications that will later be discussed as a large group. ***Depending on the size and format of your seminar, the facilitator may need to assign multiple learners to an individual medication or assign an individual learner to multiple medications. If working in groups, the facilitator may ask learners to find a physical space to work together in the in-person session or establish breakout rooms in the virtual session.***

Learners will have 15-minutes to complete the independent, interactive activity in which they search for papers/textbook entries about their assigned medication and prepare to briefly present this medication to the group. **Learners should either be directed to view the diagram drawn on the empty version of Board #2 (using Board Map #2) or to write down the information they are to search for and later present if Board #2 is not yet mapped onto the whiteboard/chalkboard**. Informing learners of the pertinent information they later need to provide prevents them from searching for extraneous information!

**The information learners present will help fill in Board Map #2, if the facilitator only has access to one whiteboard/chalkboard for an in person session this would be the optimal time to take a picture of the completed board #1 (to distribute later to learners), erase board #1, and put the Empty Board Map #2 on the whiteboard/chalkboard while the learners complete their independent searches.**

*Learners will be asked to learn about the following medications:*

1. Ibuprofen
2. Naproxen
3. Ketorolac
4. Diclofenac
5. Acetaminophen
6. Morphine
7. Hydromorphone
8. Oxycodone
9. Fentanyl
10. Ketamine
11. Lidocaine

*Learners will be asked to report back on the following about each medication:*

1. **Possible routes of administration** (NOTE: emphasis on PO, IV, and note if available in topical -- no other dosing will be discussed)
2. **Dosing for an adult** (NOTE: again, only for PO/IV and immediate release formulations; do not look up extended-release formulation or pediatrics dosing)
3. **Frequency of dosing**
4. **Onset of effect/duration of effect**
5. **Maximum daily dose**
6. **Common indications in the Emergency Department**
7. **Common side effects**
8. **Special considerations or notes** **about the medication** (anything interesting the learner finds in the fifteen minutes)

*Directing learners to the following (or other facilitator identified) resources may be useful for the activity:*

1. Hogans BB, Barreveld A, eds. *Pain Care Essentials*. Oxford: Oxford University Press; 2020.
2. Cisewski DH, Motov S, eds. *EMRA Pain Management Guide*. USA: EMRA Publications; 2020.
3. Ducharme J. Acute Pain Management. In: Tintinalli JE, Stapczynski J, Ma O, Yealy DM, Meckler GD, Cline DM. eds. *Tintinalli’s Emergency Medicine: A Comprehensive Study Guide*, 8e. McGraw-Hill; 2016.
4. UpToDate or other databases and search by medication.

**0:55-1:00 - BREAK**

**1:00-1:35 - Pain Management [Board Map #2]**

Overarching goal for the next **15-minutes** is to review the selected pain management options. The following are *suggested questions and considerations for discussion* as it pertains to filling out the second whiteboard to complete **Board Map #2**.

1. ***Review as a group the basic mechanism of action of the NSAIDs, APAP, opioids, and ketamine.***
   1. *If the facilitator has access to two boards or is doing this virtually, this would be the time to reference the neural pathway on* ***Board #1*** *and review *briefly* where on the spinothalamic tract these medications are thought to act while annotating on the diagram.*
2. ***Learners will then report out on their research and help the facilitator construct of the chart of analgesics. Supplement learners’ responses as necessary, some possible points:***
   1. With dosing, it may be helpful to consider the lowest dose you can give in pill form and use that as a benchmark to determine if your dose may need to be higher/lower.
   2. For IV opioids, in opioid naive patients especially, you can start on the low end of the recommended range. Reassess pain every 5-10min (or write order for this) to redose opioid (as analgesic onset occurs within 10 min) and titrate for relief while trying to avoid side effects. **Ex**: 2mg IV morphine can be titrated by 2–5-mg increments every 5–10 min for a change of ≥2 NRS points. On average, this titration goes up to a dose of 10mg Q3-4H.
   3. Hydromorphone at equianalgesic levels creates more respiratory depression and euphoria.^^[[8]](#footnote-8)^^ Though a lower “dose”, still a lot of opioid to give repeatedly and at the same frequency as morphine. May be useful for patients with renal failure (though fentanyl may be even more appropriate)^^[[9]](#footnote-9)^^ or patients requiring elevated doses of morphine (i.e., opioid tolerant, cancer patients, etc.).
   4. **Morphine Equivalents^^[[10]](#footnote-10)^^:** 30mg PO Morphine = 10mg IV Morphine
      1. 7.5mg PO Hydromorphone = 1.5mg IV Hydromorphone
      2. 10mg IV Morphine = 1.5mg IV Hydromorphone = 100mcg (0.1mg) IV Fentanyl
   5. IV Lidocaine - a randomized trial has shown that in acute severe pain an IV Lidocaine bolus over 10 min followed by an infusion was equivalent to morphine in reducing pain and required less breakthrough/repeated morphine doses with less side effects.^^[[11]](#footnote-11)^^
      1. Still not a standard practice, so Lidocaine is identified on chart/in this seminar to keep you aware of the developing prospects in the pain literature.
   6. A majority of the time the medication you select will be dependent on the patient’s renal/hepatic function, age, blood pressure, and severity/etiology of pain.
3. ***Comment on the need to understand these basic properties of each medication, possible discussion points include:***
   1. Indications guide how the clinician may piece together multimodal treatment
      1. **Pain regimens have been studied for specific chief complaints.** It may be beneficial to review the literature and evidence for various presentations. Some of the indications are included on **Board Map #2** and should be discussed. ***Pieces to highlight****:* evidence for treating back pain points to using NSAIDs, not opioids, with adjuncts such as neuromodulators, trigger point injections, and topical medications. (*Reviewing back pain here allows students to consider the evidence-based guidelines that may aid in their construction of a pain plan. This comment also helps students complete the knowledge assessment back pain question.*) Migraines require much different analgesics.
      2. Nausea and GI complaints may also require different medications. *(Last two CC beyond the scope of the talk, but the facilitator may choose to extend the session and supplement this information.)*
      3. Pain severity and the **“analgesic ladder”** - for mild to moderate pain work up to stronger pain medications (i.e. start with NSAIDs); for severe pain may choose to reverse the ladder using a **“step down”** approach and start with stronger analgesics (i.e. opioids/interventional pain procedures). There are other constructions such as the pain pyramid and platform that may be useful to review or mention.
   2. Route of administration is important! Is the patient NPO? Can you use the topical form of that NSAID in the elderly patient with back pain?
   3. Onset of action, duration of action, and recommended frequency help determine when the clinician may reassess the patient’s pain or the instructions they may give to nursing for subsequent “PRN” or as needed doses.
   4. Special considerations are important to review, especially comorbidities, as the clinician may need to adjust their original pain plan to avoid complications such as a hypotension or further damage to kidneys.
4. ***Having covered the medications and filled in each respective “row” on the analgesics chart, contextualize the importance of knowing these medications.***
   1. Review the WHO’s analgesic ladder^^[[12]](#footnote-12)^^ as mentioned above or consider the “Analgesic Platform.”^^[[13]](#footnote-13)^^
   2. **Differences between NSAIDs** - review classes of NSAIDs (propionic acid/acetic acid/oxicam derivatives); you may choose to contextualize by also listing indomethacin (acetic acid derivative) or meloxicam (oxicam) as NSAIDs often used in osteoarthritis.
   3. **Differences between opioids** - review that opioids can be synthetic, semi-synthetic, or natural. There are classes called Phenanthrenes, Phenylheptylamines, and Phenylpiperidines. If a patient has a sensitivity to opioids, they likely are sensitive to opioids in the same class. As such, you could carefully trial a different class if warranted for severe pain.

**1:35-1:50 - Managing Pain: Patient Case Part II**

Returning to the case of Ms. C, the facilitator will now lead a discussion on the pain plan for Ms. C. The facilitator may want to remind the group of the chief complaint and the 5 possible differential diagnoses that the group came up with in Part I.

*CC: 41F with a history of IBD presents with worsening, non-specific lower abdominal and flank pain, concerning for __, ___, ___, ___, and ___. [Insert the learners’ differential diagnoses here.]*

This section is intended to be primarily driven by the learners’ discussion and collaboration. **Goal: Create a Pain Plan for Ms. C.** *Suggested discussion points to facilitate the discussion include:*

1. ***Given what we discussed previously about Ms. C, how would you approach developing a pain plan for her?***
   1. **Multimodal!** Multimodal approaches=using multiple analgesics that target different stages in pain signaling or types of pain; using multiple administration forms such as interventional, topical, and systemic; using combinations of classes such as NSAIDs and opioids. Exciting framework developed by Dr. Motov ([www.painfree-ed.com](http://www.painfree-ed.com)) and Maimonides faculty: channel enzyme receptor-targeted analgesia (CERTA).^^[[14]](#footnote-14)^^ Consider each analgesic’s target and address as many targets as you can in your pain plan.
   2. Patients in acute pain require pain relief! If someone’s in pain, you won’t get them high. Don’t fear opioids, be comfortable with them, and attempt to be judicious but fair regarding their use (based on pain levels and not biases).
   3. **Consider the patient’s chronic use of morphine for back pain— there are implications for tolerance and incomplete cross tolerance.**
      1. **Tolerance:** requiring higher dose of chronic opioid for analgesic effect
      2. **Incomplete Cross Tolerance:** when starting a new or different opioid in a patient with chronic opioid use, it is necessary to decrease the dose in relation to daily dosing of the home/chronic opioid to avoid overdose or adverse effects. Dose the new opioid at 50-75% of chronic dose.
2. ***What medication(s) and at what dose/frequency would you prescribe?***
   1. Analgesia in abdominal pain, contrary to prior beliefs, does not increase the risk of diagnosis error or the risk of diagnostic failure.^^[[15]](#footnote-15)^^
   2. **Analgesic Ceiling:** define and provide example: Ketorolac 10, 15, 30mg studied in the ED and equally effective.^^[[16]](#footnote-16)^^
   3. RTCs have shown that non-opioids are equally effective as opioids in renal colic (assumed working diagnosis for Ms. C right now).^^[[17]](#footnote-17)^^
   4. **First line treatment option:** Ketorolac 10-15mg (IV) **or** Morphine 2-10 mg IV (may increase given home morphine); could consider combination of the two drugs x2 doses each.^^[[18]](#footnote-18)^^ (**Or** titrate hydromorphone 0.2-1mg for patients who don’t respond to or can’t receive prior two options)
3. *If the learners do not bring up the idea of reassessing:* ***You find that the workup you’ve ordered is still being processed, but Ms. C has received your initial analgesic. In the meantime, when would you go back and check on Ms. C? Explain what you would be looking for during reassessment****.*
   1. Most medications have onset of analgesic effect within 15-30 minutes, therefore you should consider revisiting Ms. C then. Consider a “drive by.” With the patient, re-check NRS (or pain scale you applied) to evaluate for clinically significant decrease. Does the patient need more? Do they need another agent? Are there new or worsening symptoms?
4. ***What would be a second line medication that you would consider? Again, at what dose/frequency?***
   1. **Second line:** Could consider redosing any of the above or move on to the next options.
   2. **Second line:** Acetaminophen 1000mg (PO vs. IV) Q6H **or** Lidocaine 1-1.5mg/kg IV (adjunct to morphine?^^[[19]](#footnote-19)^^) **or** Ketamine 0.1-0.3mg/kg IV over 15 minutes.^^[[20]](#footnote-20)^^ The last two given at titrated intervals as needed. Fentanyl 25-50mcg IV could be considered for patients in severe pain refractory to NSAIDs and morphine.
5. ***Are there any interventional pain procedures you may consider?***
   1. You *could* consider interventional procedures. Not too much evidence here, but always good to ask the question when making a pain plan. Some studies have shown that TAP blocks *can* be effective in management of pain associated with appendicitis.^^[[21]](#footnote-21)^^
6. Say the patient had been *afebrile with no vital sign abnormalities* and your work-up comes back positive for renal colic with stone measuring 4mm*.* ***You decide to discharge Ms. C home with follow up in 2-4 days. How would you approach your discharge planning for her? What pain régimen will you discharge her on?***
   1. A ureteral calculus measuring 6 mm or more and/or situated above the mid-ureter, or residual pain at discharge may predict a high likelihood of urological intervention, other possible predictors include signs of infection or impaired renal function -- in which case you may choose to admit. If she is febrile and with possible vital sign abnormalities (as she is in case and not this hypothetical), you may work up further and admit. Otherwise, if it’s uncomplicated renal colic, she is safe to discharge. Roughly 20% of patients require intervention after discharge.^^[[22]](#footnote-22)^^
   2. Likely discharge plan with 2-5 days of NSAIDs (diclofenac, ibuprofen, naproxen) and possible adjuncts:
      1. Trial of tamsulosin 0.2–0.4 mg once daily for four weeks may help passage of stone.
      2. Diclofenac 50mg PO TID or Ibuprofen 600mg PO Q6H or Naproxen 250mg PO Q8H for 3-5 days.
      3. APAP to alternate with NSAID, if necessary, 500mg PO Q6H for 3-5 days.
      4. Morphine sulfate immediate release 15mg PO Q4-6H -- in a patient with pre-existing requirements could utilize breakthrough pain dose as needed for 2-3 days until able to get follow up visit, or you may consider an increased dose for breakthrough due to tolerance. Use caution with chronic pain plans and avoid altering as you are able.
   3. Follow up with either PCP or Urology. Include hydration recommendations.
   4. Review specifics of discharge planning using **Board #2**.
7. ***Comment on the fact that a “Pain Plan” extends from the first analgesic given to the discharge or admission orders -- this helps make sure that the patient’s pain is well controlled and decreases the number of times you must start from scratch and consider what you’ve given and what you’ll give next when inevitably the pain returns.***
8. **Special consideration*:*** ***If Ms. C had a history of substance use disorder, would your management plan change? If so, how?***
   1. Screen for substance use: AUDIT-C (alcohol) or NIDA-1 Quick Screen. (Doesn’t take a lot of time!)^^[[23]](#footnote-23)^^
   2. While considerations should not be largely different in patients with SUD (treating pain is paramount regardless of SUD), it is important to pause and consider which medications you will prescribe at discharge, which medications are more pro-euphoric, and what supports exist for your patient as an inpatient or outpatient.
   3. In SUD and particularly OUD, trial non-opioids as able and if opioids are necessary, monitor the side effects. These patients may have hyperalgesia from chronic opioids or increased tolerance, these may be patients for whom you’d want to consider including analgesic doses of ketamine or IV lidocaine in the pain plan.
   4. Patients with a history of substance use disorder are concerned about withdrawal symptoms. Continue the patient’s methadone or buprenorphine and monitor with withdrawal scores.^^[[24]](#footnote-24)^^
   5. Non-opioid dependence (ex: alcohol use disorder) typically does not confer higher analgesic needs.^^[[25]](#footnote-25)^^
   6. For patients with alcohol use disorder, you would **not change** your pain plan dramatically. However, you may need to use caution with hepatically-cleared or hepatotoxic analgesics (especially acetaminophen).
9. **Special consideration: *If Ms. C were older than 41, say 70 years old, how would your pain plan change?***
   1. Beers Criteria for Potentially Inappropriate Medication Use in Older Adults -- Recommendations for patients that includes various warnings about NSAIDs, opioids, and TCAs/gabapentinoids.^^[[26]](#footnote-26)^^
   2. Aging comes with changes in pain perception, metabolism, body fat distribution. Palliative Care Network with useful resources.^^[[27]](#footnote-27)^^
   3. Generally, approach mild pain → severe pain following: APAP→NSAIDS (*consider PPI with NSAIDs)→Topicals→Block→Ketamine→Opioids.
   4. *Example of dosing:* Naproxen 200-500 mg BID PRN has less CV risk.^^[[28]](#footnote-28)^^
   5. Consider low-dose titration, or “half and half” method for older adults to decrease opioid needs in severe pain.^^[[29]](#footnote-29)^^ The “half and half” method advises that you split the dose and give one-half followed by the other over a shorter interval during the initial workup.
10. **Special consideration:** ***If Ms. C were instead a pediatric patient in pain, how would you approach her pain plan differently?***
    1. Yackey et al.: 54% of children <4 with fractures received analgesia, of those only 21% were given opioids.^^[[30]](#footnote-30)^^ While this is just one example, pain is often mismanaged in pediatrics (multifactorial problem).
    2. Uncontrolled pain in children can lead to altered perceptions of pain or hyperalgesia later.^^[[31]](#footnote-31)^^
    3. Recognize that there are different pediatric pain scales (FLACC, Wong-Baker).
    4. Consider alternative routes (intranasal, nebulized, rectal) and weight-based dosing for analgesia.
    5. **Useful Resource:** <https://first10em.com/pediatric-pain-management/>
11. ****Optional and if extra time*:** ***How do you convert opioids into morphine equivalents and convert PO to IV?* This is not on a Board Map and would need to be added after looking up morphine equivalents.**

**1:50-2:00 - Special Considerations and Discharge Planning**

In the last **10-minutes**, return to **Board #2** and review some of the discussion points that came up while wrapping up the patient case. Record the suggestions for discharge planning and considerations for patients with chronic opioid use/opioid use disorder (found on **Board Map #2**). For any additional resources the facilitator identifies or provides from the discussion above, feel free to re-share those here, write them down, or offer to send them out after the session.

1. ***Discharge Planning***
   1. Having a plan for discharge is equally important, as patients will leave afraid to be in more pain or re-present quickly when the pain returns.
   2. Clear and detailed instructions are a must! Make yourself a template so you can easily plug instructions into your patient notes/discharge instructions.
2. ***Special Considerations***
   1. Review breakthrough dosing and cross tolerance. Discuss continuing Methadone/Buprenorphine (MAT).

**2:00-2:10 - Wrap Up and Questions**

Leave time for any final comments about the patient case and how it pertains to all the material that was learned, discussed, and reviewed during the seminar. Revisit the seminar’s learning objectives and review how they were met during the session. Create space for remaining learner questions. Offer to share the pain pocket card provided with the seminar materials and offer to forward any citations/pictures of board maps that the learners may find useful.

**2:10-2:30 - Post-test Knowledge Assessment and Review**

The Post-test Knowledge Assessment is provided. Give learners time to fill this out before leaving. After learners complete and return the assessment, the facilitator may offer to spend a couple of minutes reviewing the possible answers to the two cases.

1. Wideman TH, Edwards RR, Walton DM, Martel MO, Hudon A, Seminowicz DA. The Multimodal Assessment Model of Pain: A Novel Framework for Further Integrating the Subjective Pain Experience Within Research and Practice. Clin J Pain. 2019;35(3):212-221. doi:10.1097/AJP.0000000000000670. [↑](#footnote-ref-1)
2. Turk DC, Melzack R. *Handbook of Pain Assessment*. New York: Guilford Press; 2011. [↑](#footnote-ref-2)
3. Mills AM, Shofer FS, Boulis AK, Holena DN, Abbuhl SB. Racial disparity in analgesic treatment for ED patients with abdominal or back pain. Am J Emerg Med. 2011;29(7):752-756. doi:10.1016/j.ajem.2010.02.023 [↑](#footnote-ref-3)
4. Lee P, Le Saux M, Siegel R, et al. Racial and ethnic disparities in the management of acute pain in US emergency departments: Meta-analysis and systematic review. Am J Emerg Med. 2019;37(9):1770-1777. doi:10.1016/j.ajem.2019.06.014 [↑](#footnote-ref-4)
5. Chen EH, Shofer FS, Dean AJ, et al. Gender disparity in analgesic treatment of emergency department patients with acute abdominal pain. Acad Emerg Med. 2008;15(5):414-418. doi:10.1111/j.1553-2712.2008.00100.x [↑](#footnote-ref-5)
6. Safdar B, Heins A, Homel P, et al. Impact of physician and patient gender on pain management in the emergency department--a multicenter study. Pain Med. 2009;10(2):364-372. doi:10.1111/j.1526-4637.2008.00524.x [↑](#footnote-ref-6)
7. Pain management is improved when patients with limited english proficiency interact with providers using interpreters. Several studies demonstrate the benefit of considering barriers to receiving adequate analgesia, **one example:** Jimenez N, Moreno G, Leng M, Buchwald D, Morales LS. Patient-reported quality of pain treatment and use of interpreters in spanish-speaking patients hospitalized for obstetric and gynecological care. J Gen Intern Med. 2012;27(12):1602-1608. doi:10.1007/s11606-012-2154-x

   **One example regarding ED follow up:** Sarver J, Baker DW. Effect of language barriers on follow-up appointments after an emergency department visit. J Gen Intern Med. 2000;15(4):256-264. doi:10.1111/j.1525-1497.2000.06469.x [↑](#footnote-ref-7)
8. Mazer-Amirshahi M, Motov S, Nelson LS. Hydromorphone use for acute pain: Misconceptions, controversies, and risks. J Opioid Manag. 2018;14(1):61-71. doi:10.5055/jom.2018.0430 [↑](#footnote-ref-8)
9. Dean M. Opioids in renal failure and dialysis patients. J Pain Symptom Manage. 2004;28(5):497-504. doi:10.1016/j.jpainsymman.2004.02.02 [↑](#footnote-ref-9)
10. Bhatnagar M, Pruskowski J. Opioid Equivalency. In: StatPearls. Treasure Island (FL): StatPearls Publishing; October 3, 2020. [↑](#footnote-ref-10)
11. Clattenburg EJ, Nguyen A, Yoo T, et al. Intravenous Lidocaine Provides Similar Analgesia to Intravenous Morphine for Undifferentiated Severe Pain in the Emergency Department: A Pilot, Unblinded Randomized Controlled Trial. Pain Med. 2019;20(4):834-839. doi:10.1093/pm/pny03 [↑](#footnote-ref-11)
12. Anekar AA, Cascella M. WHO Analgesic Ladder. In: StatPearls. Treasure Island (FL): StatPearls Publishing; May 17, 2020. [↑](#footnote-ref-12)
13. Leung L. From ladder to platform: a new concept for pain management. J Prim Health Care. 2012;4(3):254-258. [↑](#footnote-ref-13)
14. Cohen V, Motov S, Rockoff B, et al. Development of an opioid reduction protocol in an emergency department. Am J Health Syst Pharm. 2015;72(23):2080-2086. doi:10.2146/ajhp140903 [↑](#footnote-ref-14)
15. Manterola C, Vial M, Moraga J, Astudillo P. Analgesia in patients with acute abdominal pain. Cochrane Database Syst Rev. 2011;(1):CD005660. Published 2011 Jan 19. doi:10.1002/14651858.CD005660.pub3 [↑](#footnote-ref-15)
16. Motov S, Yasavolian M, Likourezos A, et al. Comparison of Intravenous Ketorolac at Three Single-Dose Regimens for Treating Acute Pain in the Emergency Department: A Randomized Controlled Trial. Ann Emerg Med. 2017;70(2):177-184. doi:10.1016/j.annemergmed.2016.10.014 [↑](#footnote-ref-16)
17. Bektas F, Eken C, Karadeniz O, Goksu E, Cubuk M, Cete Y. Intravenous paracetamol or morphine for the treatment of renal colic: a randomized, placebo-controlled trial. Ann Emerg Med. 2009;54(4):568-574. doi:10.1016/j.annemergmed.2009.06.501 [↑](#footnote-ref-17)
18. Safdar B, Degutis LC, Landry K, Vedere SR, Moscovitz HC, D'Onofrio G. Intravenous morphine plus ketorolac is superior to either drug alone for treatment of acute renal colic. Ann Emerg Med. 2006;48(2):173-181.e1. doi:10.1016/j.annemergmed.2006.03.013 [↑](#footnote-ref-18)
19. Motov S, Drapkin J, Butt M, Monfort R, Likourezos A, Marshall J. Pain management of renal colic in the emergency department with intravenous lidocaine. Am J Emerg Med. 2018;36(10):1862-1864. doi:10.1016/j.ajem.2018.07.021 [↑](#footnote-ref-19)
20. Abbasi S, Bidi N, Mahshidfar B, et al. Can low-dose of ketamine reduce the need for morphine in renal colic? A double-blind randomized clinical trial. Am J Emerg Med. 2018;36(3):376-379. doi:10.1016/j.ajem.2017.08.026 [↑](#footnote-ref-20)
21. Mahmoud S, Miraflor E, Martin D, Mantuani D, Luftig J, Nagdev AD. Ultrasound-guided transverse abdominis plane block for ED appendicitis pain control. Am J Emerg Med. 2019;37(4):740-743. doi:10.1016/j.ajem.2019.01.024 [↑](#footnote-ref-21)
22. Papa L, Stiell IG, Wells GA, Ball I, Battram E, Mahoney JE. Predicting intervention in renal colic patients after emergency department evaluation. CJEM. 2005;7(2):78-86. doi:10.1017/s1481803500013026 [↑](#footnote-ref-22)
23. <https://www.masspartnership.com/pdf/CommonlyUsedSUDScreeningInstruments.pdf> [↑](#footnote-ref-23)
24. Veazie S, Mackey K, Peterson K, Bourne D. Managing Acute Pain in Patients Taking Medication for Opioid Use Disorder: a Rapid Review. J Gen Intern Med. 2020;35(Suppl 3):945-953. doi:10.1007/s11606-020-06256-5 [↑](#footnote-ref-24)
25. Quinlan J, Cox F. Acute pain management in patients with drug dependence syndrome. Pain Rep. 2017;2(4):e611. Published 2017 Jul 27. doi:10.1097/PR9.0000000000000611 [↑](#footnote-ref-25)
26. By the 2019 American Geriatrics Society Beers Criteria® Update Expert Panel. American Geriatrics Society 2019 Updated AGS Beers Criteria® for Potentially Inappropriate Medication Use in Older Adults. J Am Geriatr Soc. 2019;67(4):674-694. doi:10.1111/jgs.15767 [↑](#footnote-ref-26)
27. <https://www.mypcnow.org/fast-fact/pain-management-considerations-in-older-adults/> [↑](#footnote-ref-27)
28. Coxib and traditional NSAID Trialists' (CNT) Collaboration, Bhala N, Emberson J, et al. Vascular and upper gastrointestinal effects of non-steroidal anti-inflammatory drugs: meta-analyses of individual participant data from randomised trials. Lancet. 2013;382(9894):769-779. doi:10.1016/S0140-6736(13)60900-9 [↑](#footnote-ref-28)
29. Chang AK, Bijur PE, Davitt M, Gallagher EJ. Randomized clinical trial of an intravenous hydromorphone titration protocol versus usual care for management of acute pain in older emergency department patients. Drugs Aging. 2013;30(9):747-754. doi:10.1007/s40266-013-0103-y [↑](#footnote-ref-29)
30. Yackey KJ, Rominger AH. Are We Adequately Treating Pain in Children Who Present to US Emergency Departments?: Factors That Contribute to Pain Treatment in Pediatric Patients. Pediatr Emerg Care. 2018;34(1):42-46. doi:10.1097/PEC.0000000000000750 [↑](#footnote-ref-30)
31. Krauss BS, Calligaris L, Green SM, Barbi E. Current concepts in management of pain in children in the emergency department. Lancet. 2016;387(10013):83-92. doi:10.1016/S0140-6736(14)61686-X [↑](#footnote-ref-31)
