# Supplementary material for: Demystifying Acute Pain Management in the Emergency Department: A Case-Based Approach
Source: MedEdPORTAL. 2023 Aug 22;19:11339. doi: 10.15766/mep_2374-8265.11339 (PMC10442463; doi:10.15766/mep_2374-8265.11339)
Supplement: Supplementary file 1 — Chalk Talk Board Maps.docxPatient Case.docxPresession Knowledge Assessment.docxPostsession Knowledge Assessment.docxPocket Card.pdfFacilitator Guide.docxFacilitator Notes and Prereading.docxAnnotated Knowledge Assessment.docx [file mep_2374-8265.11339-s001.zip › _Educational Summary Report_11339.pdf]

MedEdPORTAL® The AAMC Journal of Teaching and Learning Resources

Original Publication Open Access

Demystifying Acute Pain Management in the Emergency Department: A Case-Based Approach

Morgan Sehdev, MD*, Jason Lewis, MD, Antje Barreveld, MD

*Corresponding author: msehdev883@gmail.com

Abstract

Introduction: Acute pain is one of the most common complaints that presents to the emergency department. Despite its ubiquity, oligoanalgesia, or the undertreatment of pain, remains a problem in medicine, possibly due to minimal dedicated pain teaching for senior medical students transitioning to residency. **Methods:** We designed a 2.5-hour interactive seminar for senior medical students transitioning into residency. The seminar included a chalk talk and case-based discussion, reviewed pain physiology, revisited pain assessment, and introduced pain management strategies using a novel acute pain plan to organize an analgesic approach from presentation through disposition from the emergency department. The didactic chalk talk was interwoven with a case of acute pain. Seminar materials promoted a near-peer teaching opportunity for future facilitators. Learners completed open-ended pre-/postsession knowledge assessments. **Results:** Data were obtained from 19 fourth-year medical students enrolled in three iterations of a preinternship course at Harvard Medical School. Prior to the seminar, learners scored an average of 23.0 out of 53.0 points ($SD = 9.0$) on the knowledge assessment, which improved to 36.6 out of 53.0 points ($SD = 6.7$) following the seminar (paired t test $p < .001$). Learner satisfaction data revealed a positive response to the seminar: Learners felt more confident managing pain and highly recommended the seminar's continuation in the future. **Discussion:** Initial data from this seminar suggest a need for and benefit of targeted pain education for senior medical students. Seminar materials can easily be adapted for learners in other departments or in early graduate medical education.

Keywords:
Acute Pain, Analgesia, Chalk Talk, Case-Based Learning, Clinical Teaching/Bedside Teaching, Emergency Medicine, Pain Management, Pain Medicine

Educational Objectives

By the end of the seminar, participating learners will be able to:

1. Summarize the differences between the four different types of pain (inflammatory, nociceptive, neuropathic, and visceral).
2. Efficiently assess a patient's pain on presentation to the emergency department.
3. Appropriately dose (including administration route, dose, and frequency) at least three opioid and three nonopioid pain medications.
4. Practice designing a basic acute pain management plan, from initial evaluation in the emergency department to discharge planning and prescribing, for one case-based scenario.
5. Identify three differences that need to be made in an acute pain plan for a patient with a history of substance use disorder as compared to a patient without a history of substance use disorder.

Citation:
Sehdev M, Lewis J, Barreveld A. Demystifying acute pain management in the emergency department: a case-based approach. *MedEdPORTAL*. 2023;19:11339.
https://doi.org/10.15766/mep_2374-8265.11339

Introduction

Acute pain is one of the most common complaints a physician will be asked to manage; however, medical students often do not feel prepared to manage pain or prescribe analgesics in a safe, effective manner as a resident and later as an attending physician.[1] Multiple studies have shown that while most medical schools and residencies provide some form of pain education, pain-specific teaching may be variable, fragmented, and limited.[2-5] Furthermore, deficiencies in pain education may self-perpetuate. A poor foundational understanding of pain as a student or trainee may later hinder teaching on pain as an attending.[6] Today, comprehensive pain education includes recent concerns in pain medicine such as chronic

Copyright © 2023 Sehdev et al. This is an open-access publication distributed under the terms of the Creative Commons Attribution license.

pain and substance use disorder (SUD), but a foundational understanding of basic acute pain and its management is still lacking.[5] The undifferentiated nature and frequency of acute pain presentations in the emergency department (ED) are ideal for discussions on acute pain.[7] Learners in the ED must actively engage with the dueling forces between under- and overtreatment of pain, balancing the risks and benefits associated with oligoanalgesia[8] and opioid overprescription,[9] a balancing act best navigated with a basic understanding of pain pathophysiology and management options. Finally, the triage skills necessary to manage acute pain from presentation through disposition in the ED make for transferable skills that students and later trainees can apply in other rotations or practice settings given the ubiquity of acute pain.

Over the past decade, the number of pain education curricula and resources in *MedEdPORTAL* and elsewhere has surged in response to the overall limited scope of pain management, though most target learners in GME, emphasizing inpatient pain management[10] or opioid use disorder/withdrawal management.[11] Current UME-targeted publications emphasize pain management in palliative care[12] and family medicine.[13] These interventions highlight pain assessment and management specific to critically ill or dying patients and patients managing chronic pain, without focusing on assessment of acute pain in the undifferentiated patient. While *MedEdPORTAL* provides a handful of pain-related emergency medicine (EM) interventions, existing pieces are typically simulations or case-based learning scenarios utilizing a singular diagnosis (e.g., neck pain) as opposed to generalized acute pain.[14,15] To our knowledge, there have been no prior studies or interventions designed for medical students or interns/junior residents that target general acute pain education as it pertains to emergency care or that use a chalk talk approach.

We created and evaluated a 2.5-hour, highly interactive, and unique didactic seminar on acute pain and its management in the ED. The seminar was designed for and implemented with senior medical students.[16] It introduced relevant pain physiology, bias within pain care, and the idea of a presentation-through-disposition pain plan—a concept not featured in pain education interventions yet one particularly relevant for care in the ED and hospital setting at large. We designed the seminar utilizing Kern's frameworks for curriculum design[17] and considering the arc of acute pain care within a fourth-year medical student's zone of proximal development (physiology = "I know," clinical assessment/medication names = "I am learning," prescribing and discharge considerations = "I am soon to be learning"). The material was presented in a chalk talk format to increase in-session discussion and engagement while promoting enhanced postsession retention.[18] We assessed pre- and postseminar knowledge using patient case vignettes and open-ended questions to evaluate learners' knowledge acquisition. The chalk talk format and pain plan framework were developed to promote our primary goal of creating an engaging and accessible educational intervention.

As a secondary goal, we created a tool kit suited for sustainable practice so that the seminar could continue to be broadly implemented. The seminar facilitation materials support a mentored near-peer teaching model that aims to empower student-educators and provide a unique, beneficial experience for near-peer learners.[19] We also designed a facilitation primer as a novel approach to facilitate pain education by decreasing the barrier some educators may face when planning to teach about acute pain.

Methods

This research met exemption criteria outlined by the Harvard Medical School (HMS) Program of Medical Education and Medical Education Institutional Review Board. Informed consent from students was obtained. Students were given the option to abstain from all surveys and assessments.

Curricular Context

HMS recently introduced longitudinal curricular themes, such as those described by Kitzes and colleagues,[20] focusing on common educational gaps identified within the curriculum. Pain and pain management were one of these curricular themes, aiming to incorporate pain teaching throughout the HMS experience in response to the national UME deficit in pain education.[21] As a result, pain-focused seminars became incorporated into several fourth-year preinternship courses. Students were also exposed to 2 days of pain-related material during a second-year course occurring at the transition from preclinical to clinical learning. Currently, the curricular development team is working to interleave more pain-focused teaching throughout the preclinical courses themselves.

Fourth-year medical students at HMS had the opportunity to participate in two different preinternship preparatory courses within the Department of Emergency Medicine at Beth Israel Deaconess Medical Center. The first course was the Emergency Medicine Bootcamp, an optional advanced elective open to students applying to EM residencies or preliminary intern years. The second course, the Emergency Medicine Capstone, was required after applying for residency in EM. Both courses

Copyright © 2023 Sehdev et al. This is an open-access publication distributed under the terms of the Creative Commons Attribution license.

included didactic sessions and simulations focused on preparing students for the transition to intern year. Reflecting on prior iterations of the two courses and HMS's new pain and pain management theme, we recognized a lack of formal teaching on acute pain and its management in the ED throughout the curricula of both courses. Considering the curricular theme's mission as our general needs assessment[17] for the HMS student body, we developed and implemented this seminar in response. Educational objectives for the intervention were informed by medical committee recommendations for acute pain management curricula in UME[22] and EM-specific GME.[4]

Participants and Target Learners

Fourth-year medical students enrolled from February 2021 through April 2022 in the bootcamp and capstone courses described above were required to participate in the acute pain management seminar during prescheduled didactics. Both courses fostered small-group learning within all learning sessions. Subsequently, we designed the seminar to be effective in smaller groups, although the materials could be adapted to meet the needs of larger learning groups. The content of our seminar required basic familiarity with pain signaling and assessment. Our content was most appropriate for a learner with clinical clerkship experience. Though our curriculum was implemented with senior medical students applying in EM, the material would also be suitable and adaptable for medical students or junior residents in other specialties.

Educational Intervention

We developed a highly interactive, 2.5-hour seminar to be incorporated into both courses (note that the material presented in the session could, at facilitator discretion, be spread over multiple shorter sessions). The seminar featured an instructional chalk talk (Appendix A) interwoven with an extended case-based problem (Appendix B). The learners were not required to complete any prereading but were given a 10-minute presession knowledge assessment (Appendix C). To avoid oversurveying the learners, the presession knowledge assessment also served as a targeted needs assessment[17] (any weak points for a particular group of learners given their pooled presession responses could be addressed in greater detail during their seminar). The assessment tools were designed collaboratively by all three authors to implement open-ended, case-based questioning that asked learners to develop a pain plan; the design was informed by best practices in medical education.[23]

The first 40 minutes of the seminar were split between covering the didactic material demonstrated during the first of the two chalk talk board maps (i.e., the depictions of the chalk talk; Appendix A) and covering the first half of the case-based scenario (Appendix B). The chalk talk facilitated a discussion that contextualized pain signaling and types of pain (i.e., nociceptive, inflammatory, neuropathic, visceral) with a particular emphasis on why this physiology could greatly impact management. Additionally, the material highlighted why managing acute pain was important on a physiologic and psychologic level for the patient. This segment concluded by reviewing pain assessment, placing emphasis on pain rating scales and pain-focused physical exams using trauma-informed care. Upon completion of the first chalk talk board map, learners were introduced to a patient case: Ms. C, a 41-year-old, Spanish-speaking woman with a history of inflammatory bowel disease who presented with several hours of right-sided abdominal pain (Appendix B).

The patient case played a supporting role by creating a space to discuss, practice, and apply the material covered during the chalk talk.[24] Specifically, learners practiced collecting a pain-focused history, conducting a pain-focused exam, and identifying the type of pain experienced by the patient. Learners were also encouraged to practice naming special considerations that could globally impact the patient's pain care, as patient features including age,[25] gender,[26] and ethnicity[27] have all been shown to play a role in pain-related health disparities.

The next 80 minutes of the seminar reviewed the material provided on the second chalk talk board map (Appendix A). This segment introduced and reviewed 11 common analgesics. Learners started this portion of the seminar by independently conducting guided research on an assigned analgesic (e.g., morphine). Learners were asked to specifically research analgesic dosing parameters, uses/indications, and side effects/contraindications. Learners returned to the group to report their findings as 15-minute experts on their assigned analgesics. The facilitator then filled the second chalk talk board as directed by the learners, adding detail or clarification as necessary. Upon completion of the analgesics table, the facilitator transitioned to the second half of the patient case. We utilized the 15-minute expert exercise here for several reasons: (1) Learners could practice and troubleshoot navigating suggested on-shift resources, (2) facilitators could introduce an opportunity for active learning utilizing a break-as-action moment,[28] and (3) facilitators could encourage future retention (e.g., learners recalling, "I remember telling the group ______ about this analgesic" or "My colearner taught us ______ about another analgesic"). Those opting not to include the 15-minute expert exercise could consider incorporating additional mini-vignettes and having learners apply the knowledge presented to them after the facilitator has filled in the table.

Copyright © 2023 Sehdev et al. This is an open-access publication distributed under the terms of the Creative Commons Attribution license.

Upon reintroducing the case, the facilitator defined and described the concept of a pain plan (Figure 1). Adapted from chronic and cancer pain management,[29] the acute pain plan asked learners to consider first-, second-, and third-line interventions for pain from presentation through disposition (i.e., home or to the floor/ICU from the ED). We identified a paucity of teaching materials for UME and GME learners emphasizing discharge planning from the ED and therefore wanted to highlight this aspect through the case. Learners practiced developing an acute pain plan for Ms. C from presentation to discharge through guided, open-ended questions. The learners then briefly considered alterations to the pain plan if Ms. C were a pediatric patient, geriatric patient, or patient with SUD. On concluding the case discussion, the facilitator returned to the whiteboard and summarized an approach to discharge planning and constructing patient-centered pain plans.

The session concluded with a 10-minute postsession knowledge assessment (Appendix D). Students were given a pocket card (Appendix E) summarizing the material covered during the seminar and were provided with additional information on acute pain management. Figure 2 offers a visual representation of the workflow through the intervention's various phases.

Facilitator Resources

Author Morgan Sehdev designed the seminar's materials as a part of a peer-to-peer/near-peer initiative within HMS's pain curricular theme. The three iterations of the seminar were delivered by Morgan Sehdev as a fourth-year medical student peer-to-peer facilitator and then as an intern and second-year resident near-peer facilitator, a strategy that promoted

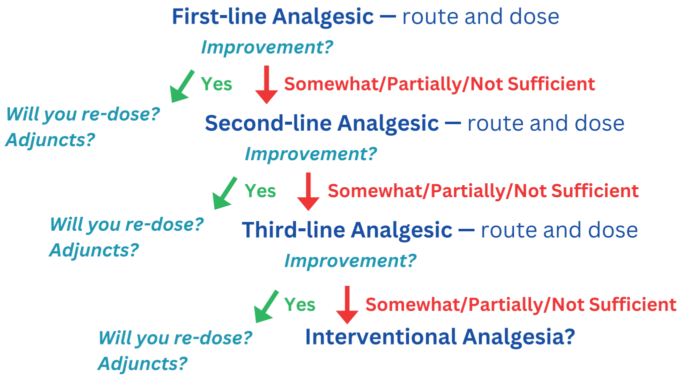

Figure 1. Recommended approach to developing a pain plan. This stepwise progression should be immediately considered after every encounter and mapped out mentally or on a scrap of paper to eliminate the mental burden of asking oneself, "What have I tried? What will I try next?", if notified by the patient or other staff later in one's shift that the initial analgesic did not have much effect for the patient. Depending on one's practice pattern, select a first line using a pain ladder, shelf, or pyramid approach for escalation of analgesia.

learner/facilitator self-efficacy, response efficacy, and cognitive congruence (communicating in the same language as learners). Because the seminar was designed to allow for a near-peer facilitation strategy, we developed a detailed facilitator guide (Appendix F) that included instructions on how to use the session materials and facilitation suggestions. While the facilitator for our seminars was a fourth-year medical student and subsequently a junior resident, the material was designed to support other medical students, residents, or junior attendings seeking to deliver the seminar to either medical student or resident learners. In addition to the guide, we constructed a facilitator primer (Appendix G) summarizing the literature on pain and pain management from authors in both pain medicine and EM. The primer provided facilitators who might feel hesitant to teach on pain with the necessary context to facilitate the seminar. Additional references were listed in the primer should a facilitator desire further reading. Whether used as a starting point or a refresher, the primer served to promote increased pain education.

Assessment and Evaluation

The pre- and postsession knowledge assessments (Appendices C and D) served as the primary subjective and objective assessment of the seminar. Both assessments surveyed learners' confidence in triaging and managing a patient with acute pain on a 5-point Likert scale (1 = *not at all confident,* 5 = *extremely confident* [ready to manage independently as an intern]). Additionally, the assessments provided two patient case vignettes. Two or three open-ended questions accompanied the vignettes to evaluate learners' ability to name the type of pain experienced by a patient; develop a pain plan with a first-, second-, and third-line analgesic; provide discharge instructions for patients to manage pain at home; and consider how a pain plan would change if the patient had a history of SUD. We scored the open-ended questions using the coding scheme found in the annotated knowledge assessment (Appendix H), which assigned 1 point for each correct piece of information in a given response. The postassessment included subjective questions eliciting learner feedback to improve future iterations of the seminar. Pre- versus postintervention data for the seminars were analyzed using paired Student *t* tests.[30]

Results

From February 2021 through March 2023, 19 fourth-year medical students enrolled in either the Emergency Medicine Bootcamp (n = 15) or the Emergency Medicine Capstone (n = 4) course and completed the pre- and postsession knowledge assessments (a total of four different course

Copyright © 2023 Sehdev et al. This is an open-access publication distributed under the terms of the Creative Commons Attribution license.

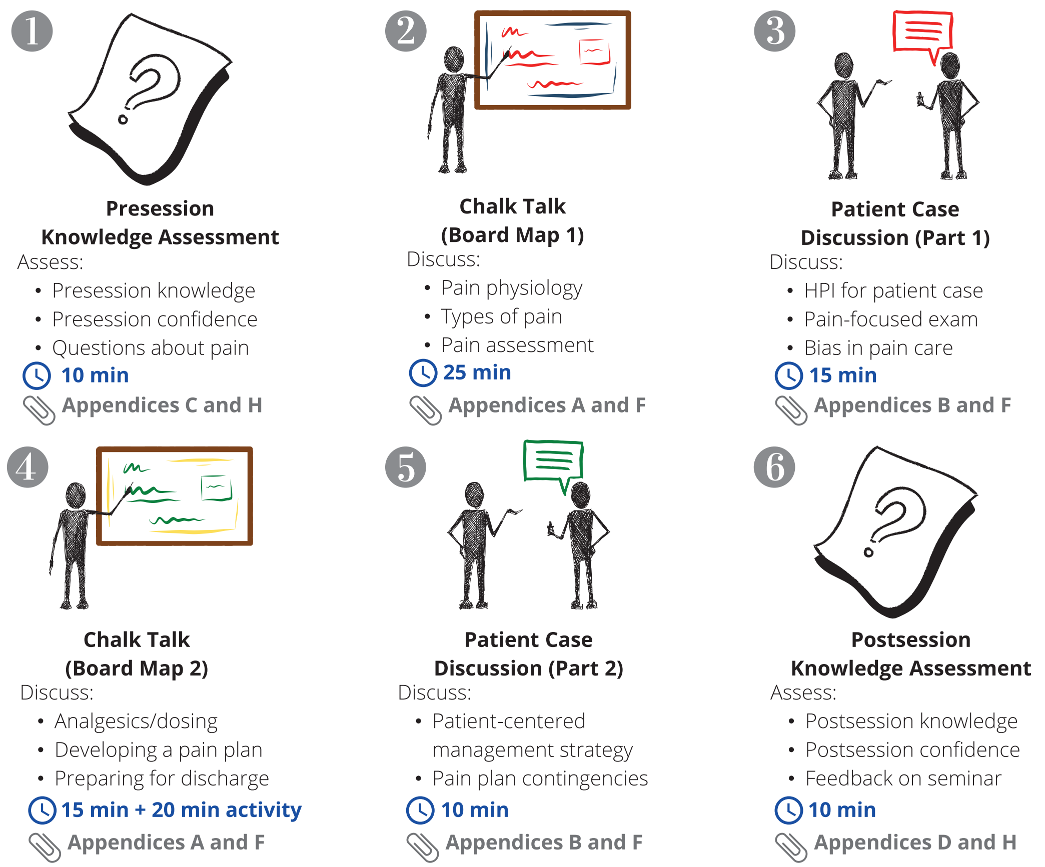

Figure 2. Graphical outline of the seminar's structure. The seminar can be broken down into six unique elements, including the administration of the pre- and postseminar knowledge assessments. The two board maps that anchor the seminar's 2.5 hours assist facilitators in capturing critical learning points while also serving as memory aids for the learners. Abbreviation: HPI, history of present illness.

offerings/learner enrollments). One hundred percent of learners who participated agreed to complete the assessments and did so. Ten of the 19 students applied into EM, and six enrolled in preparation for a medicine/pediatrics preliminary year.

Rated on a 5-point Likert scale (1 = *not at all confident,* 5 = *completely confident*), learner confidence in determining an appropriate or adequate pain plan to manage patient pain while in the ED increased significantly from a presession mean of 2.5 ($SD = 0.7$, $n = 19$) to a postsession mean of 4.0 ($SD = 0.5$, $p < .001$). Additionally, learner confidence in determining a second-line analgesic if the patient had minimal relief from the first attempt at pain management also increased significantly ($n = 19$; presession $M = 2.2$, $SD = 0.7$; postsession $M = 4.1$, $SD = 0.6$; $p < .001$).

We assessed change in learner knowledge after the seminar by comparing the scores from the five open-ended questions. Student scores demonstrated a significant increase in knowledge following the seminar. Out of 53 possible points, the average cumulative score before the seminar was 23.1 ($SD = 9.0$), while afterward, the average cumulative score was 36.6 ($SD = 6.3$, $p < .001$). Scores for each of the five open-ended questions individually increased significantly after the seminar (Table 1). Notably, as a result of the seminar, learners' ability to identify the types of pain experienced in a patient vignette and the breadth of appropriately chosen second- and third-line analgesic options increased significantly. Qualitatively, students commented that the seminar increased their "familiarity with doses of most commonly used pain medications" and that they now understood the "four different types of pain and potential ways to target [each]." Students identified individual learning gaps following the seminar by listing remaining questions; for example, "When should I consider medications like gabapentin for neuropathic pain?" and "When do I skip systemic pain medications and start an interventional pain management strategy such as a nerve block?"

Copyright © 2023 Sehdev et al. This is an open-access publication distributed under the terms of the Creative Commons Attribution license.

Table 1. Learner Scores on Pre- and Postsession Knowledge Assessments ($N = 19$)

Question	Maximum Possible Score	Presession *M* (*SD*)	Postsession *M* (*SD*)	*p*
1. Determining type of pain experienced by the patient	9	2.5 (2.1)	6.1 (1.8)	<.001
2. Creating a pain plan for the patient in case 1	15	8.1 (3.1)	11.5 (2.5)	<.001
3. Creating a pain plan for the patient in case 2	14	6.0 (3.7)	9.2 (3.2)	<.001
4. Designing discharge instructions for the patient in case 2	10	4.7 (2.5)	6.5 (2.0)	.01
5. Identifying necessary changes or considerations for a pain plan if a patient has substance use disorder	5	1.6 (1.3)	3.2 (1.5)	.014

The seminar was highly rated by the learners, with an average rating of 4.8 on a 5-point Likert scale (1 = *not at all helpful,* 5 = *extremely helpful*). All learners felt strongly that they would recommend repeating the session for other students in the future, with an average rating of 4.9 on a 5-point Likert scale (1 = *would not recommend at all,* 5 = *would highly recommend*).

Given the unique nature of applying a peer-peer and subsequently near-peer teaching model, we have also included observations and lessons learned by lead author Morgan Sehdev in Table 2. In future iterations at HMS, where other near-peers will fulfill the role of facilitator, we plan to develop facilitator assessments to further guide development and improvement of the seminar.

Discussion

We developed our interactive chalk talk and case-based seminar in response to the limited, fragmented, and variable teaching medical students and residents may receive on pain.[2-5] The seminar was well received by our learners while significantly enhancing both their confidence and their knowledge. As physicians continue to address ongoing public health crises related to pain care (e.g., the opioid epidemic or SUD) through medical education,[12] it remains necessary to bolster trainees' basic understanding of acute pain management.[5] Our seminar not only increases the availability of acute pain educational interventions but also provides ample resources to enhance future facilitators' comfort with pain education. This unique seminar gives learners the opportunity to review acute pain physiology, assessment, and analgesic pharmacology while introducing the novel idea of an acute pain plan from presentation through disposition, as well as the disparities within pain care.

Important lessons learned during the creation and implementation of this seminar include the following: (1) When utilizing the near-peer teacher approach, ensure that the student or trainee facilitator is supported by a more senior mentor both prior to and during the seminar, as this mentor can help the facilitator navigate the material and provide in-seminar clarification for any learner questions that extend beyond the facilitator resources or possible cognitive congruence; and (2) review the pharmacologic management table used on the second chalk talk board map with your institution's pharmacist to confirm

Table 2. Perspectives From the Peer-Peer Teacher

Theme	Reflections
Preparation	"Having gone through a majority of my medical school's curriculum by the time I was serving as the 'peer-peer' facilitator, I recognized my own weaknesses and gaps in pain education and pain management. Therefore, working through the creation of and the review of the facilitator guide not only helped me learn more about pain, but also feel adequately prepared to teach."
	"Practicing the chalk talk portion of the seminar ahead of time and recording it for my own review aided in my preparation and allowed me to make more cohesive transitions while it also highlighted how I could better record on/utilize the whiteboard."
Presentation	"During the presentation, I emphasized my motivations for teaching about acute pain. I also made sure to highlight the pieces of information I found to be most eye-opening or potentially practice changing (for myself) moving forward. From impromptu learner feedback, this was incredibly useful to everyone and brought a 'relatability' to the session—as stated by one of my classmate-learners."
	"Creating space for conversation during the activities and learner response sections of the seminar took away some of the mental burden of constantly being the one speaking but also allowed me to facilitate a space in which my classmates and learners stepped into the role of facilitator temporarily, which led to more vibrant discussion."
Moving forward	"After teaching this seminar multiple times and now progressing through my residency training, I feel much more confident of my pain management skills and often reflect on what I learned and taught when deciding when and how to treat patients' pain."

Copyright © 2023 Sehdev et al. This is an open-access publication distributed under the terms of the Creative Commons Attribution license.

that the medications, doses, and routes of administration are likely to be encountered or used in practice by your learners.

A strength of this intervention is the approach to acute pain demonstrated in the seminar's progression—a framework that fosters a stepwise discussion through types, characterization, and management of patients' pain using our novel acute pain plan approach. The acute pain plan and associated knowledge assessment were developed after recognizing the lack of a framework for helping trainees learn about and consider acute pain from presentation through disposition. This framework can be applied outside of the ED, such as in inpatient medical and surgical services. By encouraging trainees to anticipate the need for repeated dosing of analgesics or to consider second- or third-line analgesics, we can promote improved patient care, comfort, and outcomes.

Although innovative and filling a known educational need, our intervention had limitations: (1) We implemented our seminar at a single institution among a limited cohort of learners—it is encouraging that the results were significant among our small cohort, but they may not be generalizable to other institutions or learners; (2) our assessment strategy primarily evaluated students' reactions to and knowledge acquired during the seminar, as the assessments were not able to account for any possible behavior changes in practice; and (3) the seminar was limited to 2.5 hours and did not allow for a full discussion of adjunct medications that might be useful for certain types of pain (e.g., steroids, gabapentin, muscle relaxants) or interventional pain control and nerve blocks, which are increasingly common in the ED.

Moving forward, modifications will be made to the teaching materials to focus on the theory of pain ladders, pyramids, and/or multimodal approaches, especially as they pertain to the development of a pain plan. These modifications will be made in response to some of the learners' weaker responses to the postsession knowledge assessment. Furthermore, this seminar can be adapted and implemented among different learners. We anticipate that future iterations will allow other near-peer teachers to practice, prepare, and present the material. Further work with this seminar will continue to evaluate change in learners' knowledge while also seeking an evaluation strategy to measure behavioral changes, particularly among resident learners. For future resident learners, the addition of templated discharge pain plans for patients discharged home with acute pain will be critical. Adding adjunct simulation to the seminar may promote possible behavioral changes among future learners and move the seminar further up Bloom's taxonomy.[31] To assess knowledge retention or behavior changes, facilitators presenting this material to students who will also be evaluated in a clinical component of the course might consider including a question regarding pain management skills on a faculty postshift feedback form. Finally, this material would benefit from complementary seminars that address the acute pain management topics not covered in detail during the seminar, including nerve blocks, opioid conversions, and neuropathic pain-specific treatment.

This seminar ultimately addressed a curricular gap experienced by senior medical students, provided learners with an engaging near-peer chalk talk that enhanced their confidence in and knowledge of managing acute pain, and demonstrated a framework with which to manage a critically common complaint in medicine.

Appendices

A. Chalk Talk Board Maps.docx
B. Patient Case.docx
C. Presession Knowledge Assessment.docx
D. Postsession Knowledge Assessment.docx
E. Pocket Card.pdf
F. Facilitator Guide.docx
G. Facilitator Notes and Prereading.docx
H. Annotated Knowledge Assessment.docx

All appendices are peer reviewed as integral parts of the Original Publication.

Morgan Sehdev, MD: Third-Year Resident, Department of Emergency Medicine, Massachusetts General Hospital; ORCID: https://orcid.org/0000-0002-2981-0512

Jason Lewis, MD: Assistant Professor, Department of Emergency Medicine, Beth Israel Deaconess Medical Center; ORCID: https://orcid.org/0000-0003-4288-0045

Antje Barreveld, MD: Assistant Professor, Department of Anesthesiology, Newton-Wellesley Hospital; ORCID: https://orcid.org/0000-0003-1126-2797

Disclosures
None to report.

Funding/Support
None to report.

Prior Presentations
Sehdev M, Lewis J, Barreveld A. Demystifying acute pain: a chalk talk for medical students using a novel pain plan approach. Poster presented

Copyright © 2023 Sehdev et al. This is an open-access publication distributed under the terms of the Creative Commons Attribution license.

at: Society for Academic Emergency Medicine New England Regional Meeting; April 6, 2022; Worcester, MA.

Sehdev M, Lewis J, Barreveld A. Demystifying acute pain: a chalk talk for medical students using a novel pain plan approach. Poster presented at: Society for Academic Emergency Medicine Annual Meeting; May 10-13, 2022; New Orleans, LA.

Ethical Approval

The Harvard Medical School Program of Medical Education and Medical Education Institutional Review Board deemed further review of this project not necessary.

References

1. Yanni LM, McKinney-Ketchum JL, Harrington SB, et al. Preparation, confidence, and attitudes about chronic noncancer pain in graduate medical education. *J Grad Med Educ*. 2010;2(2):260-268. https://doi.org/10.4300/JGME-D-10-00006.1
2. Mezei L, Murinson BB; Johns Hopkins Pain Curriculum Development Team. Pain education in North American medical schools. *J Pain*. 2011;12(12):1199-1208. https://doi.org/10.1016/j.jpain.2011.06.006
3. Shipton EE, Bate F, Garrick R, Steketee C, Shipton EA, Visser EJ. Systematic review of pain medicine content, teaching, and assessment in medical school curricula internationally. *Pain Ther*. 2018;7(2):139-161. https://doi.org/10.1007/s40122-018-0103-z
4. Paziana K, Timpano E, Stolbach A. Designing and implementing emergency department pain management curriculum: a Delphi approach. *AEM Educ Train*. 2018;2(2):121-129. https://doi.org/10.1002/aet2.10092
5. Vadivelu N, Mitra S, Hines R, Elia M, Rosenquist RW. Acute pain in undergraduate medical education: an unfinished chapter! *Pain Pract*. 2012;12(8):663-671. https://doi.org/10.1111/j.1533-2500.2012.00580.x
6. Dotters-Katz S, Hargett CW, Zaas AK, Criscione-Schreiber LG. What motivates residents to teach? The *Attitudes in Clinical Teaching* study. *Med Educ*. 2016;50(7):768-777. https://doi.org/10.1111/medu.13075
7. Todd KH. A review of current and emerging approaches to pain management in the emergency department. *Pain Ther*. 2017;6(2):193-202. https://doi.org/10.1007/s40122-017-0090-5
8. Rupp T, Delaney KA. Inadequate analgesia in emergency medicine. *Ann Emerg Med*. 2004;43(4):494-503. https://doi.org/10.1016/j.annemergmed.2003.11.019
9. Axeen S, Seabury SA, Menchine M. Emergency department contribution to the prescription opioid epidemic. *Ann Emerg Med*. 2018;71(6):659-667.e3. https://doi.org/10.1016/j.annemergmed.2017.12.007
10. Lester P, Remolana R, Sandhu S, Scott J. Road map for opioid management in the inpatient setting: a structured approach to opioid selection and titration. *MedEdPORTAL*. 2016;12:10470. https://doi.org/10.15766/mep_2374-8265.10470
11. Fujita AW, LaRosa A, Carter A. Treating withdrawal and pain in inpatients with opioid use disorder: a brief educational intervention for internal medicine residents. *MedEdPORTAL*. 2021;17:11123. https://doi.org/10.15766/mep_2374-8265.11123
12. Sagin A, Kimberly SM, Farabelli JP, Schafer K, Kumar P, Uritsky TJ. Teaching pain management in serious illness in the era of the opioid epidemic: a team-based intervention. *MedEdPORTAL*. 2020;16:11006. https://doi.org/10.15766/mep_2374-8265.11006
13. Smith K. Introduction to pain management for third-year medical students team-based learning module. *MedEdPORTAL*. 2021;17: 11095. https://doi.org/10.15766/mep_2374-8265.11095
14. Barratt D, Tolchin R, Brown A, Segui D, Nausheen F. Neck pain OSCE: a 65-year-old patient presents to the emergency department with neck pain. *MedEdPORTAL*. 2015;11:10151. https://doi.org/10.15766/mep_2374-8265.10151
15. Riddell JC, Sawtelle S, Jhun P, et al. Low back pain in the emergency medicine department: a flipped classroom module. *MedEdPORTAL*. 2016;12:10458. https://doi.org/10.15766/mep_2374-8265.10458
16. Teo AR, Harleman E, O'Sullivan PS, Maa J. The key role of a transition course in preparing medical students for internship. *Acad Med*. 2011;86(7):860-865. https://doi.org/10.1097/ACM.0b013e31821d6ae2
17. Thomas PA, Kern DE, Hughes MT, Tackett SA, Chen BY, eds. *Curriculum Development for Medical Education: A Six-Step Approach*. 4th ed. Johns Hopkins University Press; 2022.
18. Mookherjee S, Beste LA, Klein JW, Wright J, eds. *Chalk Talks in Internal Medicine: Scripts for Clinical Teaching*. Springer; 2020.
19. Hall S, Harrison CH, Stephens J, et al. The benefits of being a near-peer teacher. *Clin Teach*. 2018;15(5):403-407. https://doi.org/10.1111/tct.12784
20. Kitzes JA, Savich RD, Kalishman S, et al. Fitting it all in: integration of 12 cross-cutting themes into a school of medicine curriculum. *Med Teach*. 2007;29(5):437-442. https://doi.org/10.1080/01421590701288564
21. Antman KH, Berman HA, Flotte TR, Flier J, Dimitri DM, Bharel M. Developing core competencies for the prevention and management of prescription drug misuse: a medical education collaboration in Massachusetts. *Acad Med*. 2016;91(10): 1348-1351. https://doi.org/10.1097/ACM.0000000000001347
22. Howard B. Students learn to treat pain, with and without opioids. Association of American Medical Colleges. October 2, 2018. Accessed July 6, 2023. https://www.aamc.org/news-insights/students-learn-treat-pain-and-without-opioids
23. Hauer KE, Boscardin C, Brenner JM, van Schaik SM, Papp KK. Twelve tips for assessing medical knowledge with open-ended questions: designing constructed response examinations in

Copyright © 2023 Sehdev et al. This is an open-access publication distributed under the terms of the Creative Commons Attribution license.

medical education. *Med Teach*. 2020;42(8):880-885. https://doi.org/10.1080/0142159X.2019.1629404

24. Pluta WJ, Richards BF, Mutnick A. PBL and beyond: trends in collaborative learning. *Teach Learn Med*. 2013;25(suppl 1): S9-S16. https://doi.org/10.1080/10401334.2013.842917
25. Platts-Mills TF, Esserman DA, Brown DL, Bortsov AV, Sloane PD, McLean SA. Older US emergency department patients are less likely to receive pain medication than younger patients: results from a national survey. *Ann Emerg Med*. 2012;60(2):199-206. https://doi.org/10.1016/j.annemergmed.2011.09.014
26. Chen EH, Shofer FS, Dean AJ, et al. Gender disparity in analgesic treatment of emergency department patients with acute abdominal pain. *Acad Emerg Med*. 2008;15(5):414-418. https://doi.org/10.1111/j.1553-2712.2008.00100.x
27. Pletcher MJ, Kertesz SG, Kohn MA, Gonzales R. Trends in opioid prescribing by race/ethnicity for patients seeking care in US emergency departments. *JAMA*. 2008;299(1):70-78. https://doi.org/10.1001/jama.2007.64
28. Graffam B. Active learning in medical education: strategies for beginning implementation. *Med Teach*. 2007;29(1):38-42. https://doi.org/10.1080/01421590601176398
29. Developing a pain control plan. American Cancer Society. Updated January 3, 2019. Accessed July 6, 2023. https://www.cancer.org/treatment/treatments-and-side-effects/physical-side-effects/pain/developing-a-pain-control-plan.html
30. Harpe SE. How to analyze Likert and other rating scale data. *Curr Pharm Teach Learn*. 2015;7(6):836-850. https://doi.org/10.1016/j.cptl.2015.08.001
31. Bloom BS, Krathwohl DR, Masia BB, eds. *Taxonomy of Educational Objectives.* David McKay; 1986.

Received: July 18, 2022
Accepted: May 29, 2023
Published: August 22, 2023

Copyright © 2023 Sehdev et al. This is an open-access publication distributed under the terms of the Creative Commons Attribution license.
